# Structure–property relationships of responsive doubly-threaded slide-ring polycatenane networks

**DOI:** 10.1039/d5sc05459a

**Published:** 2025-09-17

**Authors:** Guancen Liu, Jongwon Oh, Yuan Tian, Jerald E. Hertzog, Heyi Liang, Benjamin W. Rawe, Natsumi Nitta, Charlie A. Lindberg, Hojin Kim, Juan J. de Pablo, Stuart J. Rowan

**Affiliations:** a Department of Chemistry, University of Chicago Chicago IL 60637 USA stuartrowan@uchicago.edu; b Pritzker School of Molecular Engineering, University of Chicago Chicago IL 60637 USA jjd8110@nyu.edu; c Department of Chemical and Biomolecular Engineering, Tandon School of Engineering, New York University Brooklyn NY 11201 USA; d James Franck Institute, University of Chicago Chicago IL 60637 USA; e Department of Computer Science, Courant Institute of Mathematical Sciences, New York University New York NY 10012 USA; f Department of Physics, New York University New York NY 10003 USA; g Chemical Science and Engineering Division and Center for Molecular Engineering, Argonne National Laboratory 9700 S. Cass Ave. Lemont IL 60434 USA

## Abstract

Slide-ring polycatenane networks (SR-PCNs) are covalent polymer networks that contain interlocked doubly threaded rings that serve as additional topological constraints. These rings are catenated by the covalent polymer network, enabling them to slide along the polymer backbone between the covalent crosslinks. Herein, the SR-PCN synthesis is achieved by reacting a metal-templated doubly threaded pseudo[3]rotaxane (P3R) crosslinker with a chain extender and a covalent crosslinking moiety. The focus of this work is to explore the impact that monomer structure has on the SR-PCN synthesis, with the goal of increasing the reaction kinetics of the P3R to optimize ring incorporation in the network and minimize side reactions. It is shown that through monomer optimization it is possible to synthesize SR-PCNs with high gel fractions and ring content, allowing a detailed evaluation of the influence of the rings on the properties of these interlocked networks. Compared with control covalent networks and a tangled network, formed using a 1 : 2 metal–ligand complex, SR-PCNs exhibit enhanced swelling and frequency-dependent viscoelastic behavior, which are attributed to the motion of the rings. Molecular simulations of model interlocked networks elucidate the underlying mechanisms governing the mechanical behavior and provide insights into the structural changes induced by the rings. In addition, the responsive behavior of these SR-PCNs is explored upon exposure to stimuli that impact the ring mobility, such as changes in solvent, metalation, and protonation of the ligand moieties.

## Abstract 2

With optimized synthesis, slide-ring polycatenane networks exhibit distinct swelling and viscoelasticity arising from ring motion. Simulations elucidate these mechanisms, and ring mobility effects are revealed through metalation and protonation.

## Graphical abstract

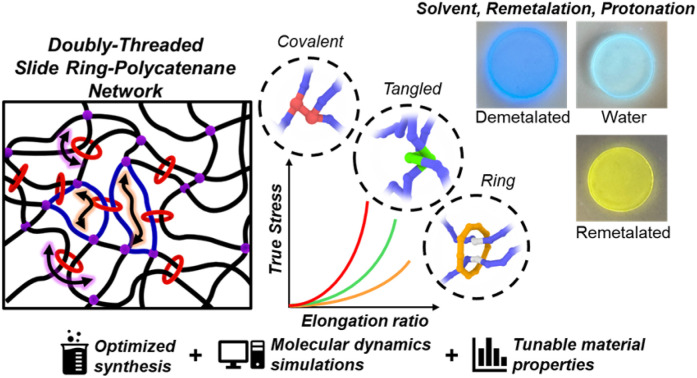

## Introduction

**Fig. 1:**
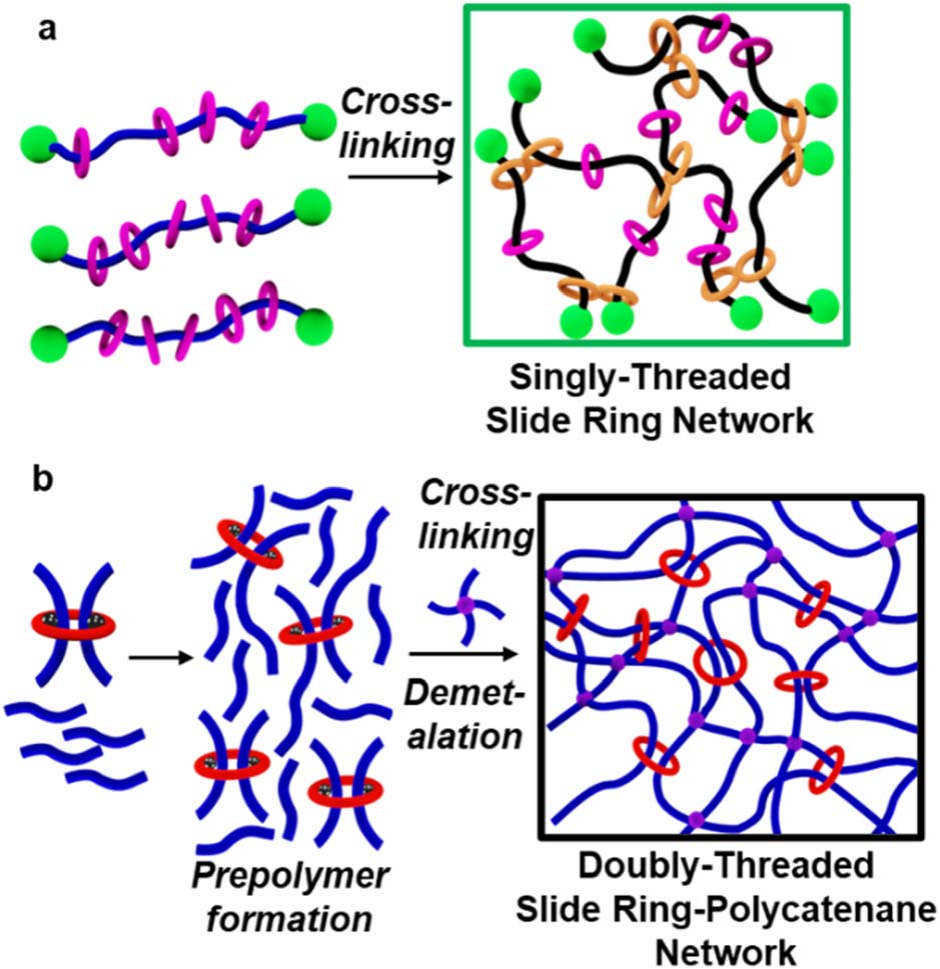
(a) Synthesis of singly-threaded slide-ring networks (SRN) by chemically crosslinking the rings of a polyrotaxane, resulting in figure-of-eight crosslinks. (b) Synthesis of slide-ring polycatenane network (SR-PCN) using a metal ion templated doubly-threaded pseudo[3]rotaxane (P3R) to react with an excess of chain extender followed by reaction with a covalent crosslink. After crosslinking, the metal ion is removed to yield the SR-PCN.

Mechanically interlocked molecules (MIMs) are a class of molecules characterized by the presence of the mechanical bond.^1–6^ The most prevalent types of MIMs are catenanes, which are interlocked macrocycles (rings), and rotaxanes, which consist of a ring threaded onto a dumbbell component.^7–9^ The presence of mechanical bonds, as opposed to covalent bonds, between components generally enhances the conformational freedom of the interlocked components, enabling access to distinct motions such as rotation, elongation, twisting, and sliding relative to their interlocked partner.^10–12^ Incorporating MIMs into polymeric materials allows access to mechanically interlocked polymers (MIPs).^13–15^ Among the family of MIPs, slide-ring networks (SRN, a subclass of polyrotaxanes) have been demonstrated to exhibit remarkable physicochemical and mechanical properties as a result of the mobility of the rings along the polymer backbone.^16,17^ This mobility leads to enhanced toughness,^18,19^ increased extensibility,^20–23^ stimuli-responsive actuation,^24^ shape memory,^25^ stress relaxation,^12^ and rapid energy dissipation.^26^ Typical synthetic approaches to SRNs involve crosslinking the rings on a polyrotaxane to form figure-of-eight crosslinks (Fig. 1a),^17,18,21,27^ or using a polymer to crosslink the rings.^19,22,23,28,29^ Most SRNs reported to date are singly-threaded (one polymer chain threaded through a ring). It is possible to access more complex interlocked structures, such as doubly-threaded (dt) MIMs, where two dumbbell components are threaded through one ring, in high yield,^30–33^ which has subsequently led to a few reports of dtMIPs.^34–38^ A related class of MIPs is polycatenanes, where the mechanical bond is the result of interlocked rings (*i.e.*, a catenane moiety).^39–41^ To date, the majority of polycatenane networks have focused on systems that have a singly-threaded [2]catenane as their interlocked component.^42–49^

**Fig. 2:**
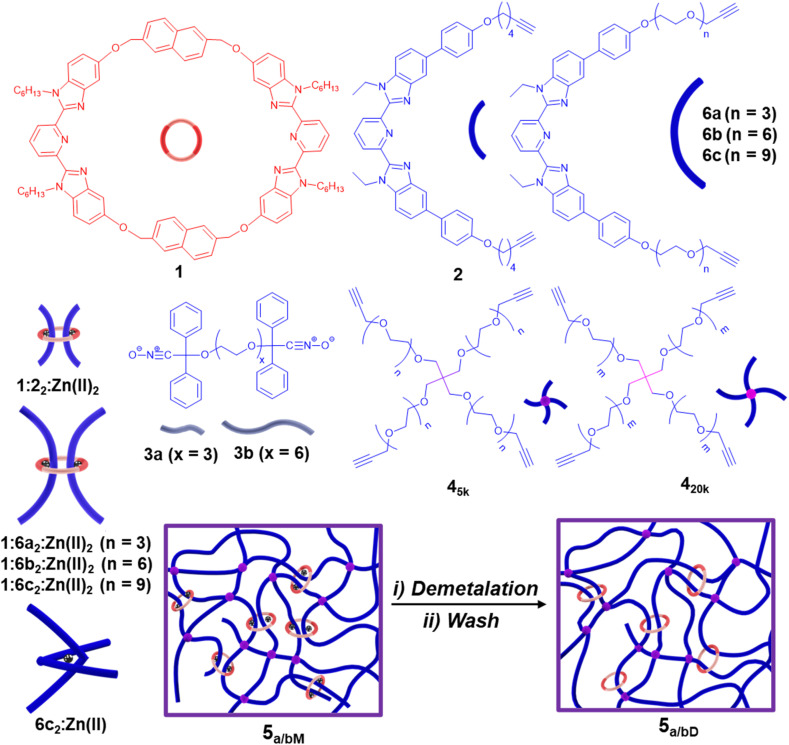
Structure of the components used in the synthesis of SR-PCN *via* nitrile-oxide/alkyne cycloaddition polymerization – the doubly-threaded pseudo[3]rotaxanes (P3Rs) 1 : 2_2_:Zn(ii)_2_ and 1:6a-c_2_:Zn(ii)_2_, covalent tetra-alkyne PEG crosslinks 4_5k_ and 4_20k_ (M_n_ = 5 kg mol^−1^ and 20 kg mol^−1^, respectively), 4-arm tetra-alkyne 6c_2_:Zn(ii) formed by metal–ligand assembly, and *bis*-nitrile-oxide chain extenders 3a and 3b. Copolymerization of 1 : 2_2_:Zn(ii)_2_ and 4_5k_ with 3a yielded a series of SR-PCNs 5_a/bM_ by varying the ratio of P3R and covalent crosslink, and upon base treatment the demetalated 5_a/bD_.

Networks with multiply-threaded catenane moieties include Olympic gels and kinetoplast DNAs.^48,50–56^ A subclass of multiply-threaded catenane-containing networks are the slide-ring polycatenane networks (SR-PCN, Fig. 1b). These SR-PCNs contain crosslinking doubly-threaded (dt) rings embedded within a percolating covalent network.^57^ The synthetic route to such networks employed a dt-pseudo[3]rotaxane (P3R) 1:2_2_:Zn(ii)_2_ (Fig. 2, *a* = 0–30 mol%) crosslinker, formed from the self-assembly of a ditopic 2,6-bis(*N*-alkyl-benzimidazolyl)pyridine (BIP) ring^58^1 and two alkyne endcapped linear BIP-containing threads 2 with a transition metal ion, Zn(ii).^59,60^1:2_2_:Zn(ii)_2_ was polymerized *via* a catalyst-free nitrile-oxide/alkyne cycloaddition in the presence of a covalent tetra-alkyne poly(ethylene glycol) (PEG) crosslinker 4_5k_ (M_n_ = 5 kg mol^−1^) (Fig. 2, *b* = 100–*a* mol%) and a *bis*-nitrile oxide chain extender 3a (Fig. 2). After curing, the networks were washed to remove the sol fraction, yielding the metalated gel 5_a/bM_. The demetalated gel 5_a/bD_ (Fig. 2, M corresponds to metalated gel, and D corresponds to demetalated networks) was accessed using tetrabutylammonium hydroxide (TBAOH). Studies showed that *ca.* 30% of the macrocycle was retained within the network after demetalation and washing. Furthermore, the gel fraction (GF, wt%) of 5_a/bD_ dropped significantly when introducing more of the P3R crosslink in the synthesis. Taken together, these results suggest that the P3R crosslinker was not incorporated into the network as efficiently as the covalent crosslinker 4_5k_, which limited the ability to carry out detailed mechanical studies to evaluate the impact of the ring on the properties of these new networks. Thus, with the goal of gaining a better understanding of the properties of this class of MIP networks and how the incorporation of the mobile ring impacts their properties, the work herein is focused on (1) improving the synthesis of the SR-PCN by redesigning the system to allow for a more efficient incorporation of the P3R into the network, (2) preparing a series of control networks, (3) carrying out studies on their mechanical properties, and (4) interpreting the experimental studies through detailed simulations of molecular models of the materials.

## Results and discussion

**Fig. 3:**
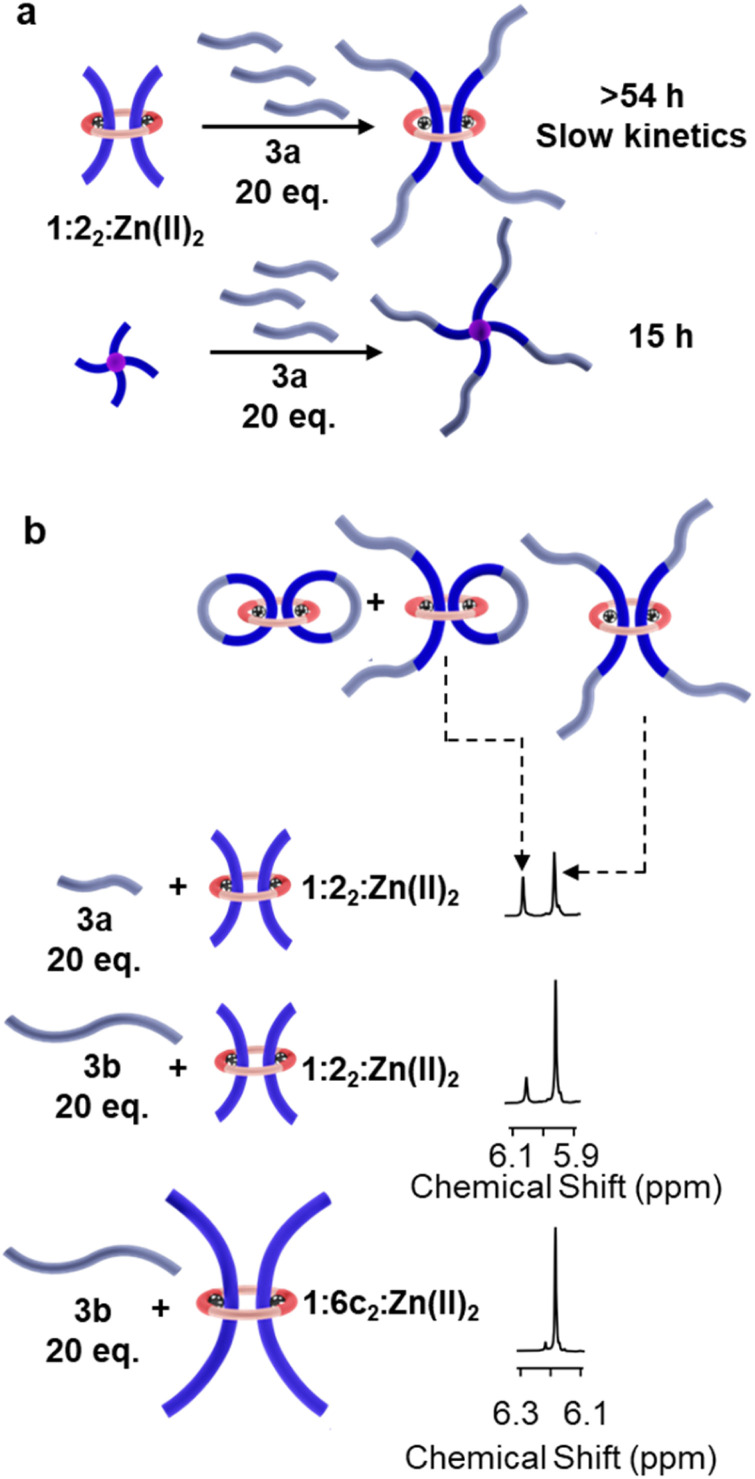
Study of the reaction between different crosslinks and the chain extenders. (a) Schematics of reaction of 1 : 2_2_:Zn(ii)_2_ and 4_5k_ reacting with 3a. (b) Region of the ^1^H NMR spectra (500 MHz, 5% CD_3_CN in CDCl_3_, 298 K) corresponding to the H_isox_ protons for 1 : 2_2_:Zn(ii)_2_ reacting with 3a and 3b, as well as 1:6c_2_:Zn(ii)_2_ reacting with 3b. Chain extenders are shown in light blue for clarity.

It was hypothesized that the limited incorporation of the P3R (1:2_2_:Zn(ii)_2_) in the prior SR-PCN studies was a result of the slower reaction kinetics of the *bis*-nitrile oxide 3a reacting with 1:2_2_:Zn(ii)_2_ (Fig. S1–S8) relative to the *tetra*-PEG alkyne 4_5k_. Thus, initial investigations focused on understanding the reactivity of the different components. As such, 1:2_2_:Zn(ii)_2_ and 4_5k_ were reacted with excess 3a (20 equiv.) and monitored *via* proton nuclear magnetic resonance (^1^H-NMR) spectroscopy (Fig. S9 and S10). While 4_5k_ took 15 h to fully react (*k* = 1.2 × 10^−3^ M^−1^ s^−1^), 1:2_2_:Zn(ii)_2_ required much longer (>54 h) for the reaction to go to completion (*k* = 2.9 × 10^−4^ M^−1^ s^−1^) (Fig. 3a). In addition, while the reaction with 4_5k_ shows only one signal in the isoxazole proton (H_isox_) region (*ca*. 5.96 ppm) (Fig. S9), the reaction with 1:2_2_:Zn(ii)_2_ showed the presence of two peaks with a second peak (*ca*. 6.06 ppm, 35% of the total H_isox_ by ^1^H-NMR spectroscopy) that is shifted downfield relative to the major signal (*ca*. 5.96 ppm) (Fig. 3b and S10). In a separate reaction using 1:2_2_:Zn(ii)_2_ and 3a (2 equiv.), this downfield-shifted signal becomes the predominant signal (66% of the total H_isox_) (Fig. S11). This suggests that the downfield signal comes from the cyclization of one of the threads in 1:2_2_:Zn(ii)_2_ with 3a to form a catenane. In fact, after demetalation of this reaction mixture, ^1^H-NMR shows the presence of upfield-shifted signals corresponding to the pyridyl protons consistent with interlocked BIP-based compounds^39–41,59–62^ (Fig. S12) and matrix-assisted laser desorption/ionization-time of flight (MALDI-TOF) mass spectrometry confirmed the presence of [2] and [3]catenanated species (Fig. S13). Taken together, these results not only show that 1:2_2_:Zn(ii)_2_ reacts with 3a more slowly than 4_5k_, but also that it can form catenanated byproducts that hinder network formation.

To limit the catenane side reaction, the *bis*-nitrile oxide 3b was synthesized with a longer hexaethylene glycol core (Fig. 2 and S14–S16). When reacting 3b (20 equiv.) with 1:2_2_:Zn(ii)_2_, the downfield shifted signal in the isoxazole proton decreased to *ca.* 18% of the total Hisox (Fig. 3b and S17–S20), indicating less catenane formation with the longer chain. However, the reaction rate did not change significantly. Hypothesizing that steric considerations are the reason for the relatively slow reaction rate of 1:2_2_:Zn(ii)_2_ with 3a/3b, it was decided to access P3Rs with BIP thread components 6a–c that have oligoethylene glycol extensions (*n* = 3, 6, or 9, respectively) between the ligand and alkyne (Fig. 2 and S21–S29). With these components in hand, a series of dtP3Rs with macrocycle 1 and the different threads 6a–c were self-assembled with Zn(ii) ions (Fig. 2 and S30–S38). Diffusion ordered spectroscopy (DOSY) confirmed the formation of larger P3R assemblies with the longer threads (Fig. S39–S48 and Table S1). The reaction of 3b (20 equiv.) with 1:6a_2_:Zn(ii)_2_ resulted in faster reaction kinetics relative to 1:2_2_:Zn(ii)_2_ (*k* = 7.9 × 10^−4^ M^−1^ s^−1^) (Fig. S49). The intensity of the downfield shifted byproduct signals (*ca.* 6.28–6.22 ppm) was also reduced to 15% of the major signal at *ca.* 6.20 ppm. Increasing the length of the spacer further revealed an additional enhancement in reaction kinetics. Reacting 1:6b_2_:Zn(ii)_2_ or 1:6c_2_:Zn(ii)_2_ with 3b (20 equivs.) resulted in the reactions going to completion in *ca.* 15 h (*k* = 1.2 × 10^−3^ M^−1^ s^−1^) (Fig. S50 and S51). Furthermore, ^1^H-NMR spectroscopy showed that the catenane byproduct signal was further reduced to 7% for 1:6b_2_:Zn(ii)_2_ and 5% for 1:6c_2_:Zn(ii)_2_ relative to the major signal (at *ca.* 6.20 ppm) (Fig. S50–S54). A summary of the reaction times and the percent of catenane formed when reacting the different P3Rs with 3b is summarized in Fig. S55 and S56.

**Fig. 4:**
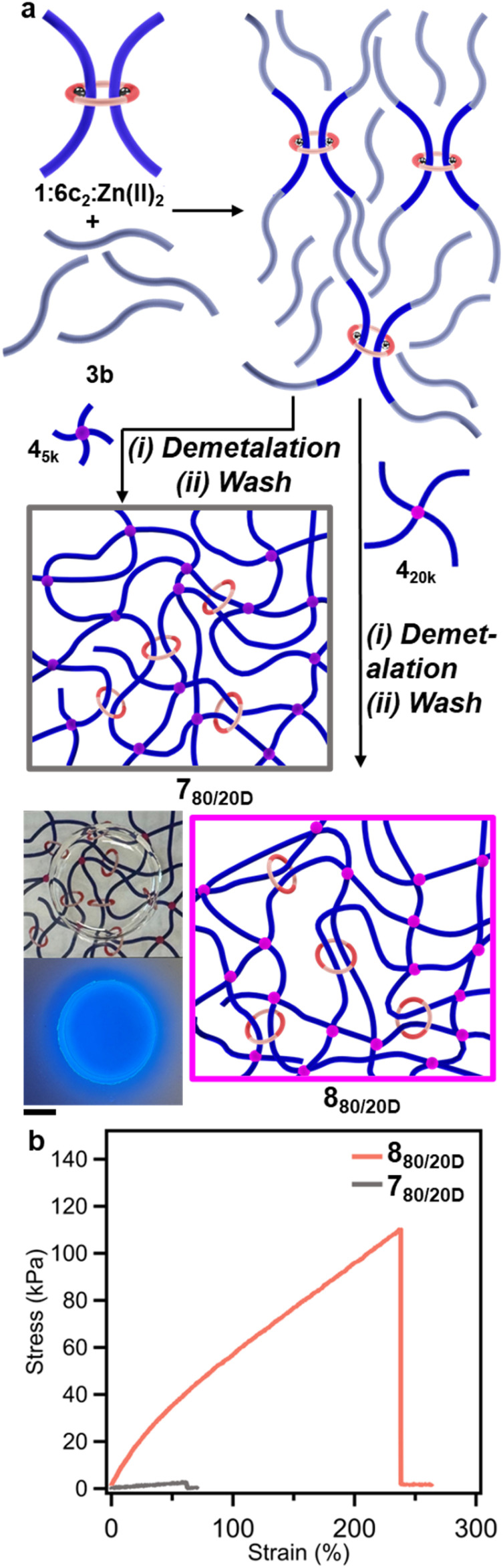
(a) Schemes showing the synthesis of SR-PCNs 7_80/20D_ and 8_80/20D_ using P3R 1:6c_2_:Zn(ii)_2_ to react with excess 3b followed by addition of the crosslink 4_5k_ or 4_20k_, respectively. Demetalation results in an optically clear film that fluoresces blue under 365 nm UV light, on the account of the free ligand. 8_80/20D_ pictured, 1 cm scale bar. (b) Stress–strain curves of swollen 7_80/20D_ and 8_80/20D_ in *N*-methyl-2-pyrrolidone (NMP) with a strain rate of 5 mm min^−1^.

Based on these studies, the synthesis of SR-PCNs was explored using 3b and 1:6c_2_:Zn(ii)_2_ (Fig. 4a). The P3R crosslinker 1:6c_2_:Zn(ii)_2_ (20 mol%) was reacted with an excess of 3b (200 mol%) before adding 4_5k_ (80 mol%) to yield the SR-PCN 7_80/20M_ (Fig. 4a and S57, see SI for details). After demetalation and washing, the gel fraction (GF) of 7_80/20D_ was higher than that observed for 5_80/20D_ (*ca.* 96% *vs. ca.* 83%).^57^ To calculate the amount of macrocycles retained in the 7_80/20D_ film, ^1^H-NMR analysis of the soluble fractions obtained from the washing and demetalation steps was carried out to determine how much macrocycles could be extracted from the film (eqn (S4), see SI for details). The data shows that *ca.* 19% of the macrocycle added to the reaction mixture was extracted from the 7_80/20D_ film, suggesting that the amount of ring retained in the network is over 80% (see SI for details), significantly higher than had been previously reported (29%).^57^ The swelling ratio (*Q*, vol%, see SI for details) of 7_80/20D_ is 1100 vol% in *N*-methyl-2-pyrrolidone (NMP), and the resulting gels are relatively weak/brittle (Fig. 4b). To access tougher materials, a higher molecular weight PEG crosslinker 4_20k_ (Fig. 2) (M_n_ = 20 kg mol^−1^) was reacted with 3b and 1:6c_2_:Zn(ii)_2_ (20 mol%) to synthesize SR-PCN 8_80/20D_ (Fig. 4a, S58 and S59, see SI for details). It was found that the swollen 8_80/20D_ (970 vol% in NMP) is a much tougher material relative to 7_80/20D_, exhibiting a significantly larger stress-at-break (110 kPa for 8_80/20D_*vs.* 3 kPa for 7_80/20D_) and strain-at-break (220% for 8_80/20D_*vs.* 60% for 7_80/20D_) in tensile tests (Fig. 4b).

**Fig. 5:**
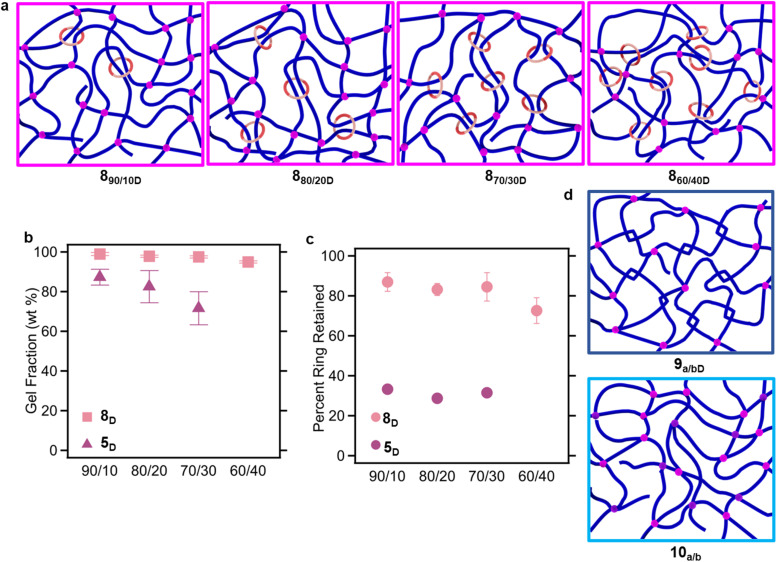
(a) Schematic of SR-PCN 8_a/bD_ with increasing number of rings (*b* = 10–40 mol%). (b) Comparison of the gel fraction (GF) of the synthesized SR-PCNs 8_a/bD_ and 5_a/bD_. Data on 5_a/bD_ from prior work.^57^ (c) Percent of ring retained in the network 8_a/bD_, relative to that of networks 5_a/bD_ (error bars omitted for clarity). Data of 5_a/bD_ from prior work.^57^ (d) Schematic of the two control networks 9_a/bM_ and 10_a/b_ synthesized from 3b with different ratios of the crosslinkers 4_20k_ and 6c_2_:Zn(ii) or 4_5k_, respectively. 9_a/bM_ was treated with TBAOH to yield 9_a/bD._

Having optimized the reaction components to access the SR-PCNs, the next step was to synthesize SR-PCNs with varying amounts of the P3R crosslinker. To that end, different ratios of the covalent crosslinker (4_20k_ from 90 mol% to 60 mol%) and the P3R crosslinker (1:6c_2_:Zn(ii)_2_ from 10 mol% to 40 mol%) were reacted with 3b to yield a series of SR-PCNs (8_a/bM_), where a and *b* are the relative mole percents of 4_20k_ and 1:6c_2_:Zn(ii)_2_ used in the network synthesis, respectively (Table S2, see SI for details). After extensive washing with chloroform, 8_a/bM_ films exhibited high GFs (>97%, Fig. S60) and after demetalation, the resulting 8_a/bD_ (Fig. 5a) retained their high GFs (>95%, Fig. 5b). The amounts of macrocycle and thread in the soluble fractions were quantified by ^1^H-NMR (Fig. S61, S62, and Table S3, see SI for details), allowing for determination of the amount of rings retained in the 8_a/bD_ films to be >80% for *b* = 10–30 mol% and *ca.* 73% for *b* = 40 mol% (Fig. 5c).

To better understand the impact of the slide-ring (SR) in the SR-PCN, two control networks were synthesized. A series of BIP-containing networks formed with metallosupramolecular crosslinking units (Fig. 2 and S63–S66) were synthesized *via* the copolymerization of 3b with varying ratios of 4_20k_ and the tetra-alkyne 6c_2_:Zn(ii), formed by self-assembly of two 6c with zinc bistriflimide (Zn(NTf_2_)_2_). The resulting networks 9_a/bM_ (*a* and *b* are the mole percentage of 4_20k_ and 6c_2_:Zn(ii) repsectively) were washed and demetalated (Fig. S67–S69, and Table S4, see SI for details). These gels are termed here tangled networks 9_a/bD_ (Fig. 5d), as the supramolecular crosslink has the potential to yield additional “trapped” chain entanglements, as has been shown by Zhukhovitskiy and coworkers.^63,64^ The amount of thread in the soluble fraction of 9_a/bD_ was quantified by ^1^H-NMR (Table S3, see SI for details) and showed that >94% of the BIP moieties are retained in the 9_a/bD_ films. A second set of control networks is the fully covalent networks 10_a/b_, which were synthesized by copolymerizing varying ratios of 4_20k_ and 4_5k_ (where *a* and *b* are the mole percentages of 4_20k_ and 4_5k_ crosslinkers, respectively) with 3b (Fig. 5d, S70 and S71, see SI for details). 4_5k_ was used as the control replacement for 1:6c_2_:Zn(ii)_2_ on account of their similar molecular weight. Both 9_a/bD_ and 10_a/b_ showed similarly high GF to 8_a/bD_ (Fig. S72 and S73). A summary of all network compositions and their naming scheme is shown in Table 1.

**Table 1:** Component composition used in the synthesis of the networks

Network^a^	Component *a* (mol%)	Component *b* (mol%)
SR-PCN 7_a/bD_	4_5k_ (80 mol%)	1:6c_2_:Zn(ii)_2_ (20 mol%)
SR-PCN 8_a/bD_	4_20k_ (90–60 mol%)	1:6c_2_:Zn(ii)_2_ (10–40 mol%)
Tangled 9_a/bD_	4_20k_ (90–60 mol%)	6c_2_:Zn(ii) (10–40 mol%)
Covalent 10_a/b_	4_20k_ (100–60 mol%)	4_5k_ (0–40 mol%)

a M corresponds to metalated gels and D corresponds to demetalated gels.

**Fig. 6:**
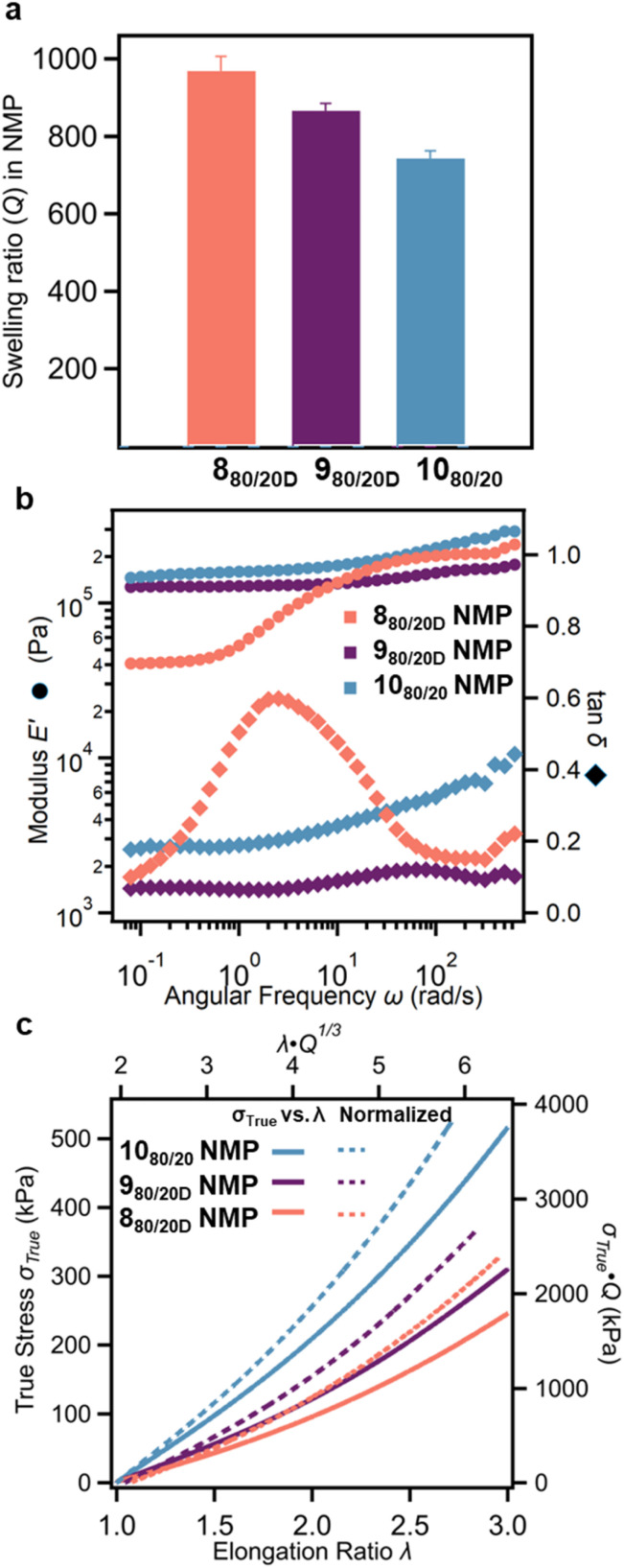
(a) Swelling ratio (Q) of SR-PCN 8_80/20D_, 9_80/20D_, and 10_80/20_ in NMP. (b) Storage moduli E′ and loss factor tan *δ* of 8_80/20D_, 9_80/20D_, and 10_80/20_ in NMP by SAOC. (c) True stress-elongation ratio curves of swollen 8_80/20D_, 9_80/20D_ , and 10_80/20_ in NMP (solid lines). The elongation ratio, *λ*, is defined as the ratio of the extended length, *L*, to its original length, *L*_0_, given by *λ* = *L*/*L*_0_ (strain rate of 20% min^−1^ with *λ* limited to 3, before the material breaks). Dotted lines show after normalizing by the swelling ratio (*σ*_true_·*Q* and *λ*·*Q*^1/3^).

With the three different classes of networks (8–10) in hand, the next step was to explore how the networks with dtSR crosslinks (8_a/bD_) compared to the control networks (9_a/bD_ and 10_a/b_). As such, 8_a/bD_, 9_a/bD_, and 10_a/b_ were swollen in NMP. The swelling ratio, *Q*, of all the SR-PCNs is higher than that of the corresponding 9_a/bD_ and 10_a/b_ gels (Fig. S74 and S75). For example, the *Q* of 8_80/20D_ (*ca.* 970%, Fig. 6a) is higher relative to 9_80/20D_ (*ca.* 870%) and 10_80/20_ (*ca.* 740%). This is consistent with the mobility of the SR crosslinks in 8_a/bD_, allowing greater swelling, as has been shown in other SRN architectures.^17,61^ Small-amplitude oscillatory compression (SAOC) experiments were conducted to study the frequency-dependent viscoelastic response of the three different networks in their equilibrium swollen states. Within the measured frequency regime, both 9_80/20D_ and 10_80/20_ exhibit no frequency dependence as would be expected for covalent networks. However, 8_80/20D_ showed two plateaus in the storage moduli (*E*′), one at high frequencies (*ω* > 100 rad s^−1^) and a second at low frequencies (*ω* < 1 rad s^−1^). At the higher frequencies, the storage modulus of 8_80/20D_ is *ca.* 150 kPa, similar to 9_80/20D_ and 10_80/20_ (Fig. 6b). However, the plateau storage modulus of 8_80/20D_ at low frequency (*ω* < 1 rad s^−1^) decreases to 45 kPa. A peak is observed in the loss factor (tan *δ*) at a frequency (*ω* = 3 rad s^−1^) consistent with this transition in *E*′ for 8_80/20D_ and is absent from 9_80/20D_ and 10_80/20_ (Fig. 6b). This transition is consistent with the sliding transition that has been observed before by Ito and co-workers in other classes of SRNs.^65–68^ The data is consistent with the SR crosslinks behaving akin to fixed crosslinks at high frequencies, while at low frequencies, the polymers have the time to slide through rings, resulting in a softer material. The frequency sweep of the other SR-PCNs in the series also shows a frequency-dependent transition that leads to a lower plateau storage modulus at low frequency and a relaxation peak in tan *δ* (Fig. S76 and S77). No such transition was observed in any of the control networks 9_a/bD_ and 10_a/b_ (Fig. S78 and S79).

Uniaxial tensile tests were conducted to further probe the mechanical properties of these gels. Gel 8_80/20D_ exhibited lower true stress (*σ*_true_, calculated from the equation *σ*_true_ = *σ*_eng_ × (1 + *ε*_eng_) where *σ*_eng_ and *ε*_eng_ are engineering stress and strain, respectively) across the measured elongation ratio range (*λ* = 1–3) than 9_80/20D_ and 10_80/20_ with the same percentage of the different crosslinks (Fig. 6c). Of course, the tensile properties of the gels are also related to their *Q*, with the more swollen gels yielding softer networks. However, after normalizing the effect of *Q* by *σ*_true_·*Q* and *λ*·*Q*^1/3^,^69^8_80/20D_ was still a softer network than either 9_80/20D_ or 10_80/20_ (Fig. 6c). The other 8_a/bD_ series also showed lower true stress than the corresponding control gels (Fig. S80 and S81), highlighting the impact of the dtSRs in the network.

**Fig. 7:**
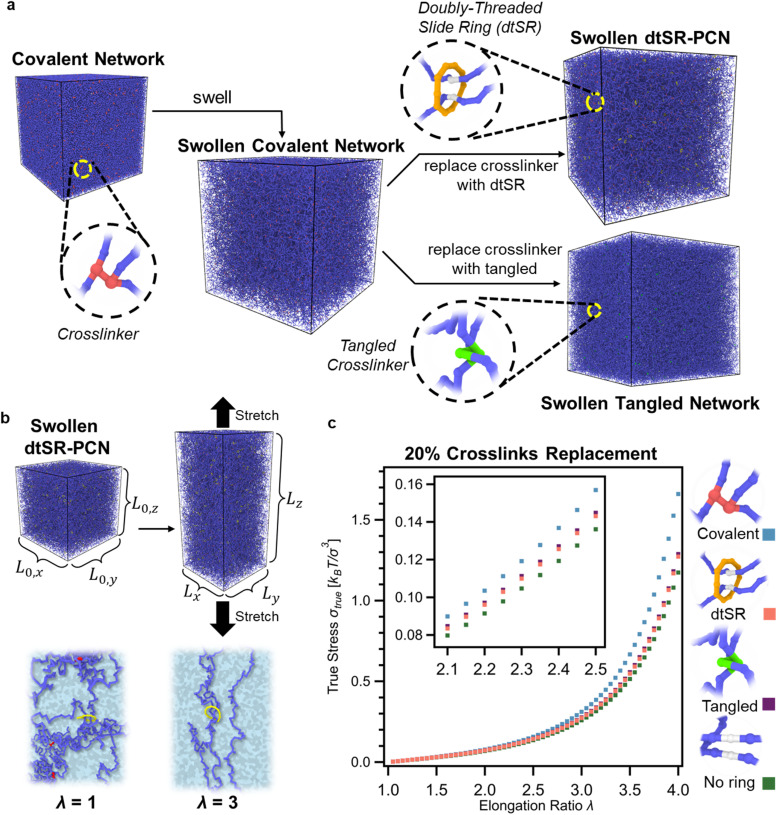
(a) Snapshots from coarse-grained molecular dynamics simulations illustrating polymer networks with different crosslinking architectures. The covalent polymer network is randomly crosslinked by precursor chains (blue) with a degree of polymerization of 1025 and with network strands composed of an average of 150 bonds between crosslinking bonds (red). The doubly threaded SR-PCN replaces conventional crosslinking bonds with doubly threaded slide rings (SRs, yellow). The tangled polymer network incorporates entanglement-like structures (green) as the crosslinking substitutes. In addition to these networks, another network was modeled, which maintains the same topology as the SR-PCN but lacks actual ring crosslinks at the potential crosslinking points (white), resulting in a network (termed no-ring network) with effectively lower crosslink density. (b) Illustration of uniaxial deformation of swollen SR-PCN, accompanied by snapshots of the SR-PCN structures in the undeformed (*λ* = 1) and stretched (*λ* = 3) states. (c) True stress-elongation curves obtained by the computer simulation of the polymer networks with different crosslinking architectures: covalent network (blue), SR-PCN (coral), tangled network (purple), and no-ring network (olive). The insert shows a magnified view of the dependence of true stress on elongation ratio in the range of *λ* = 2.1 and 2.5.

**Fig. 8:**
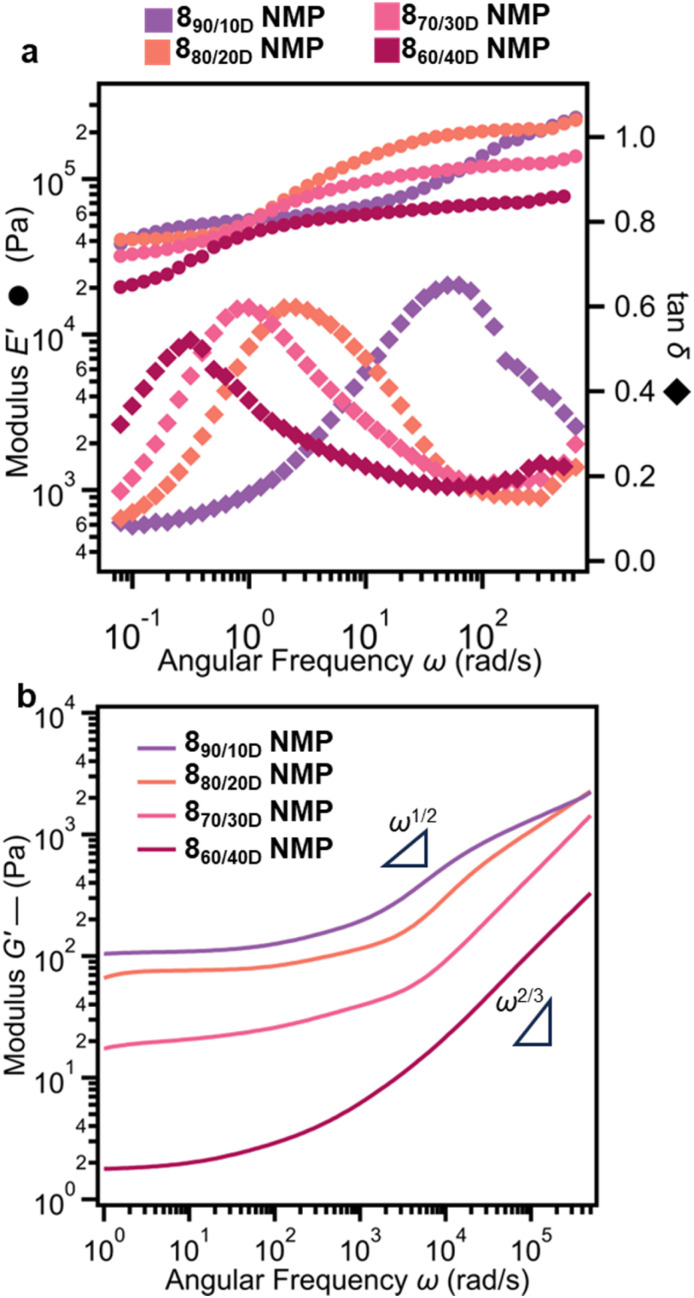
Effect on viscoelastic properties of the SR-PCNs 8_a/bD_ with varying percentages of SR crosslinks. (a) Compression storage moduli *E*′ and loss factor tan *δ* of 8_a/bD_ in *N*-methyl-2-pyrrolidone (NMP) by SAOC. (b) Shear storage moduli *G*′ of 8_a/bD_ in NMP by dynamic light scattering shear rheology.

To better understand the mechanisms underlying the properties of the differently crosslinked networks, molecular dynamics simulations of a coarse-grained model of the networks were performed under swollen conditions (Fig. 7a). The models consist of spherical interaction sites connected by springs and include covalent crosslinks and rings. More specifically, both the dtSR-PCN and the tangled network have 20% of the covalent crosslinks replaced with dtSRs and entangled-like structures, respectively. These network configurations are derived from the randomly crosslinked covalent networks with linear strands, each comprising 150 bonds between crosslinking points, denoted as *n*_*x*_ = 150, thereby preserving similar topologies across the different network types (Fig. 7a and S82).^70^ Upon deformation, the evolution of the dtSR and its two associated network strand conformations revealed that the SRs situated on the network strands aligned with the deformation direction exhibited larger displacements relative to those oriented otherwise, indicating enhanced mobility of those rings under tensile stress (Fig. 7b and S83). Furthermore, stress-elongation curves from deformation simulations of the different crosslinked networks show that the covalent network exhibits the highest stress compared to both the SR-PCN and tangled networks. Notably, the tangled network demonstrates slightly higher stress than the SR-PCN as a result of reduced flexibility inherent in its entangled-like structures. These findings are in good agreement with the experimental true stress-elongation behavior (Fig. 7c).^71^ Finally, by removing the same fraction and locations of crosslinking bonds from the covalent networks, mirroring the replacements made in the SR-PCN and tangled networks, a network akin to the SR-PCN but lacking rings was constructed. This no-ring network has a reduced crosslink density with longer strands between the crosslinks, which results in a softer gel.^69,70^ With a better understanding of how different network architectures impact the gel properties, the next step was to explore the effect the amount of SR crosslinks has on these properties. In the SR-PCN 8_a/bD_ series, replacing the covalent crosslinks with SR crosslinks results in an increase in *Q* to a greater degree than that of corresponding tangled gels 9_a/bD_ despite the same BIP-PEG polymer backbone for 8_a/bD_ and 9_a/bD_ (Fig. S74). From the SAOC frequency sweep of the SR-PCN series, the value of *E*′ at both high-frequency and low-frequency ranges decreases (Fig. 8a) when more SR crosslinks replace the covalent crosslinks, consistent with a larger mesh size. This contrasts with the covalent series (10_a/b_), where *E*′ increases as more of the lower molecular weight 4_5k_ crosslinking units are added as they reduce the molecular weight between crosslinks and therefore the mesh size (Fig. S84). Interestingly, in the SR-PCNs, the frequency for the sliding transition exhibits a strong dependence on the number of SR crosslinks in the series of 8_a/bD_. With an increasing number of rings, the sliding transition (peak in tan *δ*) shifts to lower frequencies (Fig. 8a). This observation would be consistent with a larger mesh size with more SR crosslinks, leading to a longer characteristic length scale.

With an interest in probing these interlocked networks over a broad range of time scales, dynamic light scattering (DLS) microrheology^72–74^ was used to explore the viscoelastic behavior of 8_a/bD_ across time scales (*ω* ≤ 500 000 rad s^−1^) beyond traditional rheological experiments (*ω* ≤ 1000 rad s^−1^). This was achieved by adding trimethylsilyl-functionalized silica particles (*D* = 500 nm) during the syntheses of these networks (see SI for details). At low to intermediate frequencies (*ω* ≤ 1000 rad s^−1^, longer time scale) in the DLS microrheology, the SR-PCNs showed a plateau storage shear modulus *G*′ with a weak frequency dependence on account of the polymers constrained by the matrix. At these time scales 8_a/bD_, 9_a/bD_, and 10_a/b_ (Fig. 8b and S85–S89) showed similar trends in storage modulus between DLS and SAOC rheology. At short time scales (*ω* ≥ 1000 rad s^−1^), DLS microrheology shows that the *G*′ increases with frequency and the particles probe faster relaxation modes of the rings and subsections of the polymers in these networks. The SR-PCNs with fewer ring crosslinks (8_90/10D_ and 8_80/20D_) exhibit characteristic Rouse-model (G* ∼ *ω*^1/2^) scaling behavior suggesting that the polymer chains in these networks behave more akin to concentrated polymer solutions.^72,74^ In contrast, the SR-PCNs with higher ring crosslinks (8_70/30D_ and 8_60/40D_) show Zimm-model scaling (G* ∼ *ω*^2/3^), which is generally what is observed in dilute or semidilute polymer solutions.^75^

**Fig. 9:**
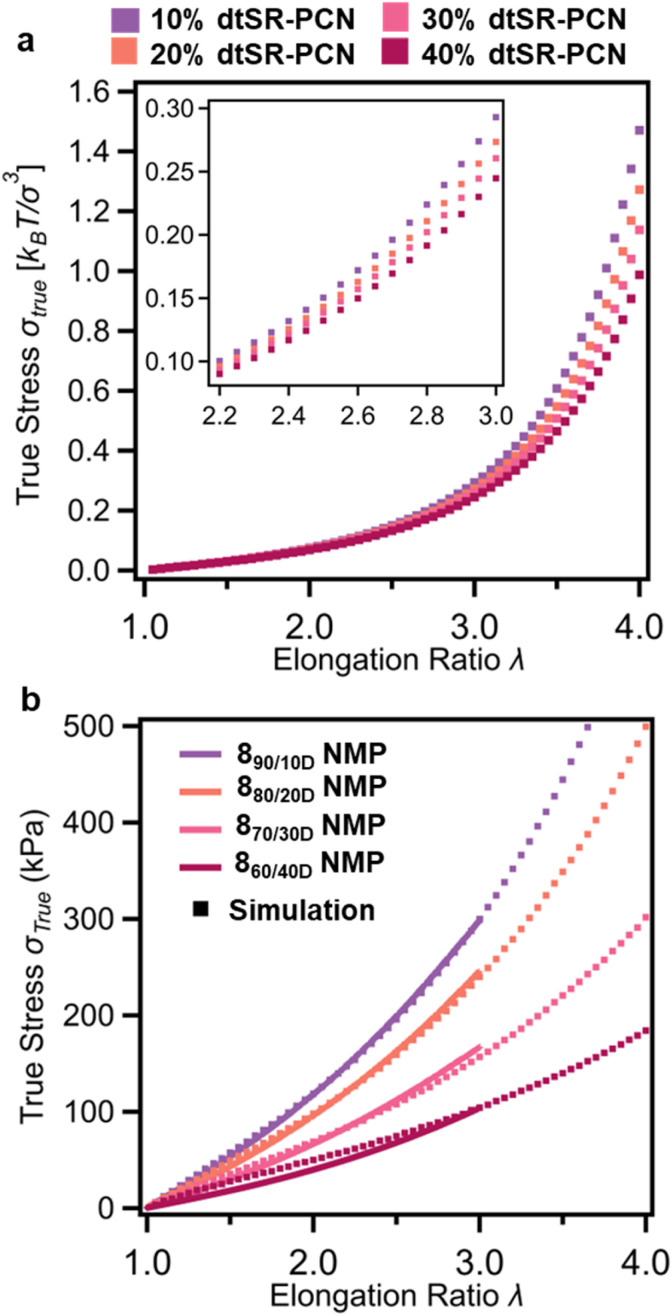
Tensile properties of the SR-PCNs 8_a/bD_ with different percentages of SR crosslinks and simulation studies on the SR-PCN swollen in NMP. (a) Dependence of the true stress *σ*_true_ on elongation ratio *λ* obtained in simulations of dtSR-PCN made by crosslinking precursor chains with the degree of polymerization of 1025 and *n*_*x*_ = 150. (b) True stress-elongation ratio curves of the SR-PCNs 8_a/bD_ by experimental tensile testing (solid lines; strain rate of 20% min^−1^ with *λ* limited to 3, before the material breaks) matched with compressive true stress-elongation curves from simulated modified SR-PCN made by crosslinking precursor chains with the degree of polymerization of 1025 and *n*_*x*_ = 150 with defects.

Tensile testing of 8_a/bD_, conducted through both simulations and experiments (Fig. 9a and b), indicates a decrease in true stress with elongation when introducing more rings (*e.g.*8_90/10D_ and 8_60/40D_ have a true stress of 298 kPa and 104 kPa, respectively, at an elongation ratio of 3). Moreover, Young's modulus (*E*, Fig. S90) from experiments shows a decrease (from *ca.* 94 kPa for 8_90/10D_ to *ca.* 34 kPa for 8_60/40D_) when the SR-PCNs contain more ring crosslinks. These observations which can be explained, at least in part, with increased swelling of the SR-PCNs with more ring crosslinks. Comparison of stress-elongation curves between simulations (dotted lines in Fig. 9b) and experiments (solid lines in Fig. 9b) provides deeper insights into how the addition of the P3R crosslinker into the network synthesis alters the network topology and subsequently the mechanical properties. Incorporating swelling effects into the simulations performed at a constant pressure, the stress-elongation behavior of the dtSR-PCN model with a 10% ring fraction closely matches experimental results (Fig. S91). However, discrepancies between experimental and simulated stress-elongation behaviors were observed for dtSR-PCNs with ring fractions ranging from 20% to 40%. To address these inconsistencies, the impact of network defects was explicitly considered. For example, reducing the functionality (*f*) of 12.5% of the total covalent crosslinks from 4 to 3 in the 20% dtSR-PCN model resulted in a match to the experimental data of 8_80/20D_ (Fig. S91). To achieve a comparable match with the experimental results of 8_70/30D_, the 30% dtSR-PCN model required conversion of 20% of the covalent crosslinks (*f* = 4) to *f* = 2 (Fig. S91). It should be noted that, in both cases, no covalent crosslinks adjacent to the SR crosslinks were altered. Both of these results suggest that the addition of the P3R crosslinker in the synthesis does increase the defects in the gels. For the 40% dtSR-PCN model to match with the experimental 8_60/40D_ true stress-elongation data, more significant addition of network defects was required. In addition to reducing 15% of the covalent crosslinks to *f* = 2, akin to what was done before, it was necessary to randomly remove 20% of the total covalent crosslinks in the network, as shown in Fig. S91 (see SI for details). It is important to note that while the experimental SR-PCNs may not have an identical topology as the simulated dtSR networks, these results do demonstrate that addition of more P3Rs in the synthesis results in increased network defects. This increase in defects can be explained, at least in part, by the observed increase in the formation of catenane byproducts when using more P3Rs in the synthesis (Fig. 3b and S92). No matter the cause of the defects it is important to note that the resulting topological changes will certainly contribute to the observed decrease in the network stiffness.

**Fig. 10:**
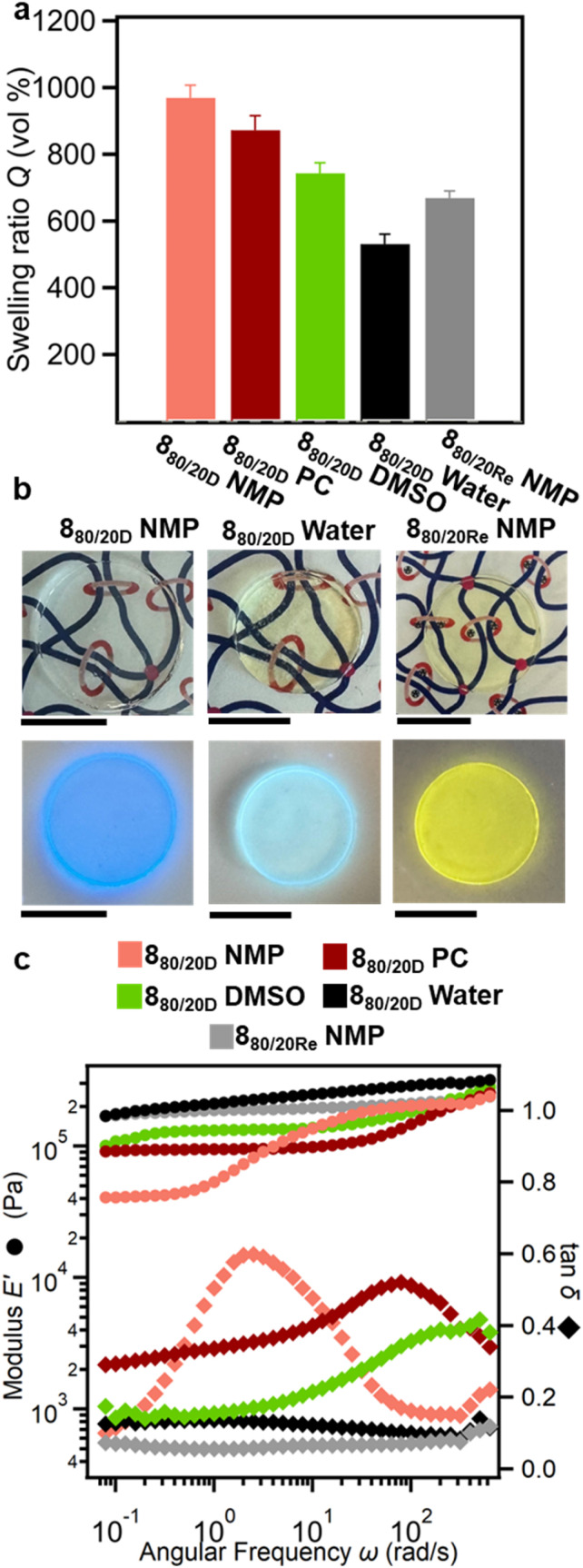
Swelling behavior and viscoelastic properties of SR-PCN swollen with different solvent and with metalation. (a) Swelling ratios of demetalated SR-PCN 8_80/20D_ in NMP, propylene carbonate (PC), dimethyl sulfoxide (DMSO), and water and the remetalated SR-PCN 8_80/20Re_ in NMP. (b) Optical images of 8_80/20D_ swollen in NMP and water and 8_80/20Re_ swollen in NMP under ambient light (top) and under 365 nm UV light (bottom). 1 cm scale bar (c) storage moduli *E*′ and loss factor tan *δ* of the demetalated SR-PCN 8_80/20D_ in different solvents and after remetalation.

To better explore the impact of solvent on these SR-PCNs, 8_80/20D_ was swollen in propylene carbonate (PC), dimethyl sulfoxide (DMSO), and water. The *Q* in these different solvents is shown in Fig. 10a, with 8_80/20D_ exhibiting the highest swelling in NMP (*ca.* 970%), followed by PC (*ca.* 870%) and DMSO (*ca.* 740%). 8_80/20D_ gels appear optically transparent in these solvents and possess a strong blue fluorescence under 365 nm UV light, consistent with the free BIP ligand (Fig. 10b and S93).^76^ SAOC of the organogels of 8_80/20D_ (Fig. 10c and S94) show that they all exhibit the sliding transition; however, the frequency of the tan *δ* peak varies depending on the solvent. As mentioned above in NMP, the peak in tan *δ* is observed at a frequency of 3 rad s^−1^. The peak moves to higher frequencies in PC (80 rad s^−1^) and DMSO (500 rad s^−1^) gels. This peak in tan *δ* tracks with the *Q* in the organogels, suggesting that this could be attributed to variations in the degree of polymer chain stretching in the gel networks. For example, the 8_80/20D_ gels with a smaller *Q* suggest that the polymer chains are less stretched, and the sliding of the ring occurs at higher frequencies on account of a shorter ring sliding path.

In water, 8_80/20D_ shows reduced swelling (*ca.* 530%) and exhibits a weakly fluorescent blue color under 365 nm UV light (Fig. 10b). The fluorescence spectrum of the 8_80/20D_ hydrogel shows a broader, lower-intensity emission peak relative to the peak in NMP. Furthermore, there is a bathochromic shift in the emission peak from 439 nm for 8_80/20D_ in NMP to 484 nm for the 8_80/20D_ hydrogel (Fig. S95). This observation is consistent with the stacking/aggregation observed for other fluorophores.^64,77–79^ Taken together, the swelling and fluorescence data support the aggregation of the BIP moieties in the hydrogels. Furthermore, the SAOC of the aqueous gel shows no sliding transition and a disappearance of the characteristic tan *δ* peak in the frequency sweep (Fig. 10c and S94) and its *E*′ storage modulus is similar to that of the covalent gel. Thus, while the PEG backbone is soluble in water, the BIP moieties used in the ring and backbone of the 8_80/20D_ are not, which hinders ring mobility and prevents sliding.

To further explore the impact of the ring mobility within the SR-PCN gels, the responsiveness of the gels to the addition of metal ions or acid was also examined. Given that these SR-PCNs contain ligands, an obvious method to inhibit ring mobility is to explore the remetalation of the gels. Upon adding enough Zn(NTf_2_)_2_ to the network to obtain 1 : 2 metal–ligand complexes, the 8_80/20Re_ NMP gel exhibits a pale-yellow color and fluoresces yellow under 365 nm UV light (Fig. 10b), both consistent with the formation of Zn(ii):BIP_2_ complexes.^76^ The remetalated 8_80/20Re_ exhibited reduced swelling in NMP (*ca*. 670% *vs.* 970% for 8_80/20D_), consistent with the presence of additional crosslinks. Furthermore, SAOC showed only one plateau in *E*′ and no peak in tan *δ* in the frequency sweep (Fig. 10c), akin to what is observed in the fully covalent networks. This is consistent with remetalation locking the rings in place.

**Fig. 11:**
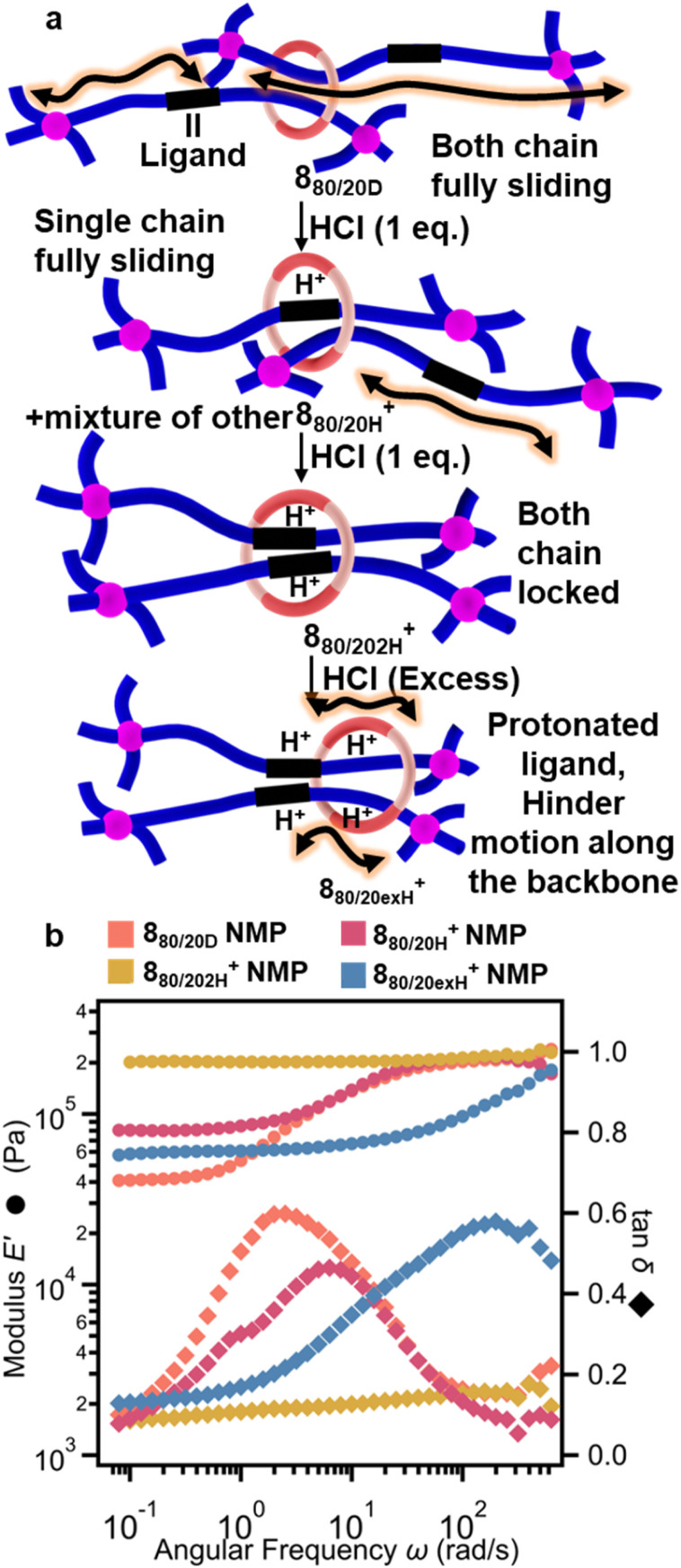
(a) Schematic showing the proposed impact of the addition of acid to the SR-PCN. The unprotonated 8_80/20D_ allows polymer/ring mobility. Partial protonation with one equivalent of acid (8_80/20H_^+^) binds some of the polymer chains and the rings. Addition of two equivalents of acid (8_80/202H_^+^) results in the formation of interlocked BIP_2_:H^+^ complexes that locks the ring and polymer (no sliding). Further protonation on the BIP moieties (8_80/20exH_^+^) results in reengaging the ring and polymer mobility on account of electrostatic repulsion. (b) Storage moduli *E*′ and tan *δ* of SR-PCN 8_80/20D_ in NMP with different amounts of added acid.

The BIP ligands in these SR-PCNs can also be protonated, so it was of interest to see if the behavior of these gels was sensitive to the addition of acid. In 1986, Sauvage reported that phenanthroline-based [2]catenanes exhibit enhanced basicity, and protonation results in the formation of interlocked phenanthroline_2_:H^+^ complexes that switch off the relative component motions,^80^ making them a class of pH-sensitive MIMs.^81,82^ As such, it was hypothesized that the presence of the interlocked, basic BIP moieties should result in acid-responsive SR-PCNs. To investigate this, SAOC was used to measure the viscoelastic properties of the SR-PCNs with varying amounts of acid (Fig. 11a and S96). Addition of one equivalent of hydrochloric acid with respect to the SR moiety (0.25 equivalent with respect to BIP units) in 8_80/20D_ yields 8_80/20H_^+^. A frequency sweep of this gel (Fig. 11b) shows a slight decrease in the intensity of the tan *δ* peak, which also shifts to slightly higher frequencies. This data is consistent with the protonation of some of the BIP moieties on the ring and thread, resulting in formation of a BIP_2_:H^+^ complex that inhibits sliding of the ring (Fig. 11a). The addition of two equivalents of protons with respect to the SR moieties yields the gel 8_80/202H_^+^ which not only exhibits an *E*′ similar to that of the covalent or metalated gels, but the frequency sweep data shows no transition in *E*′ and no peak in tan *δ*. This data suggests that the rings are no longer mobile and is consistent with the conversion of most of the BIP ligands to BIP_2_:H^+^ complexes. Addition of excess acid results in 8_80/20exH_^+^ exhibiting a reappearance of the transition in *E*′ and peak in tan *δ*, albeit at much higher frequencies than 8_80/20D_. This can be explained by excess protonation of the BIP moieties on both the polymer and rings, leading to electrostatic repulsion of the protonated BIP moieties re-engaging the mobility of the rings (Fig. 11a). The shift in tan *δ* towards higher frequency is consistent with a shorter ring sliding path length on account of electrostatic repulsions reducing ring movement along the polymer backbone.

## Conclusions

Through the careful design of the relevant components, it has been possible to increase the reaction kinetics of the metal-templated dtP3R crosslinker, allowing it to be more efficiently incorporated into the networks, resulting in SR-PCNs with a significant improvement in gel fraction and ring content. In addition, the use of extended thread components in the P3R and nitrile oxide chain extenders was shown to reduce the amount of catenane byproducts in the network. The improved synthesis of these SR-PCNs, combined with studies on covalent and tangled control networks, has allowed for a much-improved understanding of the impact of the doubly threaded SR crosslinks in these SR-PCNs. Swelling studies, compression rheology, tensile testing, dynamic light scattering microrheology, and molecular modeling were all carried out to compare the properties of the SR-PCNs with corresponding tangled and covalent networks. While the incorporation of the SRs into the gel results in a higher swelling ratio, the SRs also impart a distinct frequency-dependent mechanical behavior to the gels. At higher frequencies, the SR-PCNs have a compression storage modulus comparable to that of the covalent networks. However, when probed at lower frequencies, gels show a much lower modulus, consistent with movement of the chains through the ring over longer time scales. Supporting these findings, computational simulations revealed that purely covalent networks exhibit the highest stress response, followed by the tangled networks and then the dtSR-PCNs, reflecting the impact of crosslink flexibility and network topology. Further analysis of dtSR-PCN indicated that the introduction of higher amounts of P3R crosslinker in the synthesis introduces more network defects and topological changes, thereby further reducing the stiffness of the networks.

In addition, these SR-PCNs were shown to be stimuli-responsive. Changes in solvent, metalation, and protonation can be used to manipulate the ring sliding, which in turn changes their viscoelastic behavior, particularly the frequency dependence of the gels. Overall, these insights establish a framework for the rational design of dtSR-PCNs with tunable mechanical properties, highlighting the critical importance of having both flexible crosslinks and controlling defects in network structure. The findings of these concerted experimental and computational studies presented in this work have led to a better understanding of how the SR and polymer architecture impact the properties of this class of stimuli-responsive MIPs.

## Author contributions

G. L. and S. J. R. proposed the study. G. L. and J. O. conducted the synthesis, characterization, and analysis of all materials described with the assistance of J. E. H., B. W. R., N. N., and C. A. L. Simulations were conducted by Y. T., H. L., and J. J. d. P. The study was supervised by S. J. R. and J. J. d. P. The manuscript was written by G. L., J. O., Y. T., J. E. H., and S. J. R. The manuscript was written with the contributions from all authors. All authors have given approval to the final version of the manuscript.

## Conflicts of interest

There are no conflicts to declare.

## Supplementary Material

SC-016-D5SC05459A-s001

## Data Availability

The authors declare that all data supporting the findings of this study, including synthesis and characterization of monomers, supramolecular crosslinkers, and gels, are available within the article and SI. Supplementary information: synthetic procedures, NMR spectra, fluorescence spectroscopy, swelling and viscoelastic experiments, tensile testing, dynamic light scattering, molecular dynamics simulations, and additional supporting figures and references. See DOI: https://doi.org/10.1039/d5sc05459a.
