# Supplementary material for: Structure–property relationships of responsive doubly-threaded slide-ring polycatenane networks
Source: Chem Sci. 2025 Sep 17;16(41):19192–204. doi: 10.1039/d5sc05459a (PMC12442903; doi:10.1039/d5sc05459a)
Supplement: SC-016-D5SC05459A-s001 [file SC-016-D5SC05459A-s001.pdf]

Supplementary Information (SI) for Chemical Science.
This journal is © The Royal Society of Chemistry 2025

Electronic Supporting Information

Structure-Property Relationships of Responsive Doubly-Threaded Slide-Ring Polycatenane Networks

Guancen Liu,[a†] Jongwon Oh,[b†] Yuan Tian,[bc†] Jerald E. Hertzog,[b] Heyi Liang,[b] Benjamin W. Rawe,[b] Natsumi Nitta,[b] Charlie A. Lindberg,[b] Hojin Kim,[bd] Juan J. de Pablo,*[bcef] and Stuart J. Rowan*[abg]

[a]*Department of Chemistry, University of Chicago, Chicago, IL 60637, USA. E-mail: stuartrowan@uchicago.edu*

[b]*Pritzker School of Molecular Engineering, University of Chicago, Chicago, IL 60637, USA. E-mail: jjd8110@nyu.edu*

[c]*Department of Chemical and Biomolecular Engineering, Tandon School of Engineering, New York University, Brooklyn, NY 11201, USA.*

[d]*James Franck Institute, University of Chicago, Chicago, IL 60637, USA.*

[e]*Department of Computer Science, Courant Institute of Mathematical Sciences, New York University, New York, NY 10012, USA.*

[f]*Department of Physics, New York University, New York, NY 10003, USA.*

[g]*Chemical Science and Engineering Division and Center for Molecular Engineering, Argonne National Laboratory, 9700 S. Cass Ave., Lemont, IL 60434, USA.*

† These authors contributed equally to this work.

Table of Contents

Materials 4
Instrumentation 5
Synthesis of deprotected *N*-hexyl Bip **12** 6
Synthesis of bisbromide **13** 6
Synthesis of macrocycle **1** 7
Synthesis of deprotected *N*-ethyl Bip **15** 7
Synthesis of thread **2** 8
Zn(II) Assembly of Pseudo[3]rotaxane (P3R) **$1{:}2_2{:}Zn(II)_2$** 8
Prepolymerization for **4_{5k}** with **3a** 11
Prepolymerization for **$1{:}2_2{:}Zn(II)_2$** with **3a** 12
Catenane reaction for **$1{:}2_2{:}Zn(II)_2$** with **3a** 13
Synthesis of diphenyl nitroethene **22** 15
Synthesis of dinitro hexaethylene glycol **23** 16
Synthesis of monomer **3b** 16
Prepolymerization for **$1{:}2_2{:}Zn(II)_2$** with **3b** 17
Catenane reaction for **$1{:}2_2{:}Zn(II)_2$** with **3b** 19
Synthesis of propargyl triethylene glycol **16** 21
Synthesis of propargyl hexaethylene glycol **17** 22
Synthesis of propargyl nonaethylene glycol **18** 22
Synthesis of propargyl triethylene glycol tosylate **19** 22
Synthesis of propargyl hexaethylene glycol tosylate **20** 23
Synthesis of propargyl nonaethylene glycol tosylate **21** 23
Synthesis of thread **6a** 23
Synthesis of thread **6b** 24
Synthesis of thread **6c** 25
^{1}H NMR and ^{13}C NMR for intermediates and components 26
Zn(II) Assembly of Pseudo[3]rotaxane (P3R) **$1{:}6a_2{:}Zn(II)_2$** 33
Zn(II) Assembly of Pseudo[3]rotaxane (P3R) **$1{:}6b_2{:}Zn(II)_2$** 35
Zn(II) Assembly of Pseudo[3]rotaxane (P3R) **$1{:}6c_2{:}Zn(II)_2$** 37
DOSY NMR of the macrocycle, threads, and Zn(II) assemblies 39
Prepolymerization for **$1{:}6a_2{:}Zn(II)_2$** with **3b** 45
Prepolymerization for **$1{:}6b_2{:}Zn(II)_2$** with **3b** 47
Prepolymerization for **$1{:}6c_2{:}Zn(II)_2$** with **3b** 49
Catenane reaction for **$1{:}6c_2{:}Zn(II)_2$** with **3b** 51
Doubly-Threaded Slide-Ring Polycatenane Network (SR-PCN) $7_{80/20}$ and $8_{a/b}$ Synthesis 56
Demetalation and Washing 57
Gel Fraction (GF) for **$8_{a/bM}$** 58
NMR analysis on the soluble fractions 59

Zn(II) Assembly of **$6c_2$:Zn(II)** ... 63
Control Network $9_{a/b}$ Synthesis ... 66
Demetalation and Washing ... 67
Control Network $10_{a/b}$ Synthesis and Washing ... 68
Gel Fraction (GF) for **$9_{a/b}$** ... 69
Gel Fraction (GF) for **$10_{a/b}$** ... 70
Swelling procedure and swelling ratio (vol%) ... 70
Small-amplitude oscillatory compression (SAOC) in NMP ... 72
Storage and loss moduli from small-amplitude oscillatory compression (SAOC) in NMP ... 73
Tensile testing in NMP ... 76
Coarse-Grained Molecular Dynamics (MD) Simulations ... 77
Storage modulus from small-amplitude oscillatory compression (SAOC) for **$10_{a/b}$** in NMP ... 80
Dynamic light scattering (DLS) microrheology in NMP ... 81
Computational Interpretation of Experimental Tensile Testing ... 85
Pictures of organogel **$8_{80/20D}$** ... 87
Storage and loss moduli from small-amplitude oscillatory compression (SAOC) in different solvents and remetalation ... 88
Fluorescence spectrometry ... 89
Remetalation procedure ... 89
Acidification procedure ... 89
Storage and loss moduli from small-amplitude oscillatory compression (SAOC) after acidification in NMP ... 90
References ... 90

Table of equations

Equation	Page Number
Equation S1	57
Equation S2	57
Equation S3	59
Equation S4	61
Equation S5	69
Equation S6	70
Equation S7	77
Equation S8	78
Equation S9	79
Equation S10	79

Materials

All chemicals were purchased from Sigma-Aldrich and used without further purification unless otherwise mentioned. Benzophenone imine and 4-toluenesulfonyl chloride were purchased from Oakwood Chemical. Zinc di[bis(trifluoromethylsulfonyl)imide] was purchased from Strem Chemicals and stored in a nitrogen desiccator. Sodium hydride (90%, dry powder) was purchased from Sigma-Aldrich and stored in a nitrogen glovebox. Triethylene glycol and thymol standard for quantitative NMR *Trace*CERT® were purchased from Sigma-Aldrich and stored in a nitrogen desiccator. Hexaethylene glycol was purchased from Thermo Scientific Chemicals and stored in a nitrogen desiccator. 3,6,9,12,15,18,21,24-Octaoxahexacosane-1,26-diol was purchased from AA Blocks and stored in a nitrogen desiccator. 4-Chlorophenyl isocyanate was purchased from Sigma-Aldrich and stored in a refrigerator at 8°C before use. *N*-Bromosuccinimide (NBS) was purchased from Sigma-Aldrich and purified by recrystallization from 95 °C water. 2,2′-Azobis(2-methylpropionitrile) (AIBN) was purchased from Sigma-Aldrich and purified by recrystallization from 50 °C methanol. 4-arm PEG-Alkyne ($\mathbf{4}_{\mathbf{5k}}$, M_n = 5.0 kgmol^{-1}) and 4-arm PEG-Alkyne ($\mathbf{4}_{\mathbf{20k}}$, M_n = 20.0 kgmol^{-1}) were purchased from Creative PEGWorks and stored in a freezer at -37°C before use. TMS silica particles (500 nm) for DLS microrheology were purchased from CD Bioparticles and stored in a nitrogen desiccator. 2,6-bisbenzimidazolylpyridine ligands (**11** and **14**)[1] and nitrile-oxide monomer (**3a**)[2] were prepared following literature procedures. Solvents for chromatography were purchased from Fisher Scientific. Solvents for DLS and for curing of the networks ($\mathbf{8}_{\mathbf{a/bM}}$, $\mathbf{9}_{\mathbf{a/bM}}$, $\mathbf{10}_{\mathbf{a/b}}$) were HPLC grades from Fisher Scientific and filtered through a 0.2 µm membrane filter before use. Tetrahydrofuran (THF) was distilled over sodium and benzophenone. Dichloromethane (DCM) was distilled over calcium hydride. Dimethylformamide (DMF) was dried with activated 4Å molecular sieves (200 g of molecular sieves for 1 liter of DMF). Benzene was degassed by bubbling argon for 1 h. All synthesized components were stored in a freezer at -37°C before use. Thin layer chromatography (TLC) was performed on SiO2-60 F254 glass plates with visualization by UV light or staining. Column chromatography was performed using silica gel technical grade, pore size 60 Å, 230–400 mesh particle size, 40–63 µm particle size from Sigma-Aldrich.

Instrumentation

Room Temperature Nuclear Magnetic Resonance Spectroscopy (NMR) was performed using a 500 MHz Bruker Avance-II+ spectrometer equipped with a 1H{19F,13C,31P} QNP probe, using Topspin 3.6.2; a 500 MHz Bruker Avance-III spectrometer equipped with a BBO SmartProbe, using Topspin 3.6.2; a 500 MHz Bruker Avance-III spectrometer equipped with a BBO Prodigy Probe, using Topspin 3.6.2; and a 600 MHz Bruker Avance Neo spectrometer equipped with a BBO Prodigy Probe, using Topspin 3.6.2 at the University of Chicago Chemistry Department's NMR Facility. ^{1}H NMR spectra were referenced to the residual protonated solvent signal for single solvent solutions and the TMS signal for 5% acetonitrile-d_3 in chloroform-*d*. ^{13}C NMR spectra were referenced to the deuterated solvent carbon resonance signal.

Diffusion-Ordered NMR Spectroscopy (DOSY NMR) was obtained on dilute solutions in 5mm NMR tubes at a constant temperature of 25°C using a 500 MHz Bruker Avance-II+ spectrometer equipped with a 1H{19F,13C,31P} QNP probe, using Topspin 3.6.2. Diffusion measurements were obtained using the 2D Bruker pulse program dstebpgp3s, which includes a double stimulated echo for convection compensation, a longitudinal echo delay, bipolar gradient pulses, and three spoil gradients. The corresponding 1D pulse sequence dstebpgp3d1d was used to optimize the parameters D20 ("big delta", the major diffusion delay) and P30 ("little delta", the diffusion gradient length), in accord with manufacturer-recommended methods. The 2D data were acquired with a linear array of 32 diffusion gradient strengths (GPZ6 values) from 5% to 95%.[3–6]

NMR Titration was performed using a 500 MHz Bruker Avance-III spectrometer equipped with a BBO ProdigyProbe, using Topspin 3.6.2. Experiments were performed in a 5mm NMR tube fused with a 4mL screw cap vial on the top to provide extra volumes.

Matrix Assisted Laser Desorption/Ionization Mass Spectrometry (MALDI-TOF MS) was performed on a Bruker autoflex maX MALDI-TOF/TOF spectrometer at the University of Chicago Mass Spectrometry Facility in linear mode using dithranol as matrix and sodium trifluoroacetate as ionizer (or no ionizer).

Tensile testing was performed on a Zwick Roell – zwickiLine Z0.5 materials testing machine with a 200 N load cell at room temperature under ambient conditions. Network samples (Swollen in NMP) were cut using a razor blade. Gels were appropriately measured and loaded between metal tensile grips, with minimal handling without tweezers. Tensile testing measurements were performed at 5 mm strain min^{-1} until failure or 20% strain min^{-1} until 200% strain. Young's modulus was determined by linear fitting of the stress-strain curves below 1% strain.

Small Amplitude Oscillatory Compression (SAOC) Rheology was performed using a TA Instruments RSA GS2 with the 25 mm compression plate geometry. Network samples (swollen in NMP) were cut using a die punch and preloaded with 0.25 N to ensure uniform contact between the sample and plates. Frequency sweeps were performed from 1000 rad/s to 0.05 rad/s with a strain amplitude of 1%.

Fluorescence Spectrometry was measured using a Duetta 3-in-1 spectrofluorometer (HORIBA Scientific). The hydrogel was cut into a rectangular shape to adhere in a quartz 2mm x 10 mm cuvette from Hellma. After measuring the hydrogel, the hydrogel was fully dried inside the cuvette, and the same network in the cuvette was fully swollen in NMP and measured again.

Dynamic light scattering (DLS) microrheology was performed using a Malvern Zetasizer Nano ZS, and cuvettes (Quarzglas High Performance) for DLS were purchased from Hellma. Network samples (swollen in NMP) were placed in the cuvette with NMP that was freshly filtered through a 0.2 μm membrane filter before use. TMS silica particles were added in the second step of gelation (1.7 mg in 100 μL of 30% acetonitrile in chloroform). The experiments were conducted following literature methods, and the data was analyzed with the Github script provided in the literature.[7]

Synthesis of deprotected *N*-hexyl Bip **12**

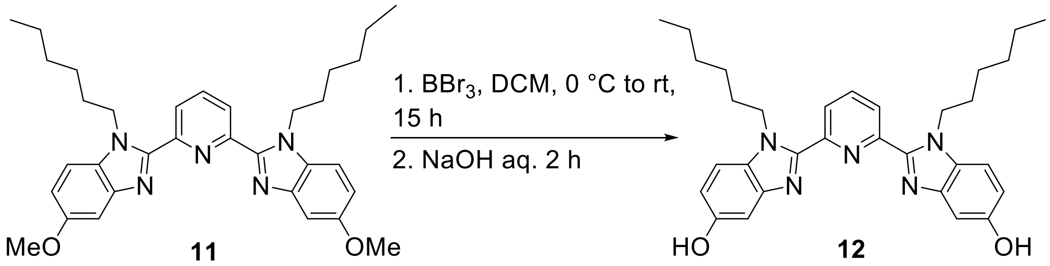

Figure S1. Synthesis of deprotected *N*-hexyl Bip **12**.

Protected *N*-hexyl Bip (2,6-bis(1-hexyl-5-methoxy-1*H*-benzo[*d*]imidazol-2-yl)pyridine) **11** (15.1 g, 28.0 mmol) was placed in a flame-dried two-neck round-bottom flask under argon atmosphere and dissolved in DCM (250 mL). The solution was cooled to 0°C in an ice-water bath, and boron tribromide (17.7 mL, 184 mmol) was added dropwise to the reaction mixture resulting in a deep red colored mixture. The reaction mixture warmed to room temperature and was stirred for 15 h. The solution was cooled to 0°C in an ice-water bath and slowly poured into a 4 L beaker containing 1 M aq. NaOH solution (1.5 L) in an ice-water bath. The mixture warmed to room temperature and was stirred for 2 h. Then the mixture was neutralized with 2M aq. HCl solution. The precipitate was collected by filtration to yield deprotected *N*-hexyl Bip (2,2'-(pyridine-2,6-diyl)bis(1-hexyl-1*H*-benzo[*d*]imidazol-5-ol)) **12** as a white solid (13.5 g, 94% isolated yield). The ^{1}H NMR and ^{13}C NMR data was consistent with the previously reported compound.[8]

Synthesis of bisbromide **13**

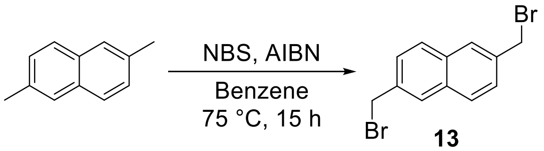

Figure S2. Synthesis of bisbromide **13**.

2,6-Dimethylnaphthalene (4.60 g, 29.4 mmol), N-bromosuccinimide (10.5 g, 59.0 mmol), and AIBN (33.8 mg, 0.206 mmol) were placed in a two-neck round-bottom flask with a reflux condenser under argon atmosphere. Degassed benzene (300 mL) was added via cannula. The solution was irradiated by UV lamp (4 W) and heated to 75°C for 15 h. After 15 h, the solution was cooled to 8°C in a refrigerator. The resulting succinimide was removed by filtration. The solution was then diluted with DCM (200 mL) and washed with saturated aq. sodium thiosulfate solution (3 × 300 mL). The organic layer was separated, dried with sodium sulfate, filtered, and the solvent was removed in vacuo. The solid was purified by successive recrystallizations from a mixture of hot chloroform/hexanes to yield bisbromide **13** as a white solid (2.80 g, 30% isolated yield). The ^{1}H NMR data was consistent with the previously reported compound.[9]

Synthesis of macrocycle **1**

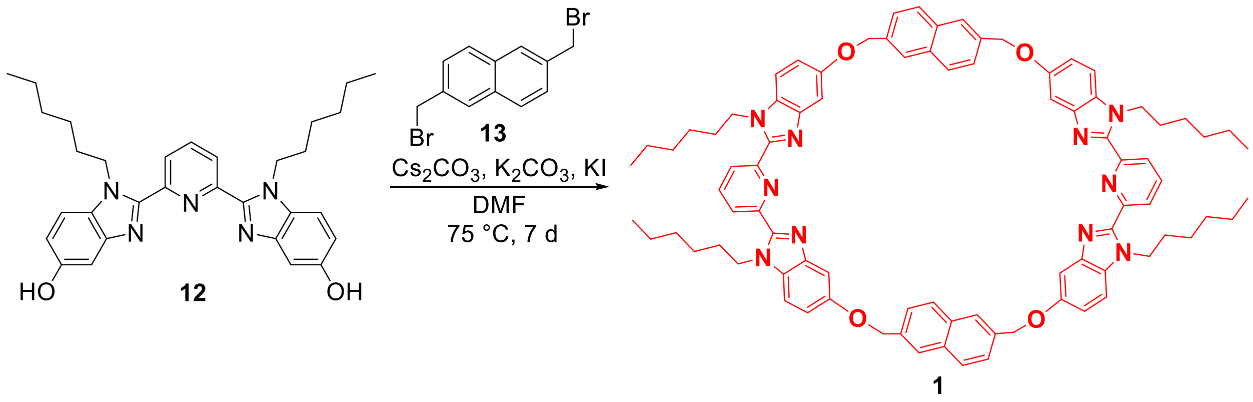

Figure S3. Synthesis of macrocycle **1**.

Cesium carbonate (17.6 g, 54.0 mmol), potassium carbonate (7.46 g, 54.0 mmol), and potassium iodide (0.26 g, 1.57 mmol) were placed in a two-neck round-bottom flask under argon atmosphere. DMF (900 mL) was added via cannula. The mixture was heated to 75 °C. To this mixture was added dropwise a mixture of deprotected *N*-hexyl Bip **12** (2.76 g, 5.40 mmol) and bisbromide **13** (1.69 g, 5.40 mmol) in DMF (900 mL) over a period of 2 d, and the reaction was stirred at 75 °C for another 5 d. The solvent was removed in vacuo. The residue was dissolved in chloroform, and the residual salt was removed by filtration. The solvent was removed in vacuo, and the solid was purified by triethylamine treated silica gel chromatography (0-1% methanol in chloroform) followed by recrystallization from a mixture of hot chloroform/methanol to yield macrocycle **1** as a white solid (135 mg, 4% isolated yield). The ^{1}H NMR and ^{13}C NMR data was consistent with the previously reported compound.[8]

Synthesis of deprotected *N*-ethyl Bip **15**

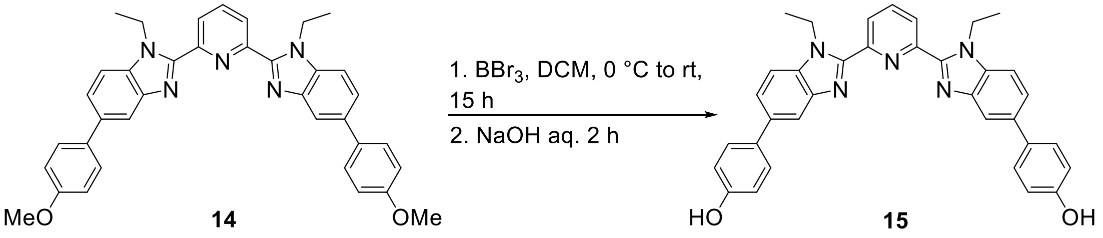

Figure S4. Synthesis of deprotected *N*-ethyl Bip **15**.

Protected *N*-ethyl Bip (2,6-bis(1-ethyl-5-(4-methoxyphenyl)-1*H*-benzo[*d*]imidazol-2-yl)pyridine) **14** (15.2 g, 26.2 mmol) was placed in a flame-dried two-neck round-bottom flask under argon atmosphere and dissolved in DCM (250 mL). The solution was cooled to 0°C in an ice-water bath, and boron tribromide (16.5 mL, 171 mmol) was added dropwise to the reaction mixture, resulting in a deep red colored mixture. The reaction mixture warmed to room temperature and was stirred for 15 h. The solution was cooled to 0°C in an ice-water bath and slowly poured into a 4 L beaker containing 1 M aq. NaOH solution (1.5 L) in an ice-water bath. The mixture warmed to room temperature and was stirred for 2 h. Then the mixture was neutralized with 2M aq. HCl solution. The precipitate was collected by filtration to yield deprotected *N*-ethyl Bip (4,4'-(pyridine-2,6-diylbis(1-ethyl-1*H*-benzo[*d*]imidazole-2,5-diyl))diphenol) **15** as a white solid (14.2 g, 98% isolated yield). The ^{1}H NMR and ^{13}C NMR data was consistent with the previously reported compound.[8]

Synthesis of thread **2**

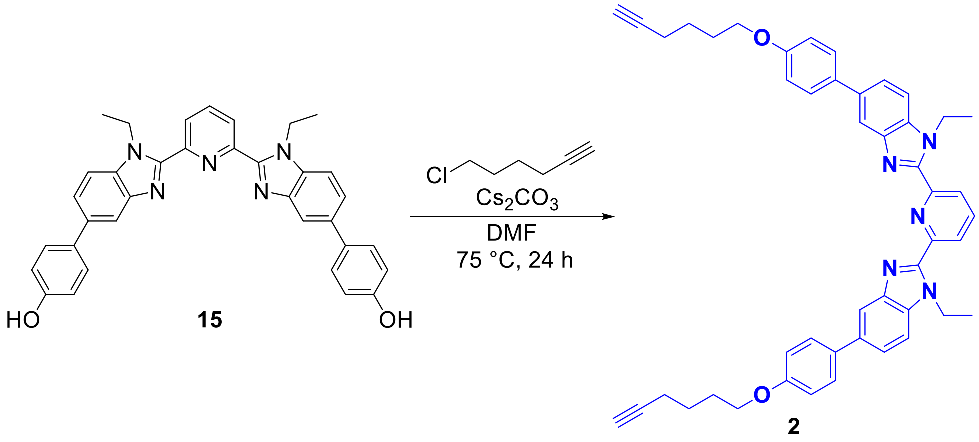

Figure S5. Synthesis of thread **2**.

Deprotected *N*-ethyl Bip **15** (4.00 g, 7.25 mmol) and cesium carbonate (9.45 g, 29.0 mmol) were placed in a flame-dried two-neck round-bottom flask under argon atmosphere, and DMF (72 mL) was added. 6-chloro-1-hexyne (3.50 mL, 29.0 mmol) was added. The mixture was heated to 75 °C for 24 h. The solvent was removed in vacuo. The residue was dissolved in chloroform, and the residual salt was removed by filtration. The solvent was removed in vacuo, and the solid was purified by silica gel chromatography (0-2% methanol in chloroform) to yield thread **2** as a white solid (4.30 g, 83% isolated yield). The ^{1}H NMR and ^{13}C NMR data was consistent with the previously reported compound.[10]

Zn(II) Assembly of Pseudo[3]rotaxane (P3R) **1:2₂:Zn(II)₂**

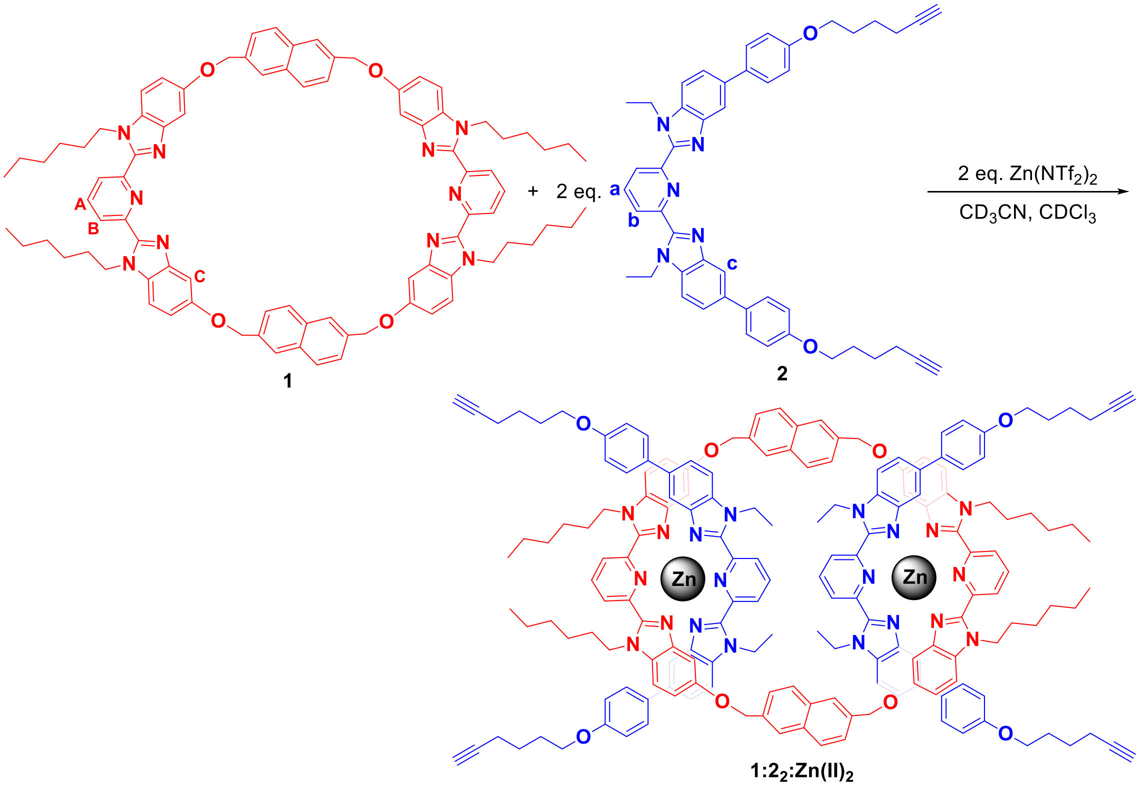

Figure S6. Formation of **$1:2_2:Zn(II)_2$**.

Macrocycle **1** (10.0 mg, 0.00753 mmol) was dissolved in chloroform-*d* (0.5 mL) and added to an NMR tube. A stock solution of thread **2** (12.9 mg, 0.0181 mmol) in chloroform-*d* (0.6 mL) was titrated into the NMR tube until an exact 1:2 (Macrocycle **1**:thread **2**) ratio was formed by monitoring both the N-CH_2 peaks (triplet at 4.44 ppm for macrocycle **1** and quartet at 4.83 ppm for thread **2**) on the alkyl size chain groups of the Bip ligands. Zinc di[bis(trifluoromethylsulfonyl)imide] (11.3 mg, 0.0181 mmol) in acetonitrile-d_3 (0.6 mL) was added until no free (unbound) Bip peaks appeared at ~2 equivalents of zinc di[bis(trifluoromethylsulfonyl)imide]. The complete disappearance of the doublets at 8.09 and 8.37 ppm indicates that all Bip ligands are bound with Zn^{2+} ions in a 2:1 Bip:metal ratio. The resulting pseudo[3]rotaxane **1:2₂:Zn(II)₂** solution was dried in vacuo to obtain a yellow solid that was redissolved in 1 mL 5% acetonitrile-d_3 in chloroform-*d*, and stirred at 45°C for 1 d to allow equilibration. The solvent was then removed in vacuo, resulting in a yellow solid that was stored in the freezer at -37°C before use.

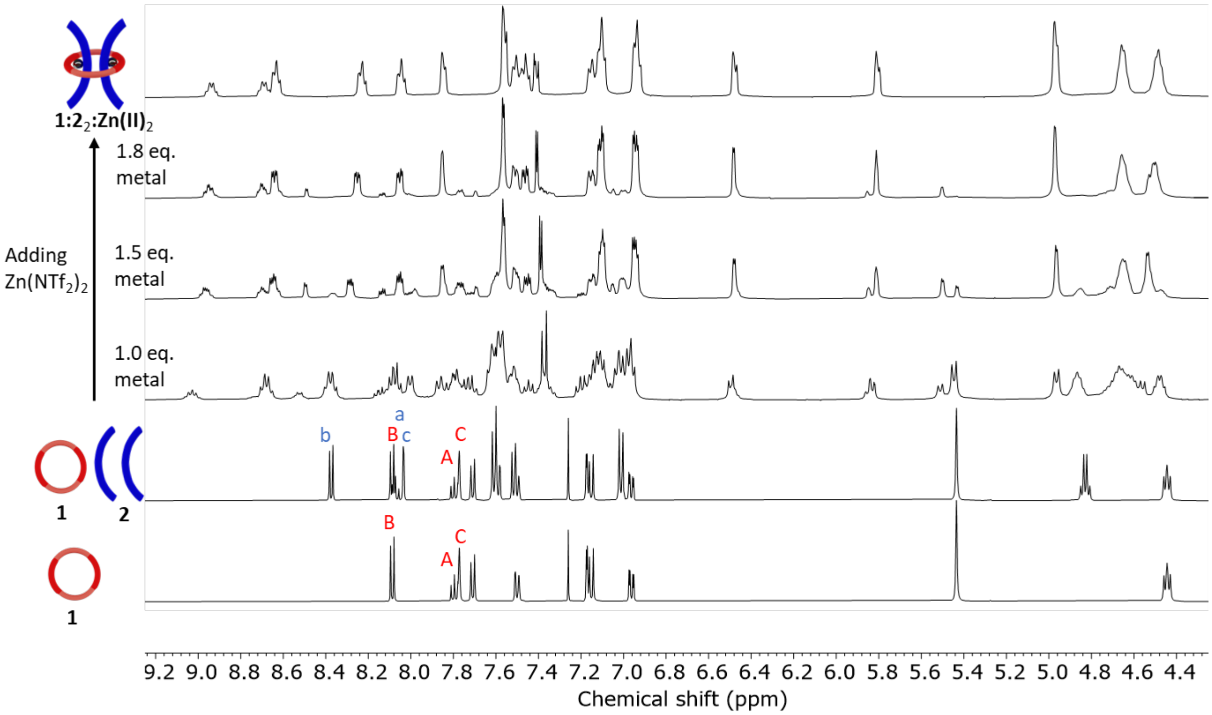

Figure S7. Partial ^{1}H-NMR overlay (500 MHz, 25°C, increasing acetonitrile-d_3 in chloroform-*d* increasing upwards) of metal addition during NMR titrations.

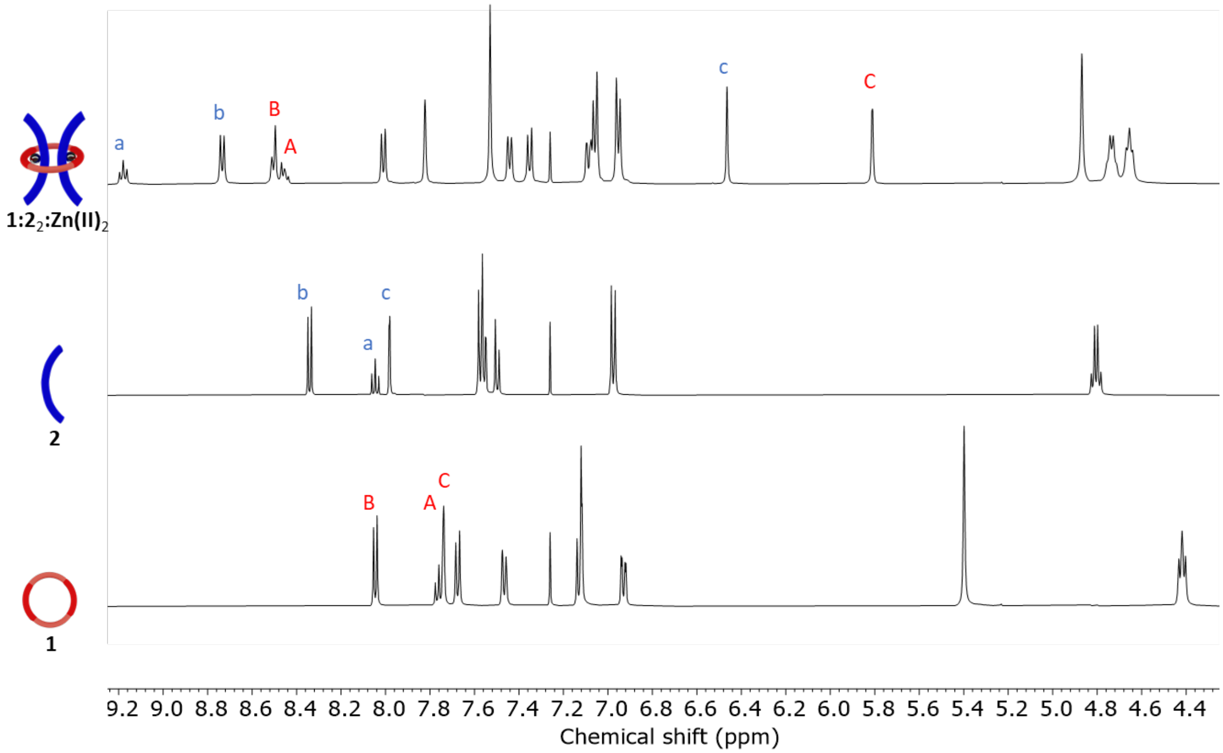

Figure S8. Partial ^{1}H-NMR overlay (500 MHz, 25°C) of 10 mM **1:2$_2$:Zn(II)$_2$**, 20 mM **2,** and 10 mM **1** (5% acetonitrile-d_3 in chloroform-d) after equilibration.

Prepolymerization for 4_{5k} with **3a**

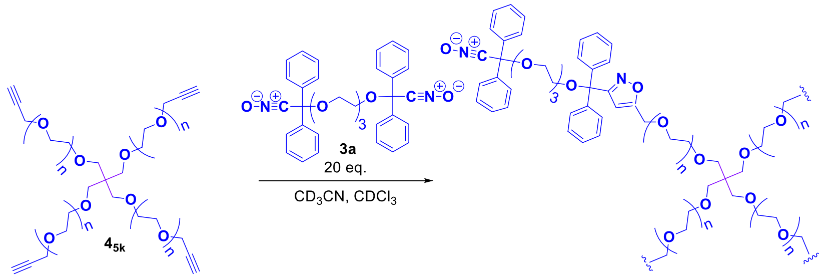

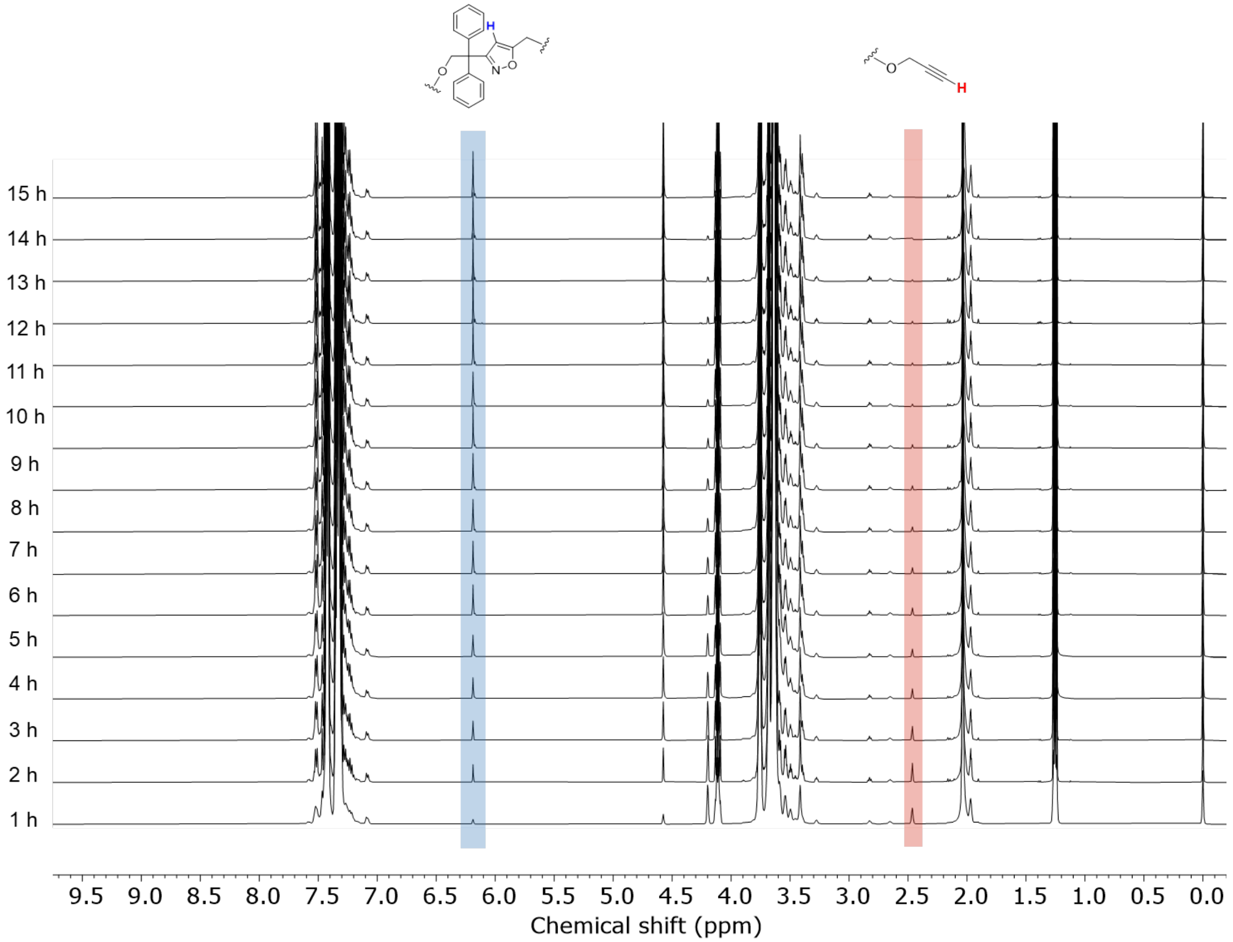

Figure S9. Prepolymerization of $\mathbf{4_{5k}}$ with an excess amount of monomer **3a** (20 equivalents) in 5% acetonitrile-d_3 in chloroform-*d* to determine the reaction kinetics. ^{1}H-NMR overlay (500 MHz, 25°C) of prepolymerization of $\mathbf{4_{5k}}$ with an excess amount of monomer **3a** (20 equivalents) at different reaction times (1 h to 15 h). The disappearance of the signal that corresponds to the terminal protons of the alkyne (2.46 ppm) and the increase of the signal that corresponds to the protons of the isoxazole (6.19 ppm) show that $\mathbf{4_{5k}}$ has been fully reacted.

Monomer **3a** (20.3 mg, 0.0359 mmol) and 4-arm PEG-alkyne $\mathbf{4_{5k}}$ (8.9 mg, 0.00177 mmol) were dissolved in 5% acetonitrile-d_3 in chloroform-*d* (0.5 mL) and added to an NMR tube. The NMR tube was constantly shaken by hand to allow thorough mixing.

Prepolymerization for **1:2$_2$:Zn(II)$_2$** with **3a**

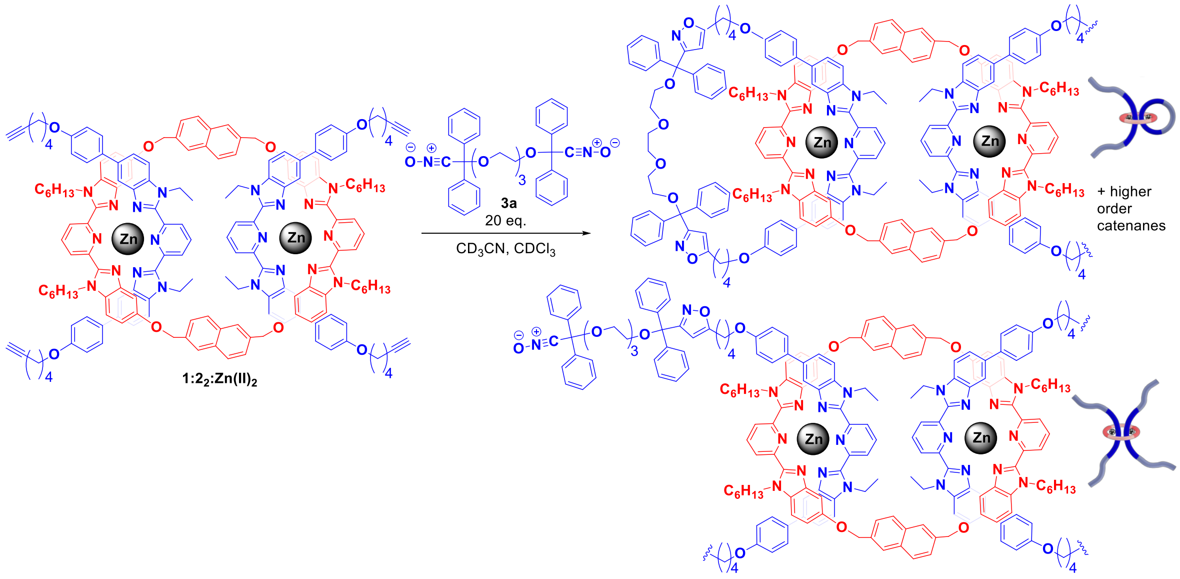

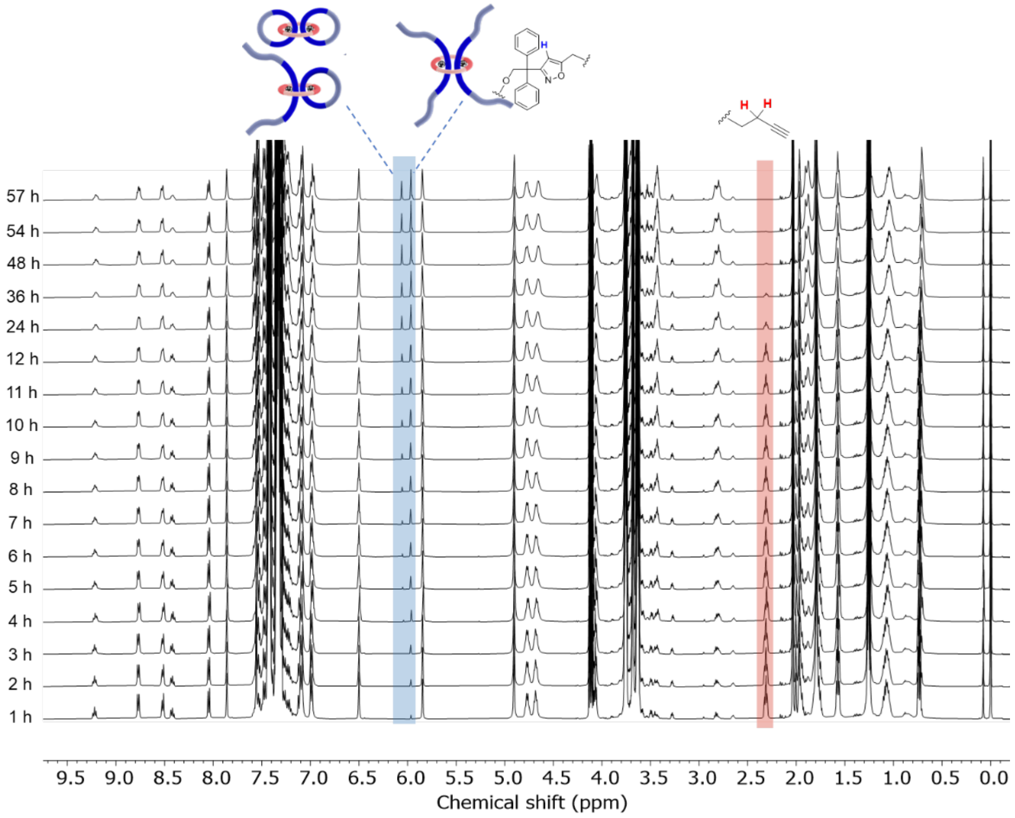

Figure S10. Prepolymerization of **1:2$_2$:Zn(II)$_2$** with an excess amount of monomer **3a** (20 equivalents) in 5% acetonitrile-*d*$_3$ in chloroform-*d* to determine the reaction kinetics and the amount of backbiting. Partial ^{1}H-NMR overlay (500 MHz, 25°C) of prepolymerization of **1:2$_2$:Zn(II)$_2$** with an excess amount of monomer **3a** (20 equivalents) at different reaction times (1 h to 57 h). The disappearance of the signal that corresponds to the α protons of the alkyne (2.30 ppm) shows that **1:2$_2$:Zn(II)$_2$** has been fully reacted. The increase of the signal that corresponds to the protons of the isoxazole (6.06 ppm for catenane, and 5.96 ppm for linear) shows the 35% backbiting.

Monomer **3a** (20.3 mg, 0.0359 mmol) and **1:2$_2$:Zn(II)$_2$** (7.1 mg, 0.00177 mmol) were dissolved in 5% acetonitrile-*d*$_3$ in chloroform-*d* (0.5 mL) and added to an NMR tube. The NMR tube was constantly shaken by hand to allow thorough mixing.

<u>Catenane reaction for **1:2$_2$:Zn(II)$_2$** with **3a**</u>

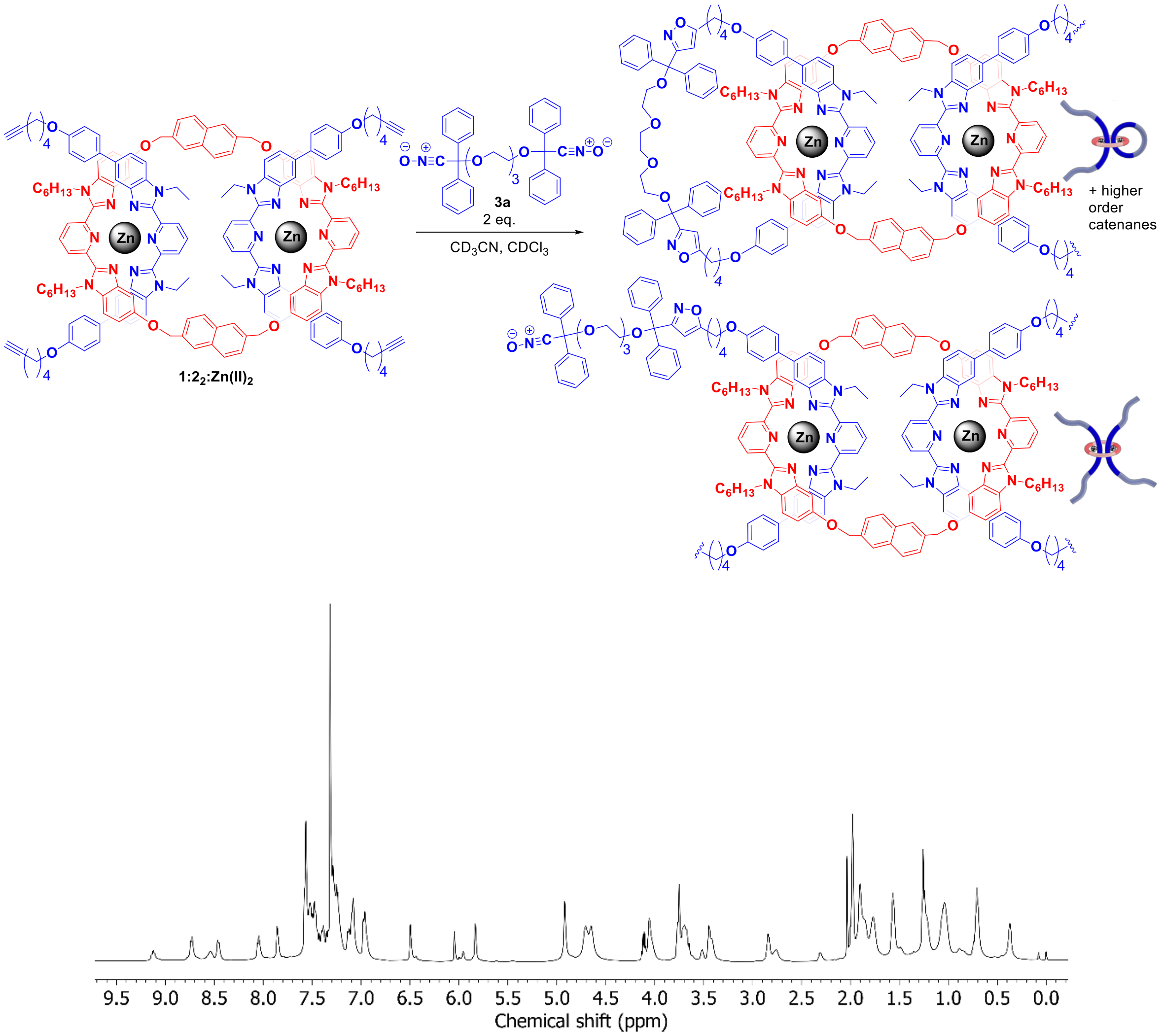

Figure S11. Cyclization of **1:2$_2$:Zn(II)$_2$** with two equivalents of monomer **3a** in 5% acetonitrile-*d*$_3$ in chloroform-*d* to target the catenane. Crude ^{1}H-NMR (500 MHz, 25°C) of cyclization of **1:2$_2$:Zn(II)$_2$** with two equivalents of monomer **3a** in 5% acetonitrile-*d*$_3$ in chloroform-*d* shows an increase in the protons of the isoxazole in the catenane and a decrease in the rotaxane.

Monomer **3a** (3.24 mg, 0.00574 mmol) and **1:2$_2$:Zn(II)$_2$** (11.5 mg, 0.00287 mmol) were dissolved in 5% acetonitrile-*d*$_3$ in chloroform-*d* (0.57 mL) and added to an NMR tube. The mixture was heated to 45 °C for 8 d. The crude NMR was taken. The solvent was removed in vacuo. The residue was dissolved in DCM (10 mL), and acetonitrile (0.5 mL) was added. To demetalate, tetrabutylammonium hydroxide solution (1M in methanol, 80 µL) was added dropwise to demetalate for 30 min. The reaction mixture was then diluted with DCM (5 mL) and washed with water (5 × 20 mL). The organic layer was separated, and the solvent was removed in vacuo.

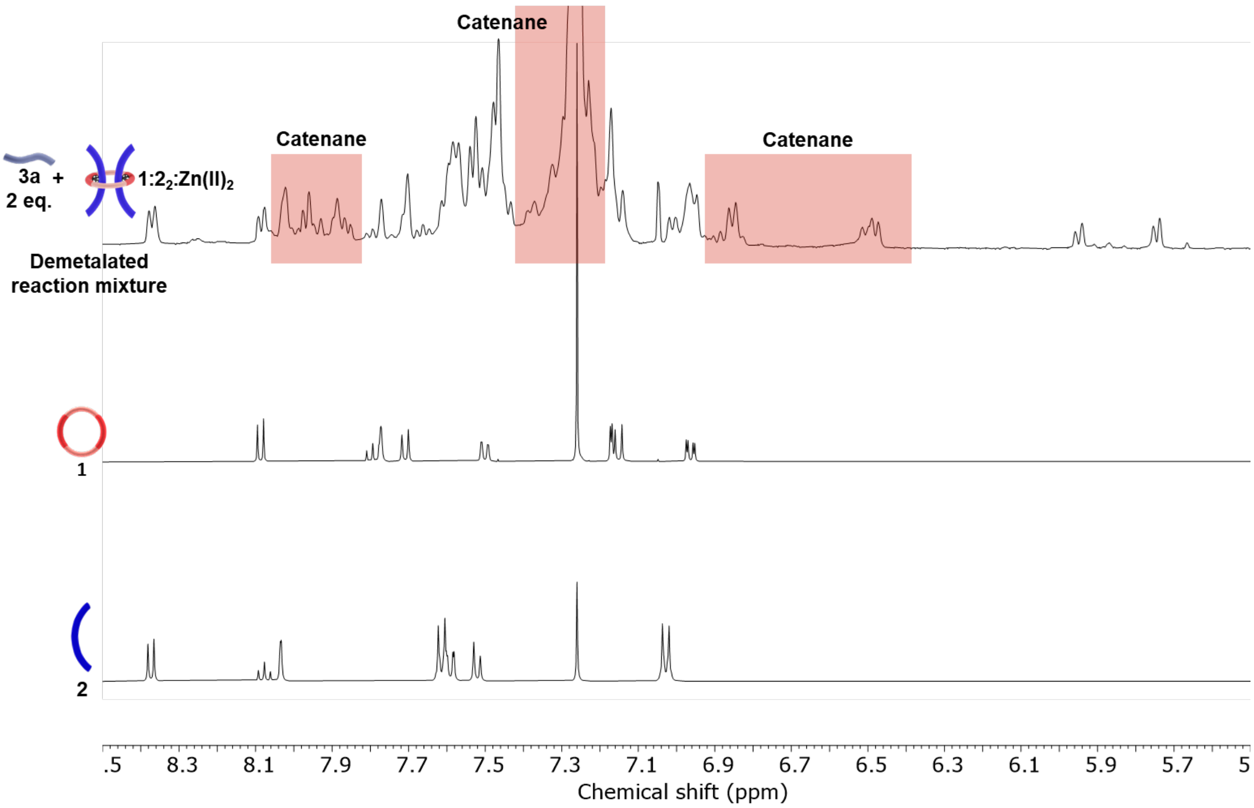

Figure S12. Partial ^{1}H-NMR overlay (500 MHz, $CDCl_3$, 25°C) of **1**, **2**, and the reaction mixture from Figure S11 after demetalation.

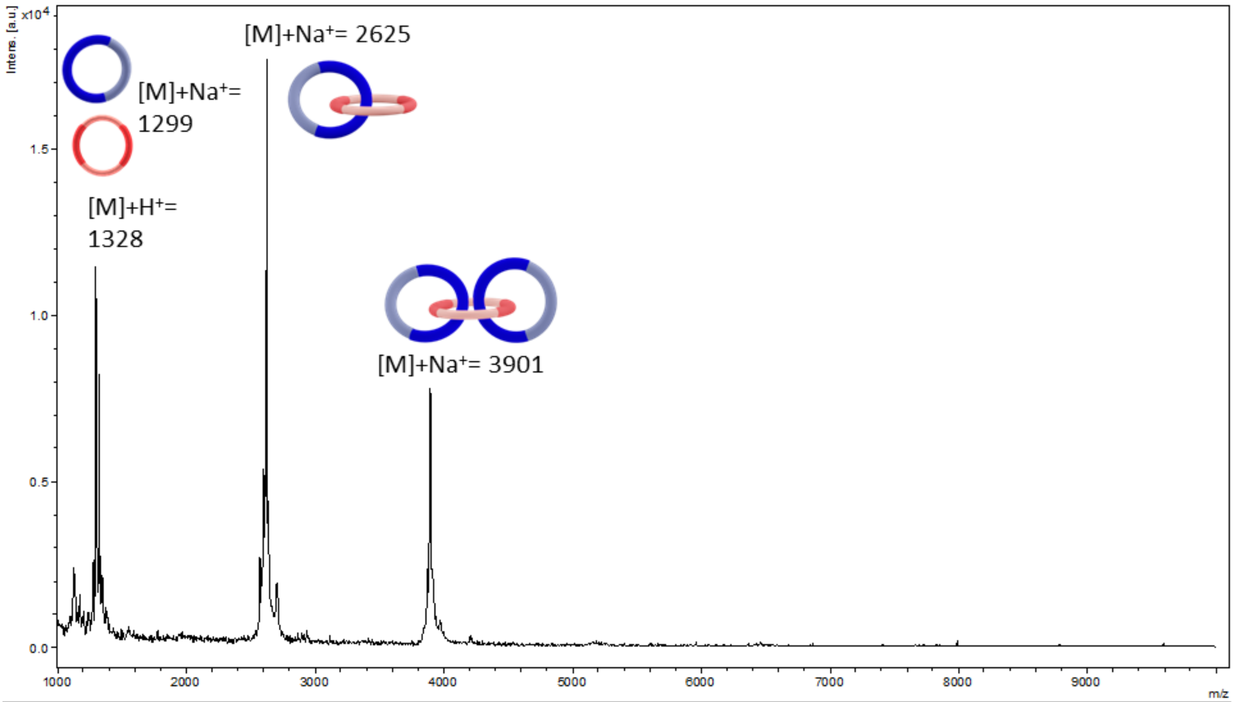

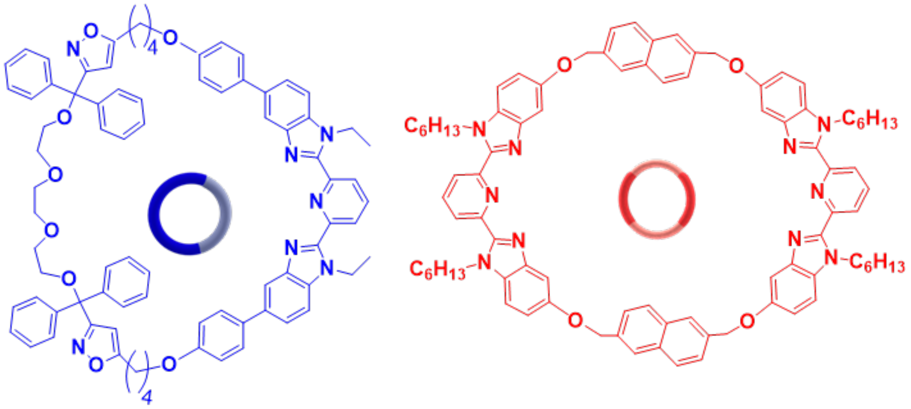

Figure S13. MALDI-TOF mass spectrometry data for the reaction mixture from Figure S11 after demetallation.

Synthesis of diphenyl nitroethene **22**

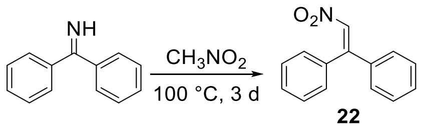

Figure S14. Synthesis of diphenyl nitroethene **22.**

Benzophenone imine (27 mL, 161 mmol) was placed in a flame-dried two-neck round-bottom flask under argon atmosphere and dissolved in nitromethane (27 mL). The mixture was heated to 100 °C for 3 d. The solvent was removed in vacuo. The residue was purified by recrystallization from a mixture of hot ethyl acetate/hexanes to yield diphenyl nitroethene **22** as yellow needles (22.7 g, 63% isolated yield) that was stored in the dark in a nitrogen glovebox. The ^{1}H NMR and ^{13}C NMR data was consistent with the previously reported compound.[11]

Synthesis of dinitro hexaethylene glycol **23**

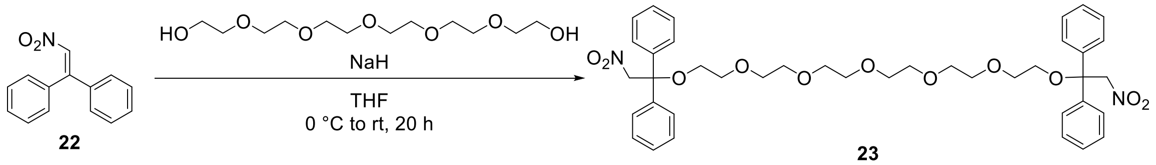

Figure S15. Synthesis of dinitro hexaethylene glycol **23.**

Sodium hydride (90% dry powder, 2.23 g, 83.6 mmol) was placed in a flame-dried three-neck round-bottom flask under argon atmosphere, and THF (9 mL) was added. The mixture was cooled to 0°C in an ice-water bath, and hexaethylene glycol (4.86 g, 17.2 mmol) in THF (13 mL) was added dropwise to the reaction mixture. The reaction mixture was stirred for 1 h. Then the ice-water bath was removed. The reaction mixture warmed to room temperature and was stirred for another 1 h. Diphenyl nitroethene **22** (15.5 g, 68.8 mmol) in THF (24 mL) was added dropwise to the reaction mixture. The reaction mixture was stirred for 18 h. The solution was cooled to 0°C in an ice-water bath, and acetic acid (40 mL) and water (40 mL) were added dropwise to the reaction mixture. The reaction mixture was then diluted with DCM (200 mL) and washed with 1 M aq. HCl solution (3 × 200 mL), water (1 × 200 mL), and brine (3 × 200 mL). The organic layer was separated, dried with sodium sulfate, filtered, and the solvent was removed in vacuo. The oil was purified by silica gel chromatography (10-70% ethyl acetate in hexanes) to yield dinitro hexaethylene glycol **23** as a yellow oil (10.1 g, 80% isolated yield). ^{1}H NMR (500 MHz, $CDCl_3$) δ 7.33 – 7.27 (m, 10H), 7.26 – 7.23 (m, 10H), 5.31 (s, 4H), 3.68 (t, *J* = 5.4 Hz, 4H), 3.64 – 3.58 (m, 16H), 3.49 (t, *J* = 5.4 Hz, 4H). $^{13}C\{^{1}H\}$ NMR (126 MHz, $CDCl_3$) 141.23, 128.58, 128.27, 126.93, 81.83, 80.29, 70.85, 70.78, 70.76, 70.73, 70.18, 63.04. MALDI-TOF MS: ([M]+Na^+) calc'd for $C_{44}H_{48}N_2NaO_{11}$: 755.316 found: 755.971.

Synthesis of monomer **3b**

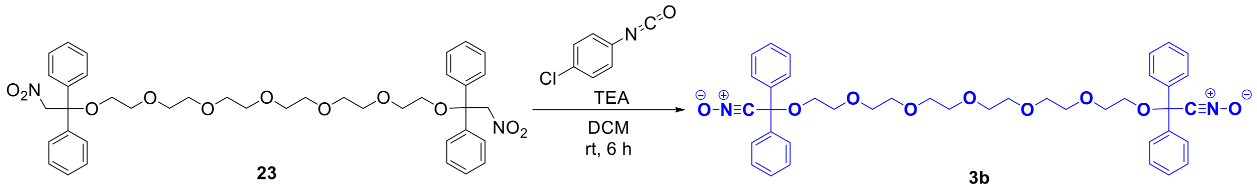

Figure S16. Synthesis of monomer **3b.**

Dinitro hexaethylene glycol **23** (5.20 g, 7.10 mmol) and 4-chlorophenyl isocyanate (4.36 g, 28.4 mmol) were placed in a flame-dried three-neck round-bottom flask under argon atmosphere and dissolved in DCM (24 mL). Triethylamine (5.90 mL, 42.3 mmol) was added dropwise to the reaction mixture. The reaction mixture was stirred for 6 h. The residual salt was removed by filtration. The solvent was removed in vacuo, and the oil was purified by silica gel chromatography (10-60% ethyl acetate in hexanes) to yield monomer **3b** as a yellow oil (3.45 g, 70% isolated yield) that was stored in the freezer at -37°C before use. ^{1}H NMR (500 MHz, $CDCl_3$) δ 7.46 – 7.39 (m, 8H), 7.39 – 7.29 (m, 12H), 3.76 – 3.70 (m, 4H), 3.67 – 3.60 (m, 20H). $^{13}C\{^{1}H\}$ NMR (126 MHz, $CDCl_3$) 140.54, 129.07, 128.85, 126.58, 84.36, 70.92, 70.80, 70.74, 69.95, 66.18. MALDI-TOF MS: ([M]+Na^+) calc'd for $C_{40}H_{44}N_2NaO_9$: 719.295 found: 719.348.

Prepolymerization for **1:2$_2$:Zn(II)$_2$** with **3b**

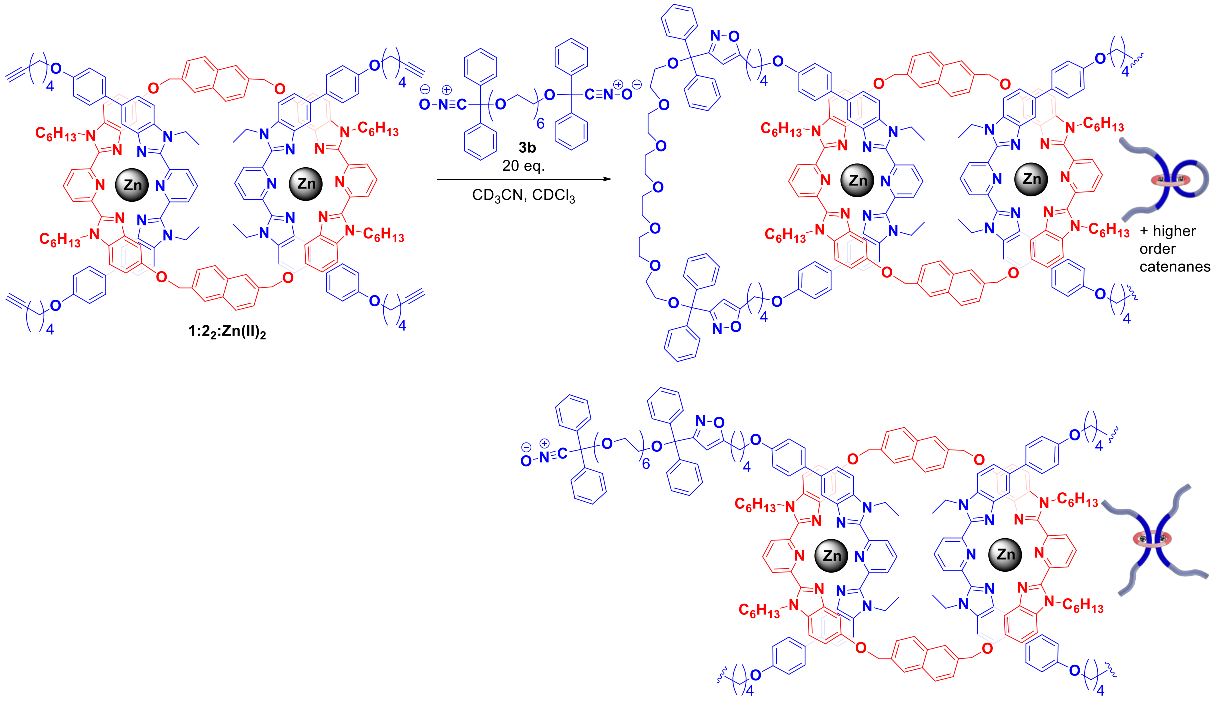

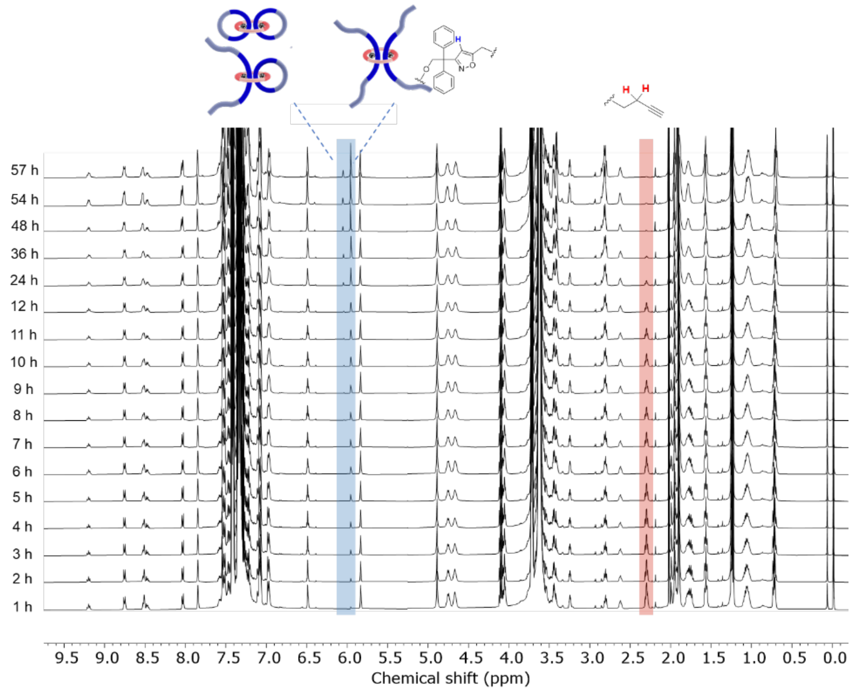

Figure S17. Prepolymerization of **1:2$_2$:Zn(II)$_2$** with an excess amount of monomer **3b** (20 equivalents) in 5% acetonitrile-*d*$_3$ in chloroform-*d* to determine the reaction kinetics and the amount of backbiting. Partial ^{1}H-NMR overlay (500 MHz, 25°C) of prepolymerization of **1:2$_2$:Zn(II)$_2$** with an excess amount of monomer **3b** (20 equivalents) at different reaction times (1 h to 57 h). The disappearance of the signal that corresponds to the α protons of the alkyne (2.30 ppm) shows that **1:2$_2$:Zn(II)$_2$** has been fully reacted. The increase of the signal that corresponds to the protons of the isoxazole (5.95 ppm for linear, and 6.05 ppm for catenane) shows the 18% backbiting.

Monomer **3b** (25.0 mg, 0.0359 mmol) and **1:2$_2$:Zn(II)$_2$** (7.1 mg, 0.00177 mmol) were dissolved in 5% acetonitrile-d_3 in chloroform-*d* (0.5 mL) and added to an NMR tube. The NMR tube was constantly shaken by hand to allow thorough mixing.

Catenane reaction for **1:2$_2$:Zn(II)$_2$** with **3b**

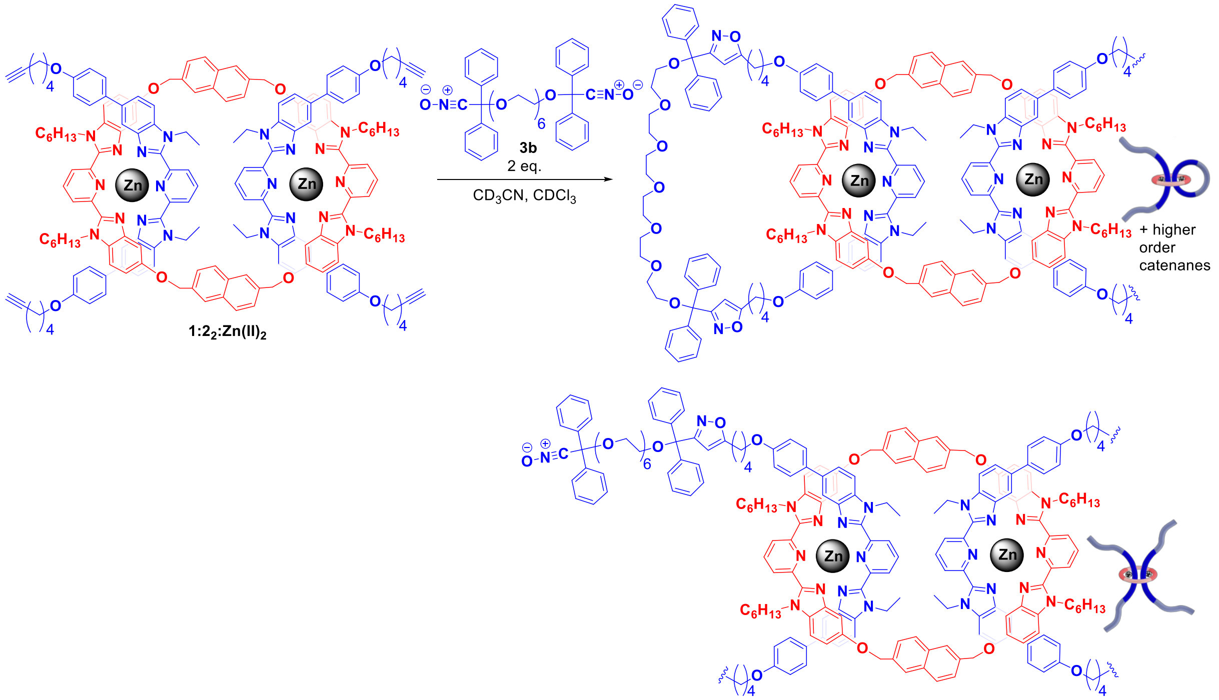

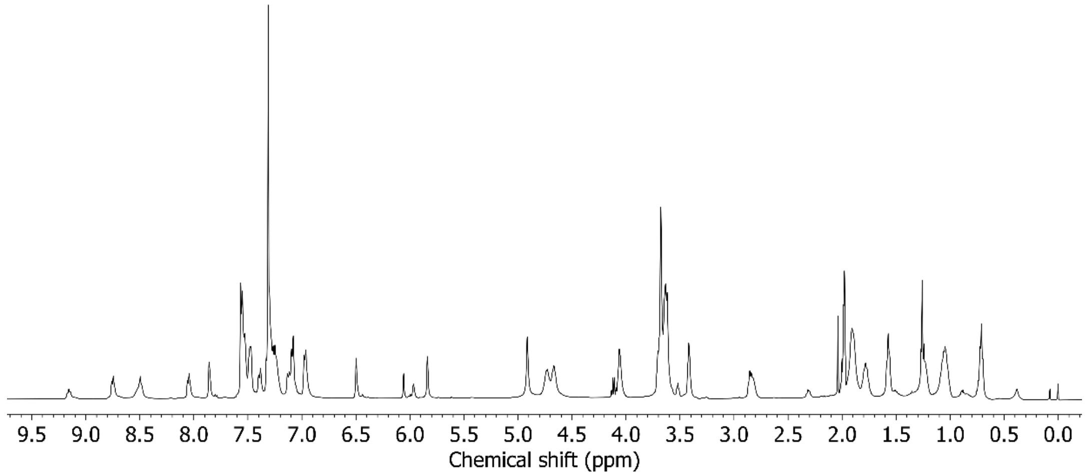

Figure S18. Cyclization of **1:2$_2$:Zn(II)$_2$** with two equivalents of monomer **3b** in 5% acetonitrile-*d*$_3$ in chloroform-*d* to target the catenane. Crude ^{1}H-NMR (500 MHz, 25°C) of cyclization of **1:2$_2$:Zn(II)$_2$** with two equivalents of monomer **3b** in 5% acetonitrile-*d*$_3$ in chloroform-*d* shows an increase in the protons of the isoxazole in the catenane and a decrease in the rotaxane.

Monomer **3b** (5.26 mg, 0.00754 mmol) and **1:2$_2$:Zn(II)$_2$** (15.1 mg, 0.00377 mmol) were dissolved in 5% acetonitrile-*d*$_3$ in chloroform-*d* (0.75 mL) and added to an NMR tube. The mixture was heated to 45 °C for 8 d. The crude NMR was taken. The solvent was removed in vacuo. The residue was dissolved in DCM (10 mL), and acetonitrile (0.5 mL) was added. To demetalate, tetrabutylammonium hydroxide solution (1M in methanol, 100 µL) was added dropwise to demetalate for 30 min. The reaction mixture was then diluted with DCM (5 mL) and washed with water (5 × 20 mL). The organic layer was separated, and the solvent was removed in vacuo.

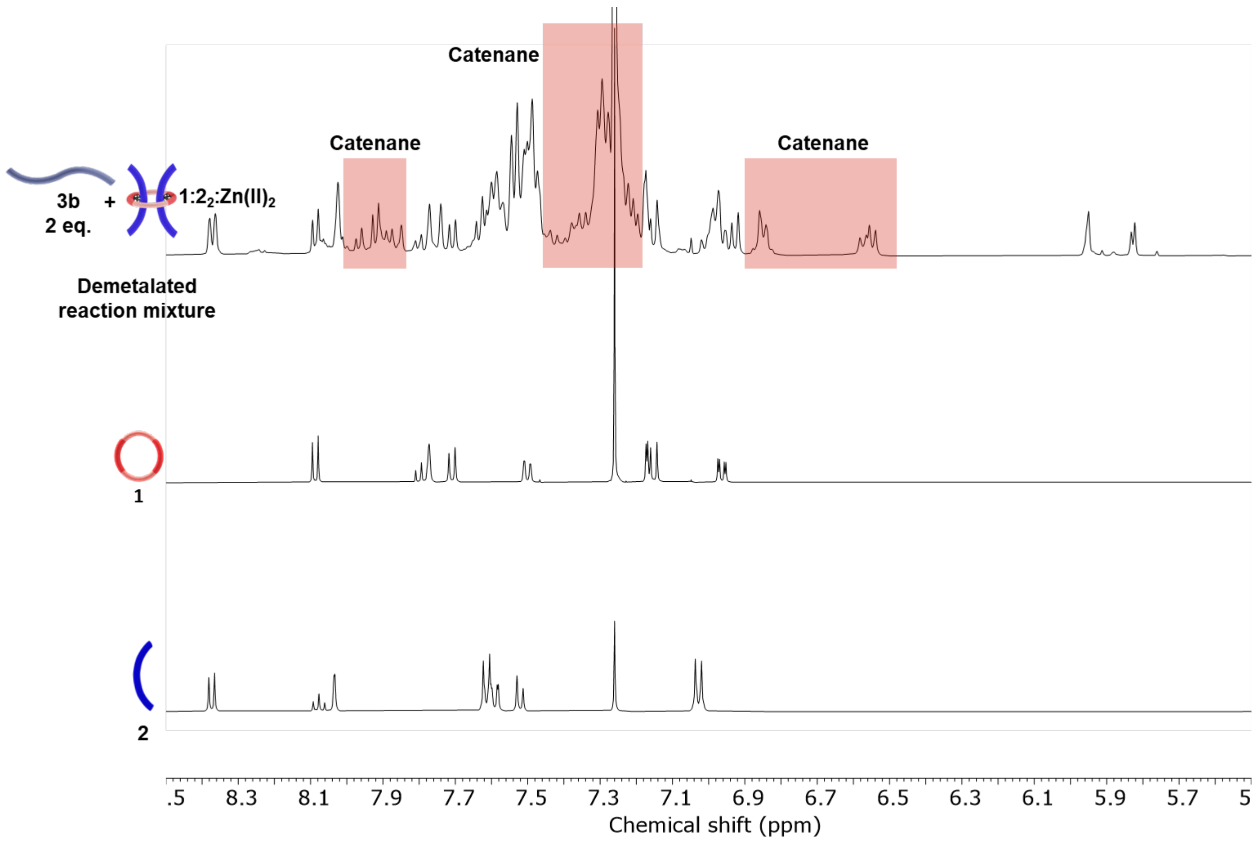

Figure S19. Partial ^{1}H-NMR overlay (500 MHz, $CDCl_3$, 25°C) of **1**, **2**, and the reaction mixture from Figure S18 after demetalation.

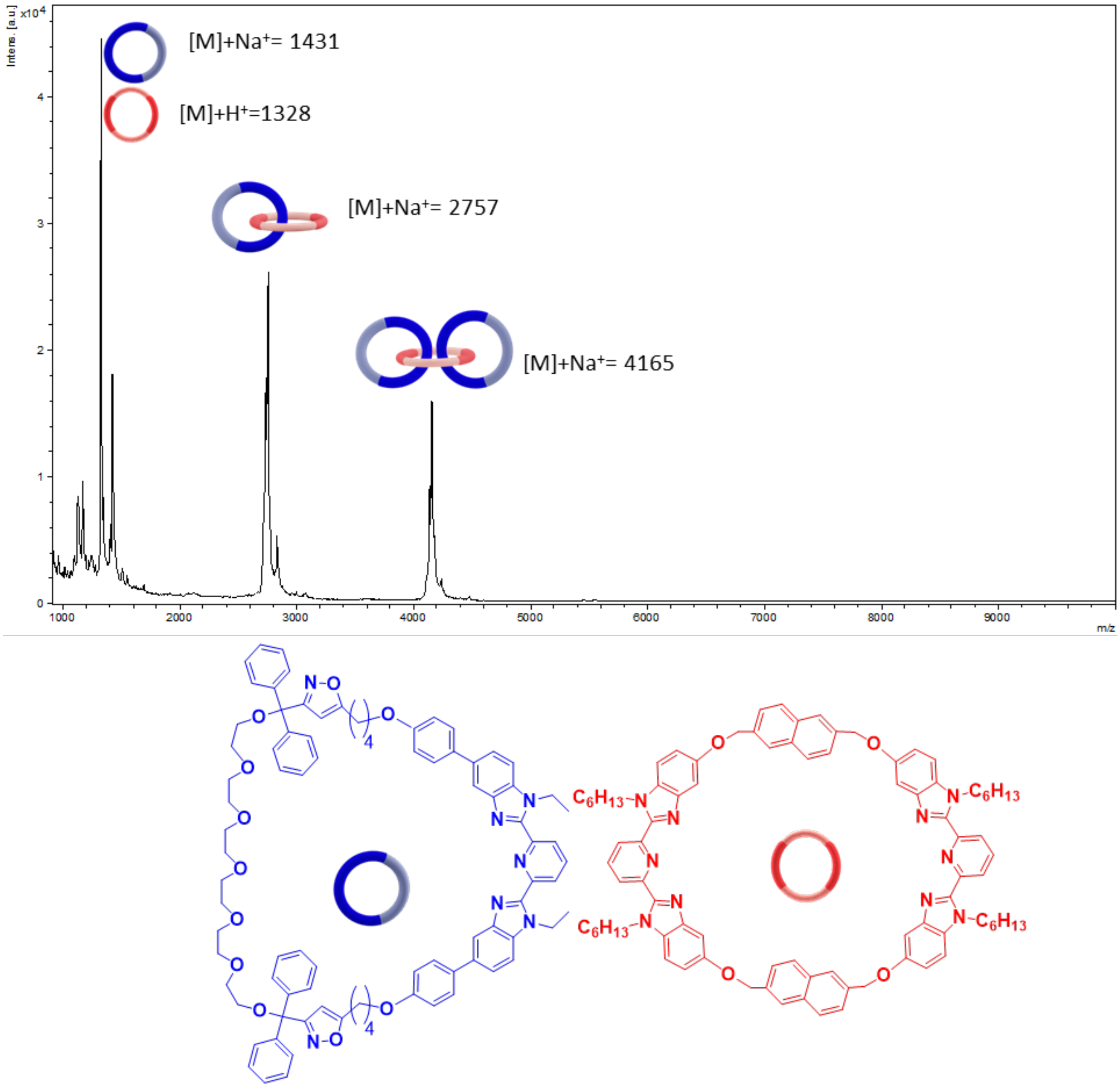

Figure S20. MALDI-TOF mass spectrometry data for the reaction mixture from Figure S18 after demetalation.

<u>Synthesis of propargyl triethylene glycol **16**</u>

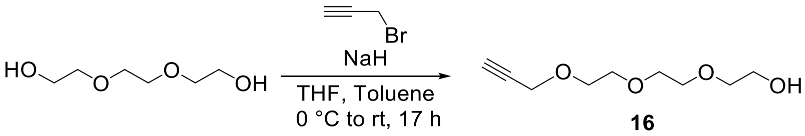

Figure S21. Synthesis of propargyl triethylene glycol **16.**

Triethylene glycol (22.5 g, 150 mmol) was placed in a flame-dried two-neck round-bottom flask under argon atmosphere and dissolved in THF (500 mL). The solution was cooled to 0°C in an ice-water bath, and sodium hydride (90% dry powder, 2.00 g, 75 mmol) was added portionwise to the reaction mixture. The reaction mixture was stirred for 1 h. Then the ice-water bath was removed. The reaction mixture warmed to room temperature and was stirred for another 1 h. Propargyl bromide (80 wt.% in toluene, 8.35 mL, 75.0 mmol) was added dropwise to the reaction

mixture. The reaction mixture was stirred for 15 h. The reaction mixture was filtered, and the solvent was removed in vacuo. The oil was purified by silica gel chromatography (10-90% ethyl acetate in hexanes) to yield propargyl triethylene glycol **16** as a transparent oil (8.23 g, 58% isolated yield). The ^{1}H NMR and ^{13}C NMR data was consistent with the previously reported compound.[12]

Synthesis of propargyl hexaethylene glycol **17**

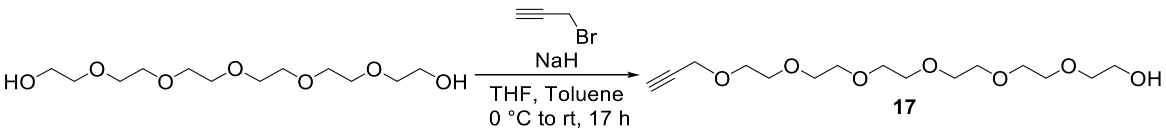

Figure S22. Synthesis of propargyl hexaethylene glycol **17.**

Propargyl hexaethylene glycol **17** was synthesized in the same way as propargyl triethylene glycol **16** with hexaethylene glycol (25.0 g, 88.5 mmol), sodium hydride (90% dry powder, 1.18 g, 44.3 mmol), THF (300 mL) and propargyl bromide (80 wt.% in toluene, 4.94 mL, 44.3 mmol). The product was purified by silica gel chromatography (10-90% ethyl acetate in hexanes to 0-5% methanol in ethyl acetate) to yield propargyl hexaethylene glycol **17** as a pale yellow oil (9.94 g, 70% isolated yield). The ^{1}H NMR and ^{13}C NMR data was consistent with the previously reported compound.[13]

Synthesis of propargyl nonaethylene glycol **18**

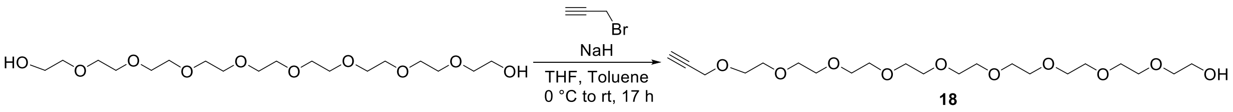

Figure S23. Synthesis of propargyl nonaethylene glycol **18.**

Propargyl nonaethylene glycol **18** was synthesized in the same way as propargyl triethylene glycol **16** with 3,6,9,12,15,18,21,24-octaoxahexacosane-1,26-diol (32.0 g, 77.2 mmol), THF (260 mL), sodium hydride (90% dry powder, 1.03 g, 38.6 mmol), and propargyl bromide (80 wt.% in toluene, 4.30 mL, 38.6 mmol). The product was purified by silica gel chromatography (10-90% ethyl acetate in hexanes to 0-10% methanol in ethyl acetate) to yield propargyl nonaethylene glycol **18** as a pale yellow oil (9.51 g, 54% isolated yield). ^{1}H NMR (500 MHz, $CDCl_3$) δ 4.20 (s, 2H), 3.73 – 3.63 (m, 34H), 3.61 – 3.58 (m, 2H), 2.85 – 2.77 (m, 1H), 2.45 – 2.41 (m, 1H). $^{13}C\{^{1}H\}$ NMR (126 MHz, $CDCl_3$) 79.78, 74.65, 72.69, 70.72, 70.70, 70.67, 70.66, 70.64, 70.51, 70.42, 69.22, 61.83, 58.50. MALDI-TOF MS: ([M]+Na^+) calc'd for $C_{21}H_{40}O_{10}Na$: 475.252 found: 475.228.

Synthesis of propargyl triethylene glycol tosylate **19**

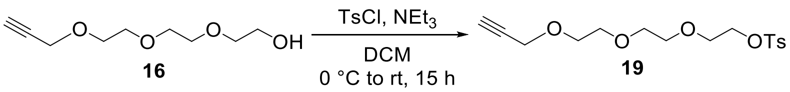

Figure S24. Synthesis of propargyl triethylene glycol tosylate **19.**

Propargyl triethylene glycol **16** (5.00 g, 26.6 mmol) and 4-toluenesulfonyl chloride (10.1 g, 53.0 mmol) were placed in a flame-dried two-neck round-bottom flask under argon atmosphere and dissolved in DCM (170 mL). The solution was cooled to 0°C in an ice-water bath, and triethylamine (11.2 mL, 80.4 mmol) was added dropwise to the reaction mixture. The reaction mixture warmed to room temperature and was stirred for 15 h. The reaction mixture was filtered, and the solvent was removed in vacuo. The residue was dissolved in ethyl acetate, and the residual salt was removed by filtration. The solvent was removed in vacuo, and the oil was purified by silica gel chromatography (10-50% ethyl acetate in hexanes) to yield propargyl triethylene glycol tosylate **19** as a transparent oil (8.10 g, 89% isolated yield). The ^{1}H NMR and ^{13}C NMR data was consistent with the previously reported compound.[14]

Synthesis of propargyl hexaethylene glycol tosylate **20**

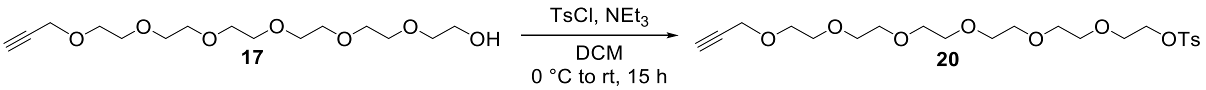

Figure S25. Synthesis of propargyl hexaethylene glycol tosylate **20.**

Propargyl hexaethylene glycol tosylate **20** was synthesized in the same way as propargyl triethylene glycol tosylate **19** with propargyl hexaethylene glycol **17** (5.00 g, 15.6 mmol), 4-toluenesulfonyl chloride (5.95 g, 31.2 mmol), DCM (100 mL), and triethylamine (6.52 mL, 46.8 mmol). The product was purified by silica gel chromatography (10-75% ethyl acetate in hexanes) to yield propargyl hexaethylene glycol tosylate **20** as a pale yellow oil (5.73 g, 77% isolated yield). The ^{1}H NMR and ^{13}C NMR data was consistent with the previously reported compound.[15]

Synthesis of propargyl nonaethylene glycol tosylate **21**

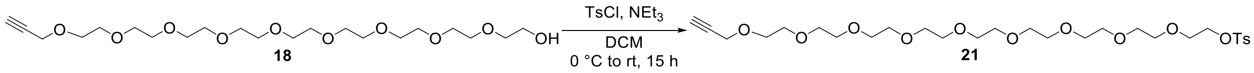

Figure S26. Synthesis of propargyl nonaethylene glycol tosylate **21.**

Propargyl nonaethylene glycol tosylate **21** was synthesized in the same way as propargyl triethylene glycol tosylate **19** with propargyl nonaethylene glycol **18** (3.00 g, 6.63 mmol), 4-toluenesulfonyl chloride (2.54 g, 13.3 mmol), DCM (44 mL), and triethylamine (2.77 mL, 19.9 mmol). The product was purified by silica gel chromatography (10-90% ethyl acetate in hexanes to 0-5% methanol in ethyl acetate) to yield propargyl nonaethylene glycol tosylate **21** as a pale yellow oil (3.00 g, 75% isolated yield). ^{1}H NMR (500 MHz, $CDCl_3$) δ 7.79 (d, *J* = 8.0 Hz, 2H), 7.34 (d, *J* = 8.0 Hz, 2H), 4.20 (d, *J* = 2.5 Hz, 2H), 4.15 (t, *J* = 4.7 Hz, 2H), 3.71 – 3.60 (m, 30H), 3.57 (s, 4H), 2.44 (s, 3H), 2.43 (t, *J* = 2.6 Hz, 1H). ^{13}C{^{1}H} NMR (126 MHz, $CDCl_3$) 144.91, 133.17, 129.96, 128.12, 79.81, 74.66, 70.88, 70.75, 70.73, 70.70, 70.65, 70.54, 69.37, 69.25, 68.82, 58.53, 21.78. MALDI-TOF MS: ([M]+Na^{+}) calc'd for $C_{28}H_{46}O_{12}NaS$: 629.261 found: 629.379.

Synthesis of thread **6a**

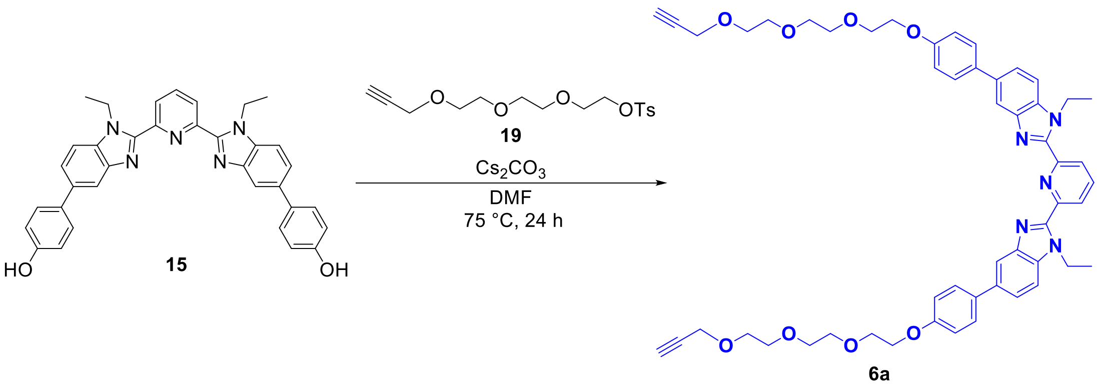

Figure S27. Synthesis of thread **6a.**

Deprotected *N*-ethyl Bip **15** (3.31 g, 6.00 mmol), propargyl triethylene glycol tosylate **19** (5.14 g, 15.0 mmol), and cesium carbonate (7.82 g, 24.0 mmol) were placed in a flame-dried two-neck round-bottom flask under argon atmosphere, and DMF (60 mL) was added. The mixture was heated to 75 °C for 24 h. The solvent was removed in vacuo. The residue was dissolved in chloroform, and the residual salt was removed by filtration. The solvent was removed in vacuo, and the solid was purified by silica gel chromatography (0-2% methanol in chloroform) followed by precipitating using excess cold methanol from a solution of chloroform to yield thread **6a** as a white solid (3.50 g, 65% isolated yield). ^{1}H NMR (500 MHz, $CDCl_3$) δ 8.37 (d, *J* = 7.8 Hz, 2H), 8.07 (t, *J* = 7.9 Hz, 1H), 8.03 (s, 2H), 7.65 – 7.56 (m, 6H), 7.52 (d, *J* = 8.4 Hz, 2H), 7.04 (d, *J* = 8.4 Hz, 4H), 4.83 (q, *J* = 7.2 Hz, 4H), 4.24 – 4.16 (m, 8H), 3.90 (t, *J* = 4.9 Hz, 4H), 3.81 – 3.74 (m, 4H), 3.74 – 3.68 (m, 12H), 2.43 (t, *J* = 2.4 Hz, 2H), 1.40 (t, *J* = 7.2 Hz, 6H). ^{13}C{^{1}H} NMR (126 MHz, $CDCl_3$) 158.28, 150.51, 150.11, 143.65, 138.26, 136.33, 135.29, 134.60, 128.50, 125.82, 123.31,

118.24, 115.17, 110.46, 79.81, 74.65, 70.99, 70.83, 70.61, 69.93, 69.27, 67.71, 58.55, 40.07, 15.62. MALDI-TOF MS: ([M]+H+) calc'd for $C_{53}H_{58}N_5O_8$: 892.429 found: 892.359.

Synthesis of thread **6b**

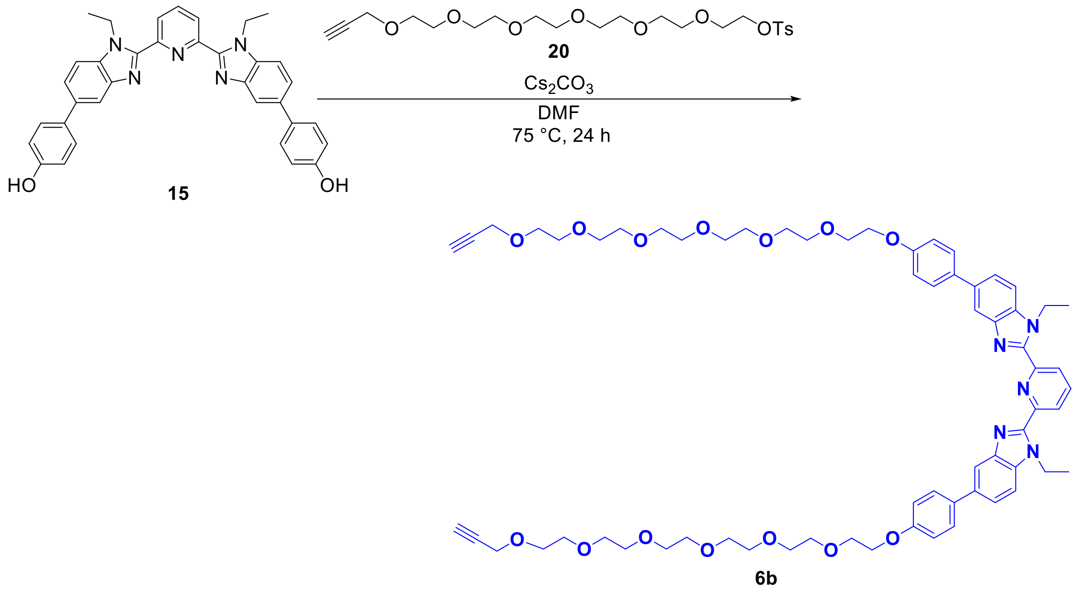

Figure S28. Synthesis of thread **6b.**

Thread **6b** was synthesized in the same way as thread **6a** with deprotected *N*-ethyl Bip **15** (2.21g, 4.00 mmol), propargyl hexaethylene glycol tosylate **20** (4.74 g, 10.0 mmol), cesium carbonate (5.21 g, 16.0 mmol), and DMF (40 mL). The product was purified by silica gel chromatography (2-4% methanol in chloroform) followed by precipitating using excess cold methanol from a solution of chloroform to yield thread **6b** as a white solid (3.24 g, 70% isolated yield). ^{1}H NMR (500 MHz, $CDCl_3$) δ 8.37 (d, *J* = 7.9 Hz, 2H), 8.07 (t, *J* = 7.9 Hz, 1H), 8.03 (d, *J* = 1.6 Hz, 2H), 7.63 – 7.56 (m, 6H), 7.51 (d, *J* = 8.4 Hz, 2H), 7.03 (d, *J* = 8.7 Hz, 4H), 4.83 (q, *J* = 7.2 Hz, 4H), 4.27 – 4.15 (m, 8H), 3.90 (q, *J* = 5.6, 4.9, 4.2 Hz, 4H), 3.77 – 3.74 (m, 4H), 3.72 – 3.63 (m, 36H), 2.43 (t, *J* = 2.4 Hz, 2H), 1.40 (t, *J* = 7.2 Hz, 6H). ^{13}C{^{1}H} NMR (126 MHz, $CDCl_3$) 158.27, 150.51, 150.10, 143.64, 138.27, 136.33, 135.29, 134.59, 128.50, 125.82, 123.31, 118.23, 115.16, 110.47, 79.81, 74.66, 71.00, 70.79, 70.75, 70.73, 70.70, 70.54, 69.91, 69.25, 67.70, 58.53, 40.08, 15.63. MALDI-TOF MS: ([M]+H+) calc'd for $C_{65}H_{82}N_5O_{14}$: 1156.586 found: 1156.576.

Synthesis of thread **6c**

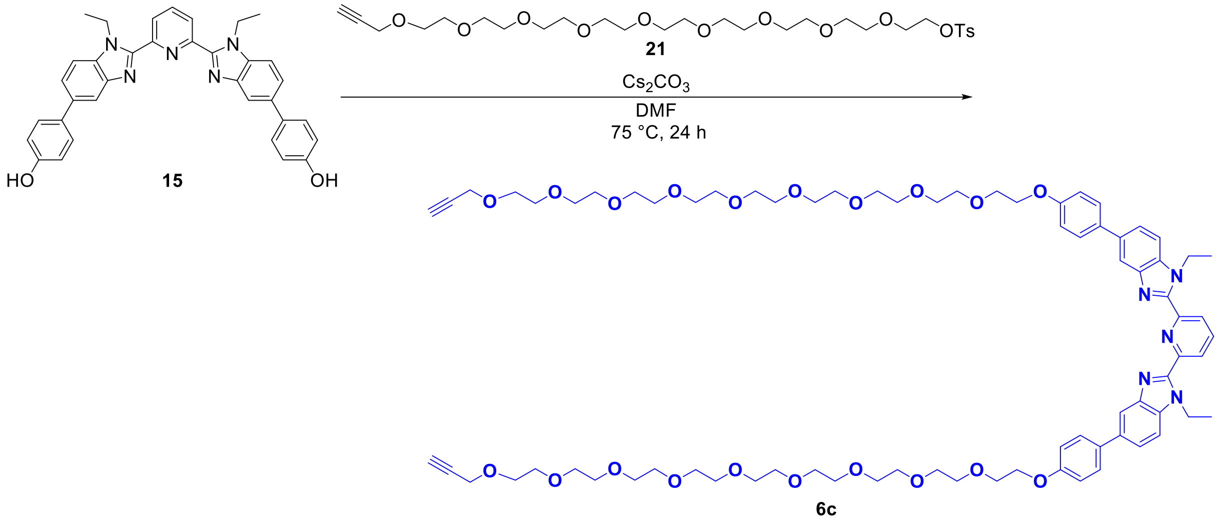

Figure S29. Synthesis of thread **6c.**

Thread **6c** was synthesized in the same way as thread **6a** with deprotected *N*-ethyl Bip **15** (1.10g, 2.00 mmol), propargyl nonaethylene glycol tosylate **21** (3.03 g, 5.00 mmol), cesium carbonate (2.61 g, 8.00 mmol), and DMF (20 mL). The product was purified by silica gel chromatography (2-5% methanol in chloroform) followed by precipitating using excess cold methanol from a solution of chloroform to yield thread **6c** as a white solid (2.08 g, 73% isolated yield). ^1H NMR (500 MHz, $CDCl_3$) δ 8.35 (d, *J* = 7.8 Hz, 2H), 8.05 (t, *J* = 7.9 Hz, 1H), 8.01 (s, 2H), 7.63 – 7.53 (m, 6H), 7.49 (d, *J* = 8.4 Hz, 2H), 7.01 (d, *J* = 8.7 Hz, 4H), 4.81 (q, *J* = 7.2 Hz, 4H), 4.20 – 4.14 (m, 8H), 3.87 (t, *J* = 4.8 Hz, 4H), 3.75 – 3.72 (m, 4H), 3.69 – 3.61 (m, 60H), 2.42 (t, *J* = 2.4 Hz, 2H), 1.38 (t, *J* = 7.1 Hz, 6H). $^{13}C\{^1H\}$ NMR (126 MHz, $CDCl_3$) 158.19, 150.40, 150.02, 143.56, 138.16, 136.21, 135.20, 134.48, 128.38, 125.73, 123.20, 118.12, 115.07, 110.39, 79.72, 74.62, 70.90, 70.69, 70.64, 70.62, 70.44, 69.81, 69.14, 67.60, 58.43, 39.98, 15.54. MALDI-TOF MS: ([M]+H^+) calc'd for $C_{77}H_{106}N_5O_{20}$: 1420.743 found: 1420.806.

^1H NMR and ^{13}C NMR for intermediates and components

^1H NMR spectrum of propargyl nonaethylene glycol **18**

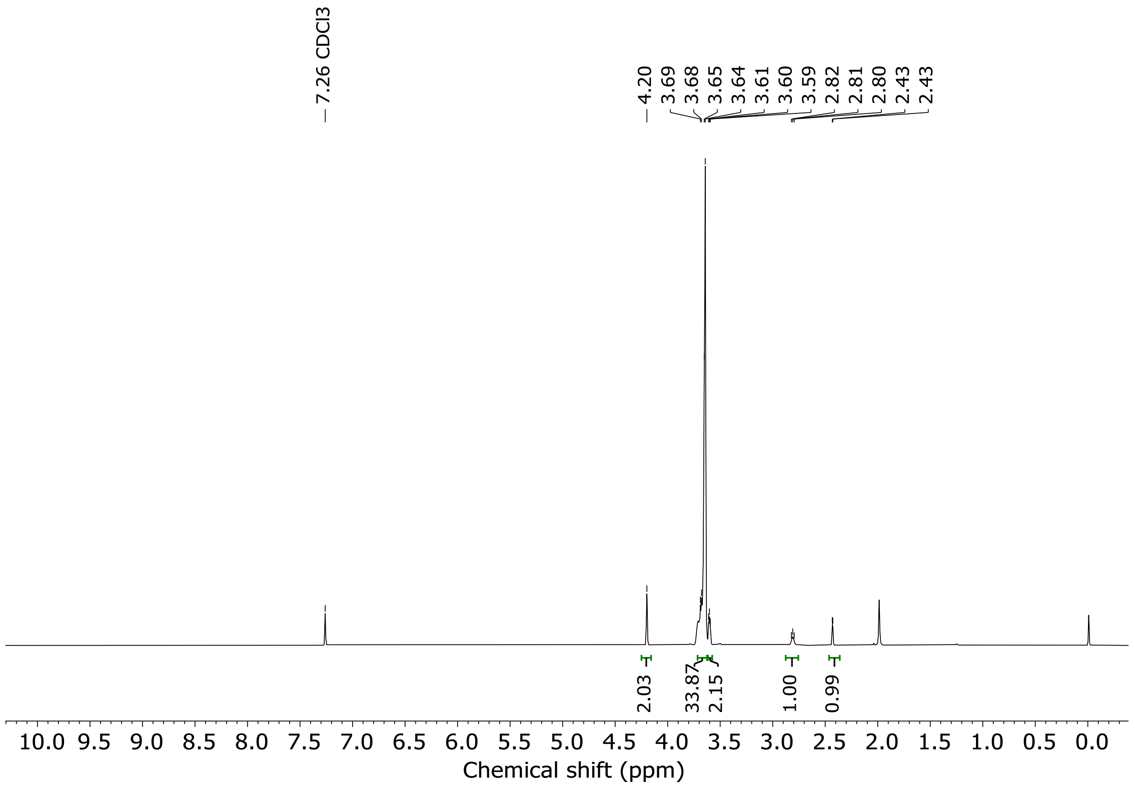

^{13}C NMR spectrum of propargyl nonaethylene glycol **18**

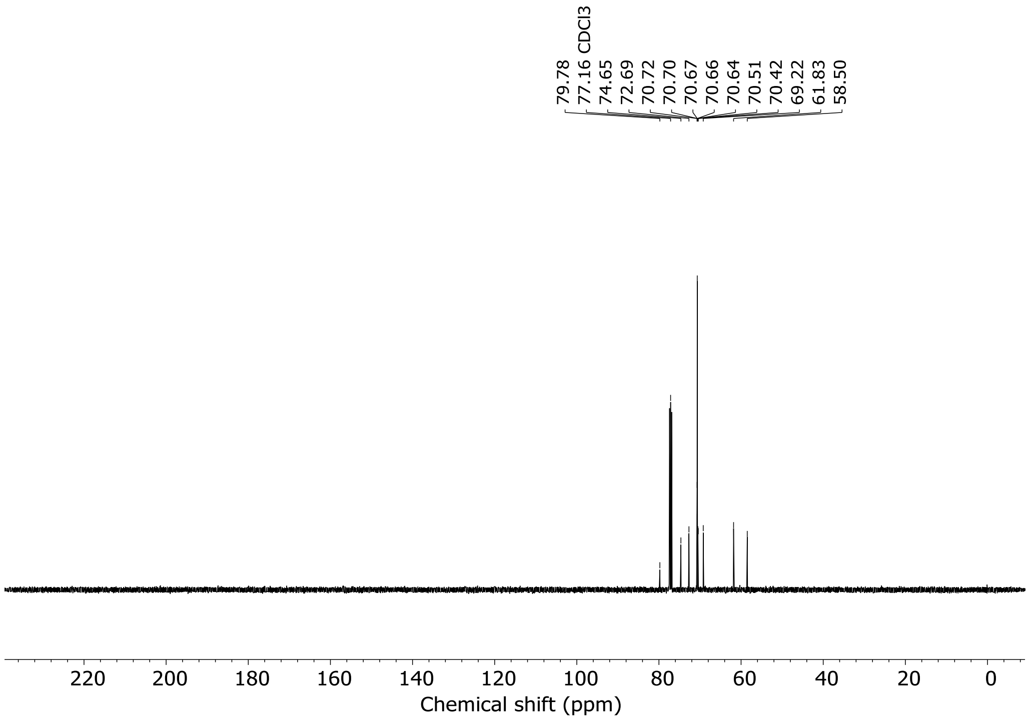

<u>^{1}H NMR spectrum of propargyl nonaethylene glycol tosylate **21**</u>

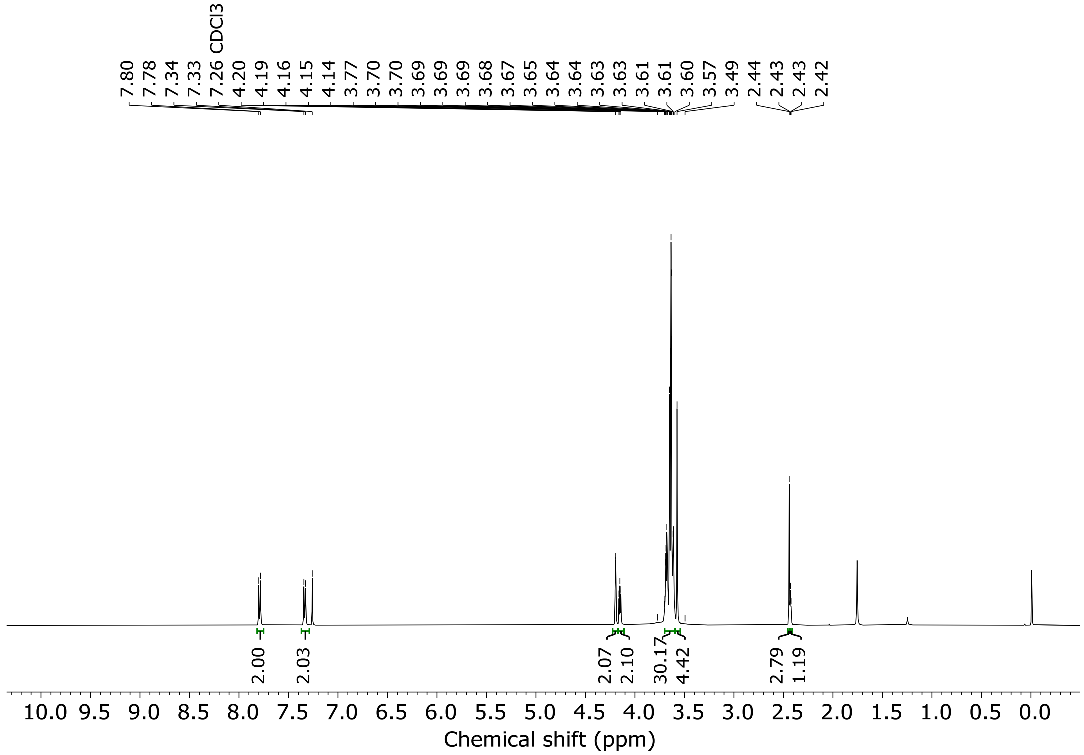

<u>^{13}C NMR spectrum of propargyl nonaethylene glycol tosylate **21**</u>

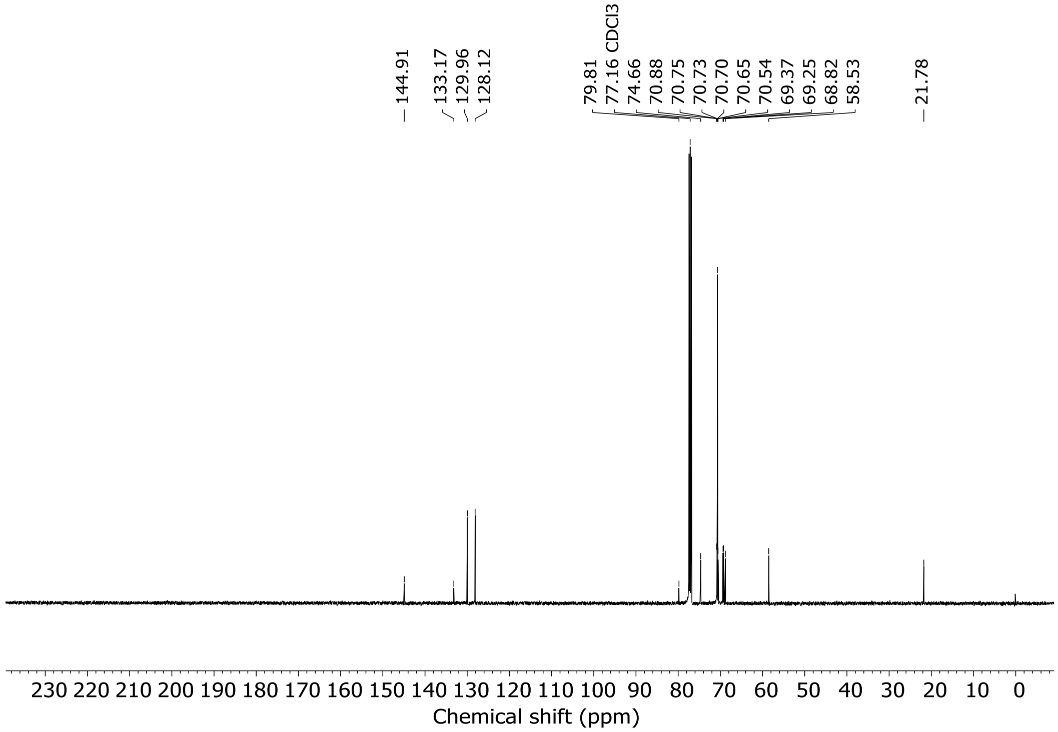

^{1}H NMR spectrum of thread **6a**

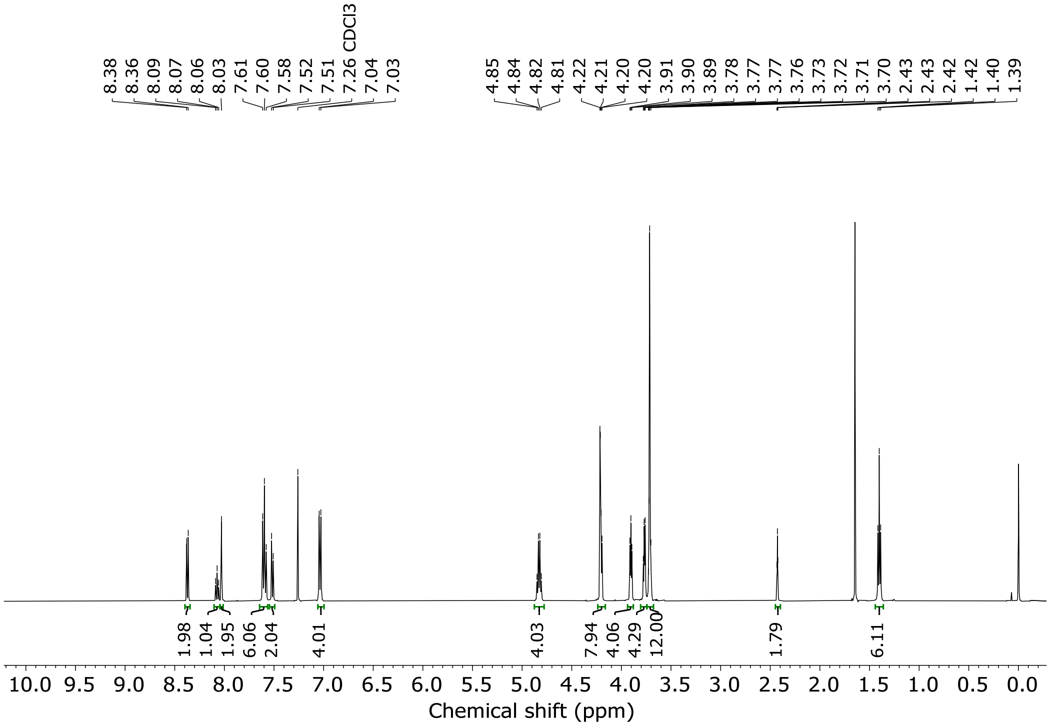

^{13}C NMR spectrum of thread **6a**

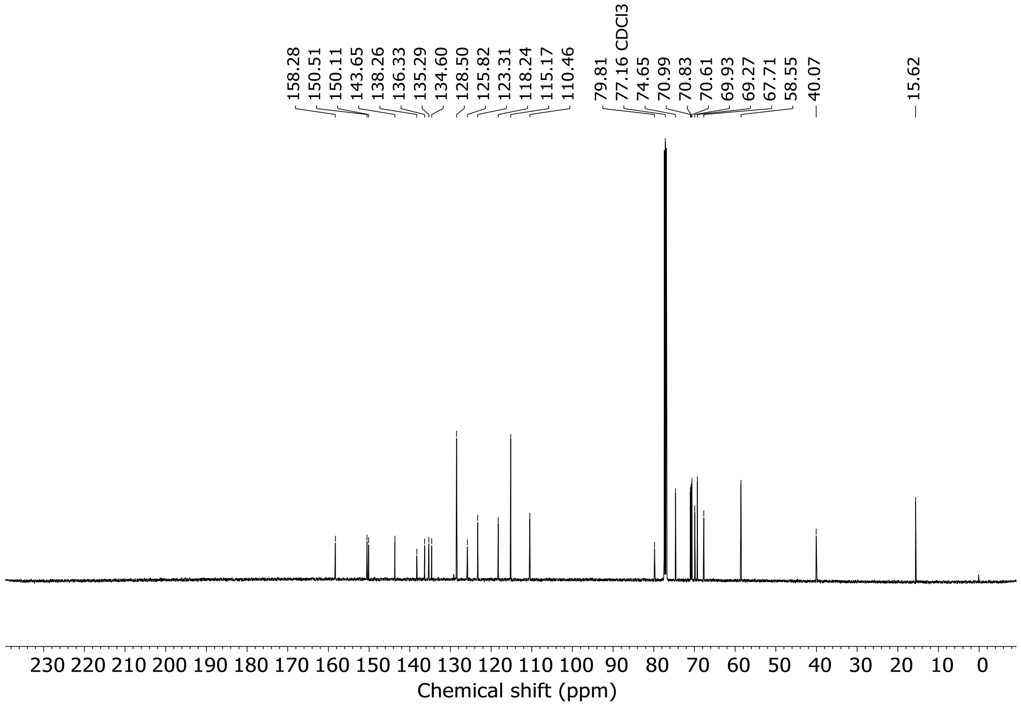

^{1}H NMR spectrum of thread **6b**

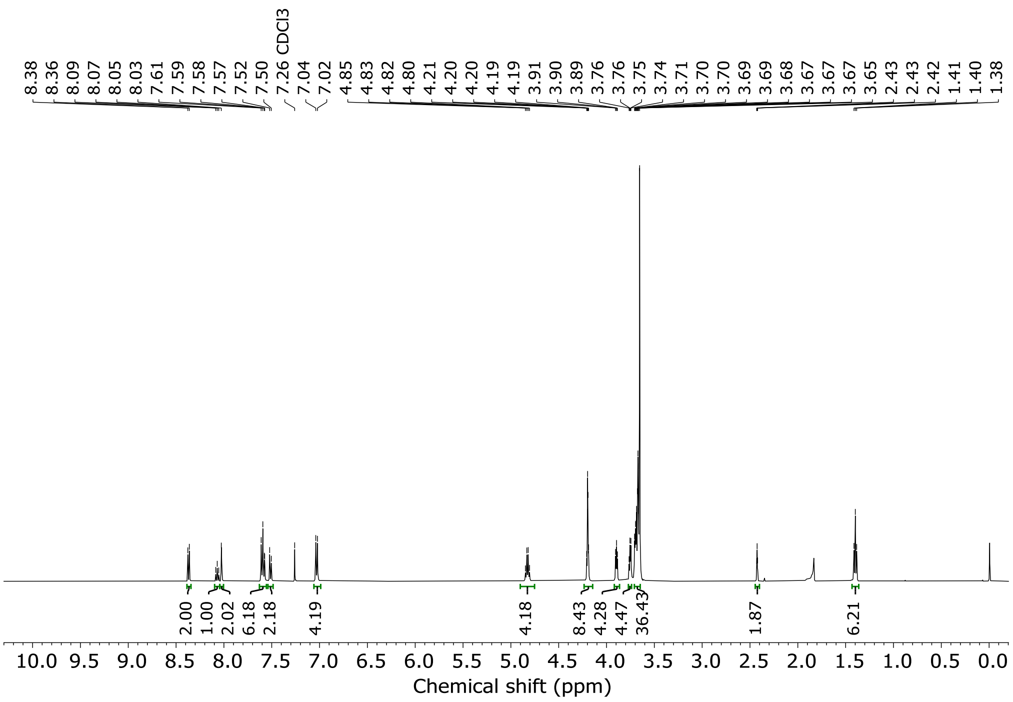

^{13}C NMR spectrum of thread **6b**

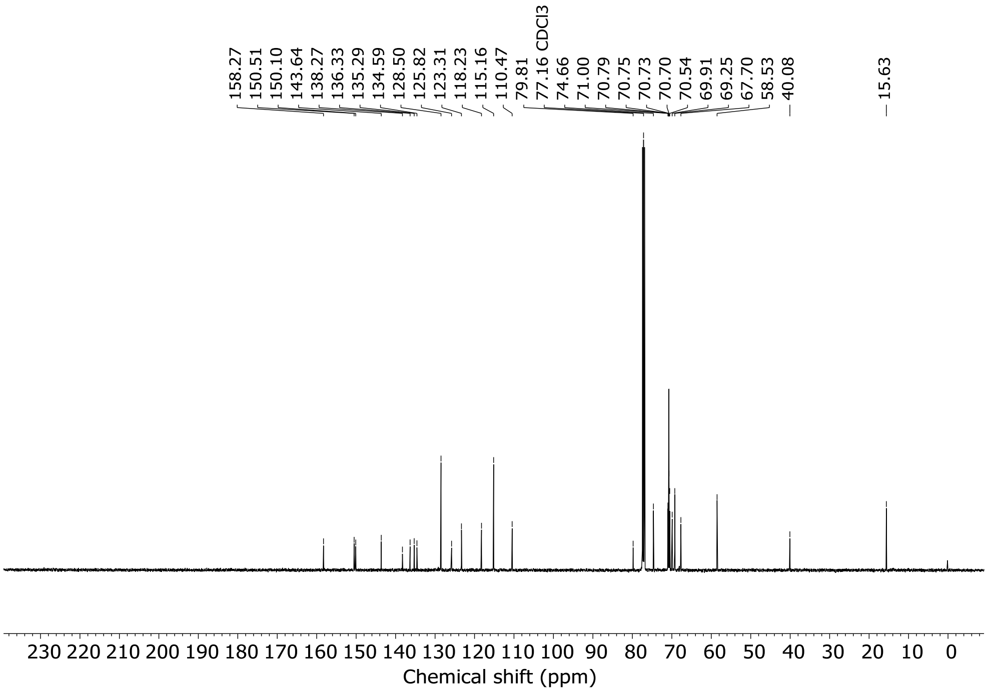

^{1}H NMR spectrum of thread **6c**

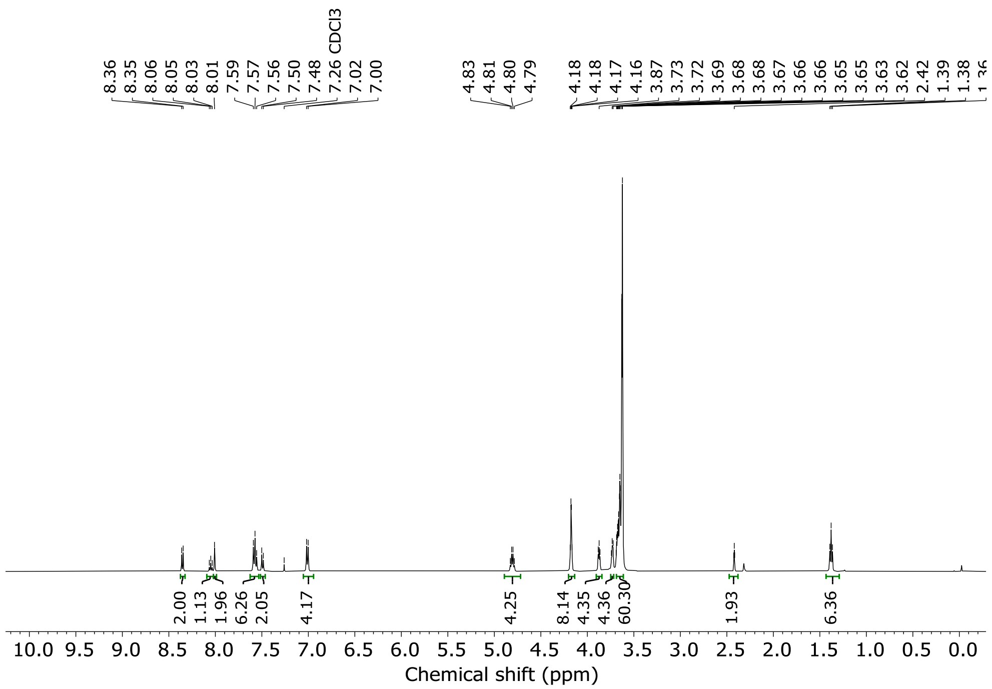

^{13}C NMR spectrum of thread **6c**

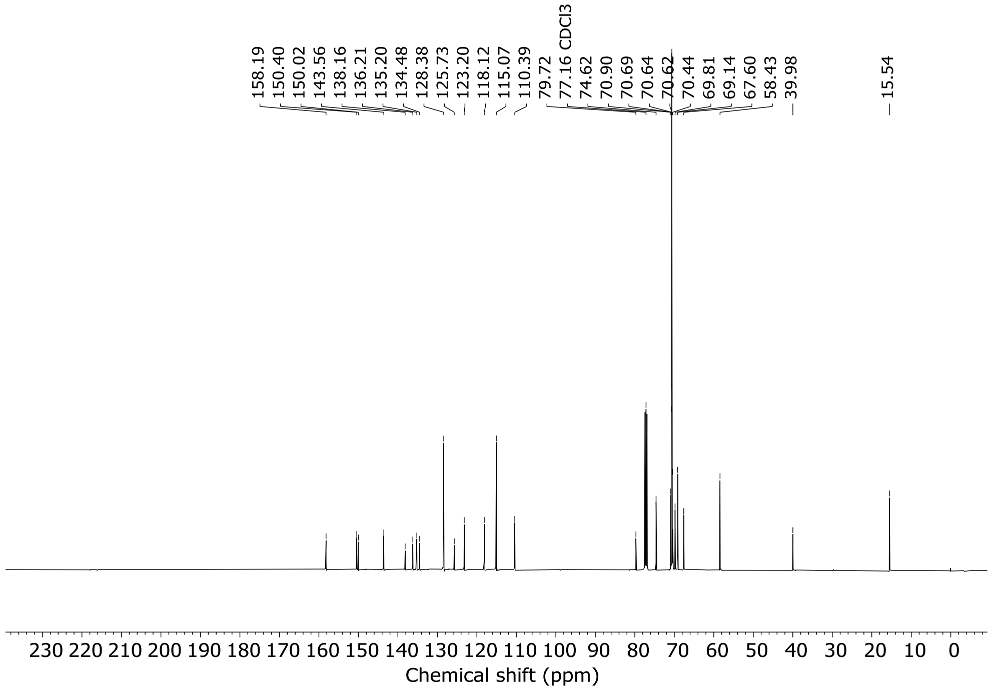

^{1}H NMR spectrum of dinitro hexaethylene glycol **23**

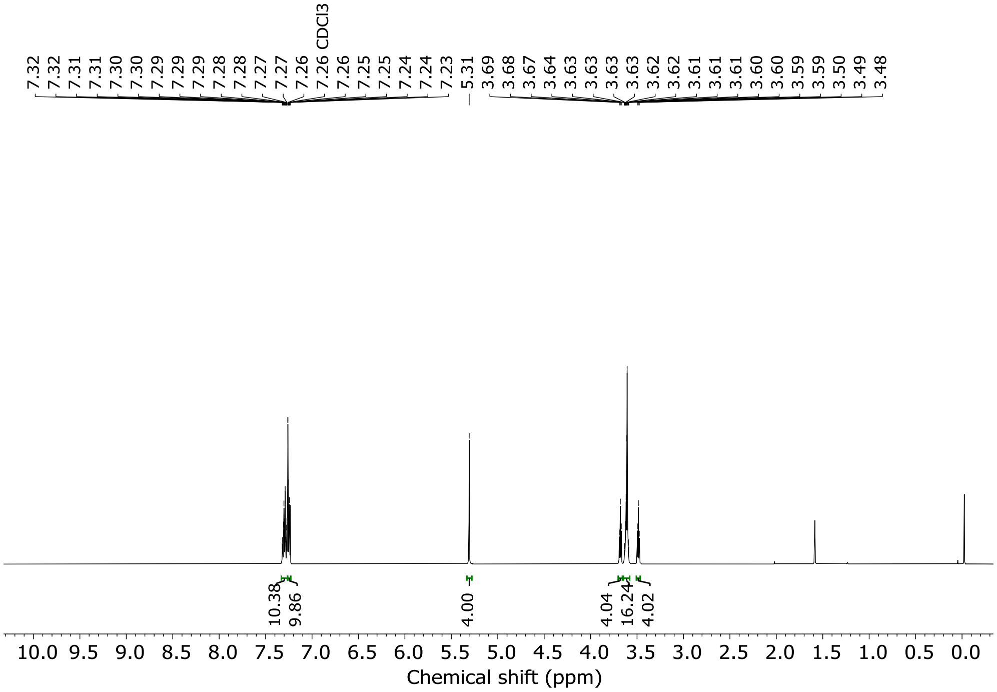

^{13}C NMR spectrum of dinitro hexaethylene glycol **23**

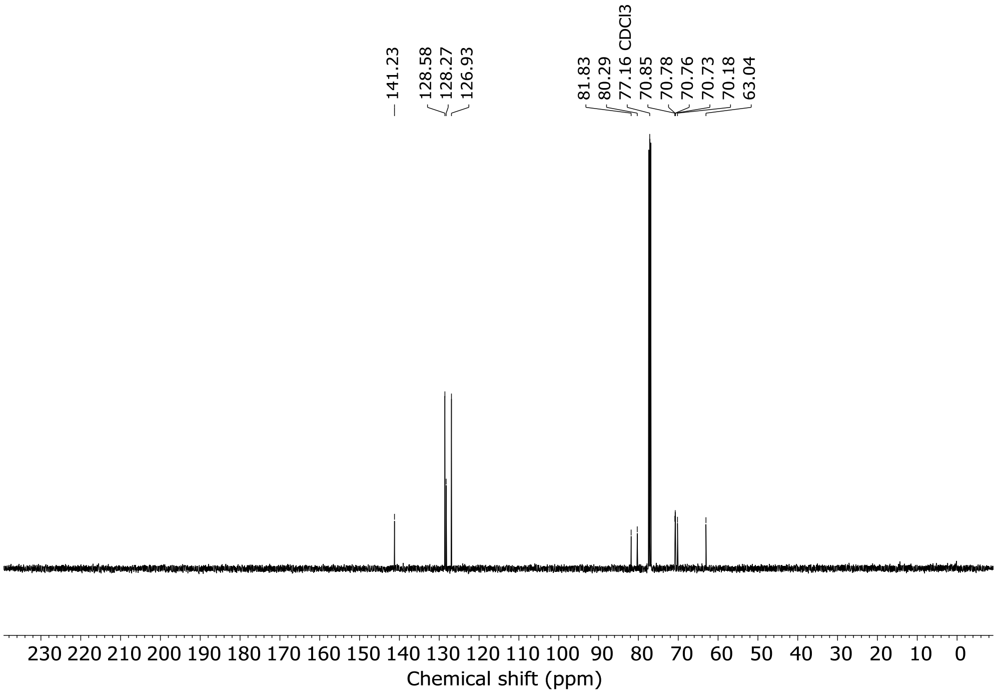

^{1}H NMR spectrum of monomer **3b**

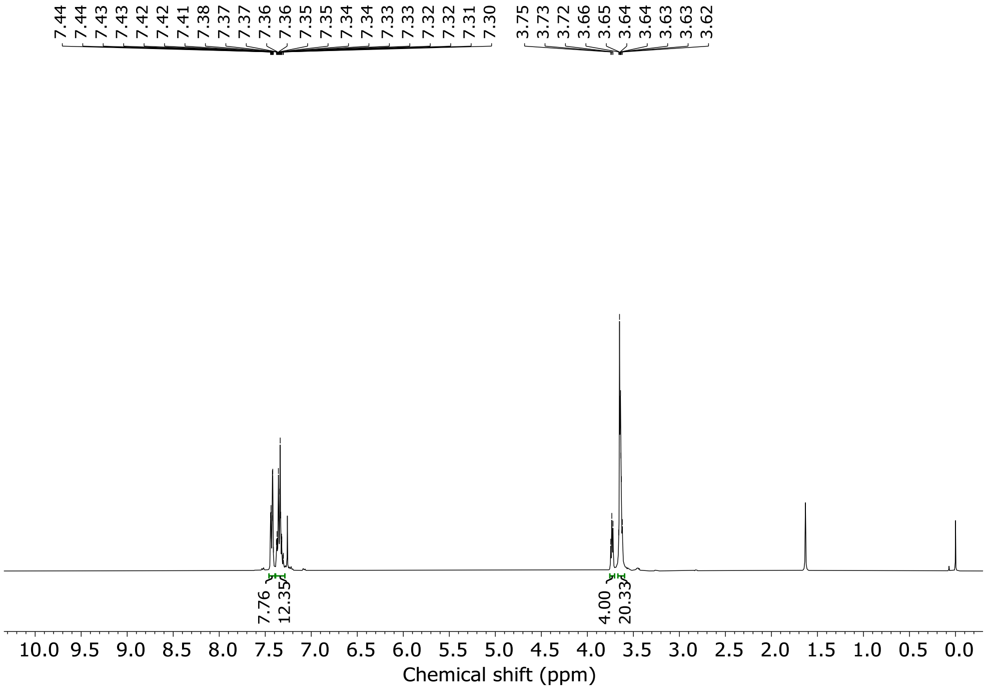

^{13}C NMR spectrum of monomer **3b**

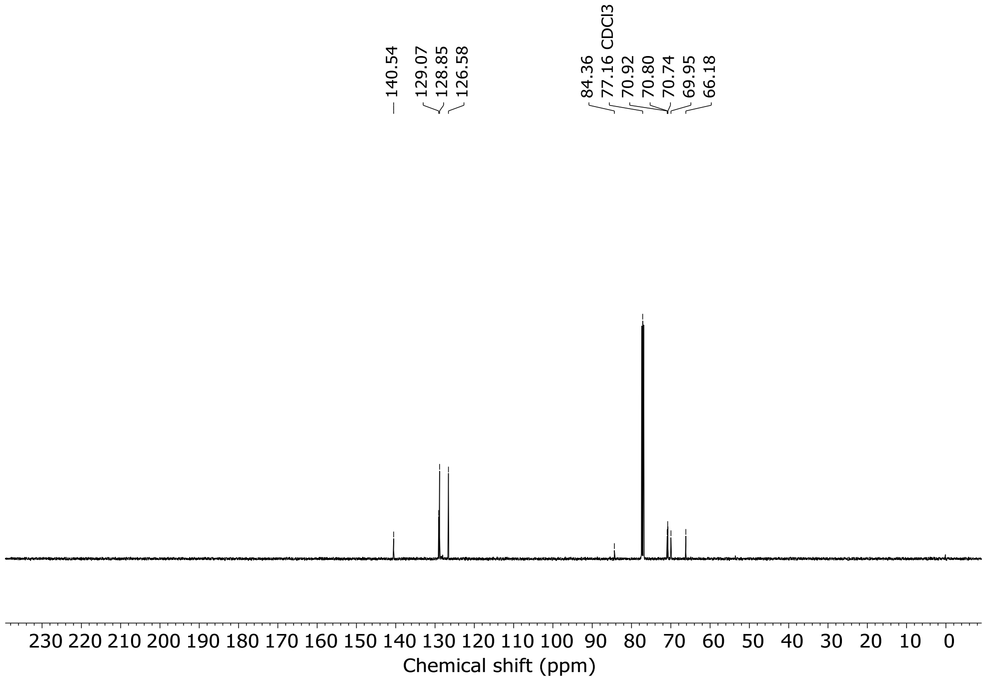

Zn(II) Assembly of Pseudo[3]rotaxane (P3R) **1:6a$_2$:Zn(II)$_2$**

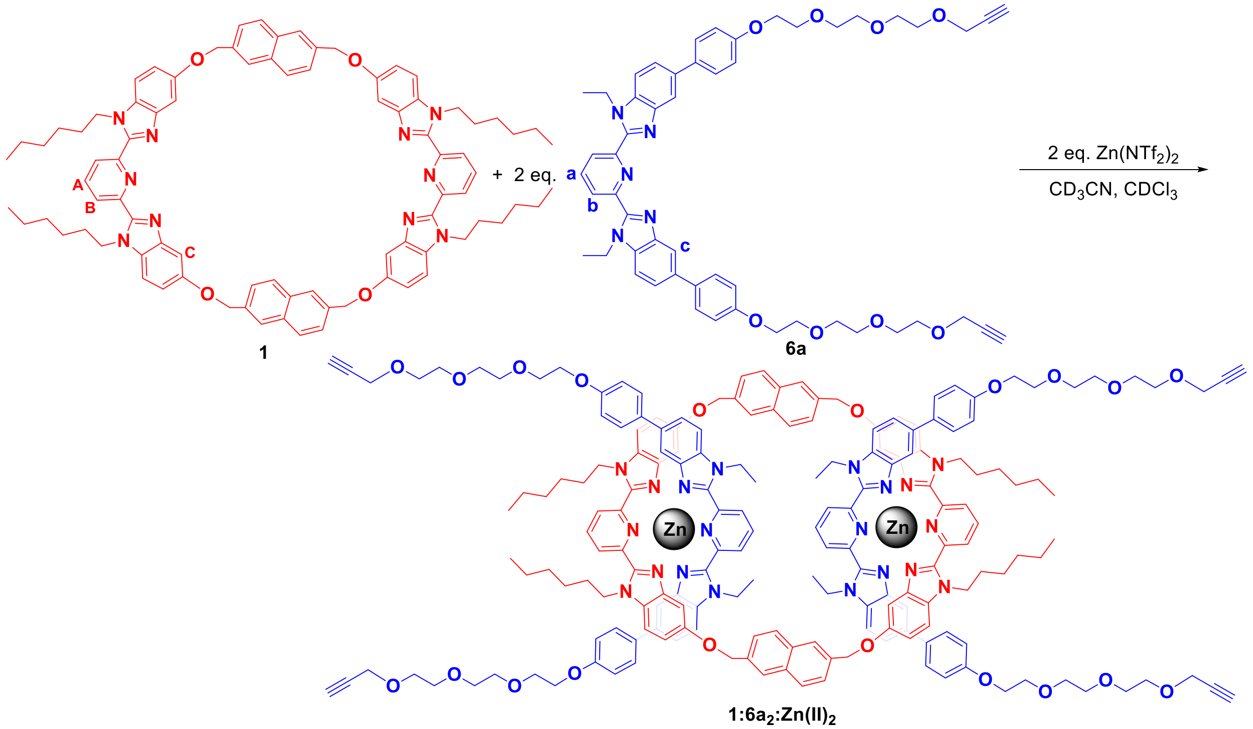

Figure S30. Formation of **1:6a$_2$:Zn(II)$_2$**.

1:6a$_2$:Zn(II)$_2$ was synthesized in the same way as **1:2$_2$:Zn(II)$_2$** with macrocycle **1** (10.0 mg, 0.00753 mmol) in chloroform-*d* (0.5 mL), stock solution of thread **6** (16.1 mg, 0.0181 mmol) in chloroform-*d* (0.6 mL), and stock solution of zinc di[bis(trifluoromethylsulfonyl)imide] (11.3 mg, 0.0181 mmol) in acetonitrile-*d*$_3$ (0.6 mL). The resulting pseudo[3]rotaxane **1:6a$_2$:Zn(II)$_2$** solution was dried in vacuo to obtain a yellow solid that was redissolved in 1 mL 5% acetonitrile-*d*$_3$ in chloroform-*d*, and stirred at 45°C for 1 d to allow equilibration. The solvent was then removed in vacuo, resulting in a yellow waxy solid that was stored in the freezer at -37°C before use.

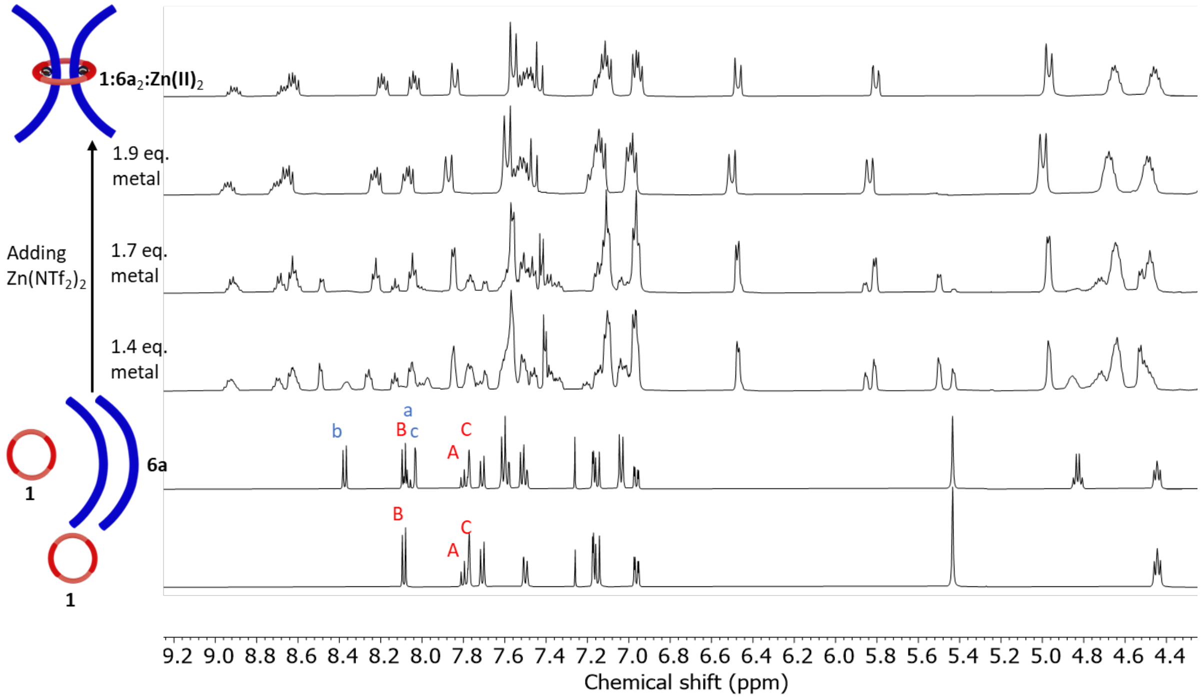

Figure S31. Partial ^{1}H-NMR overlay (500 MHz, 25°C, Solvent: increasing acetonitrile-*d*$_3$ in chloroform-*d* increasing upwards) of metal addition during NMR titrations.

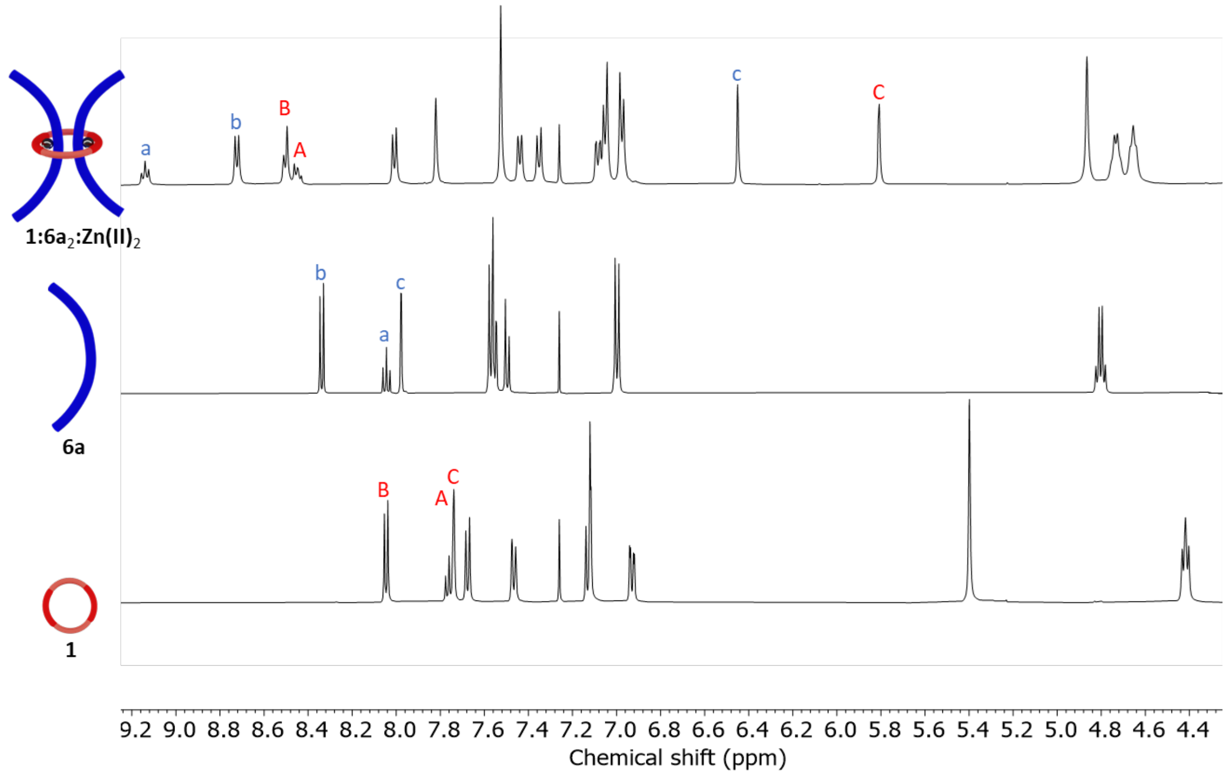

Figure S32. Partial ^{1}H-NMR overlay (500 MHz, 25°C) of 10 mM **1:6a$_2$:Zn(II)$_2$**, 20 mM **6a**, and 10 mM **1** (5% acetonitrile-*d*$_3$ in chloroform-*d*) after equilibration.

Zn(II) Assembly of Pseudo[3]rotaxane (P3R) **1:6b$_2$:Zn(II)$_2$**

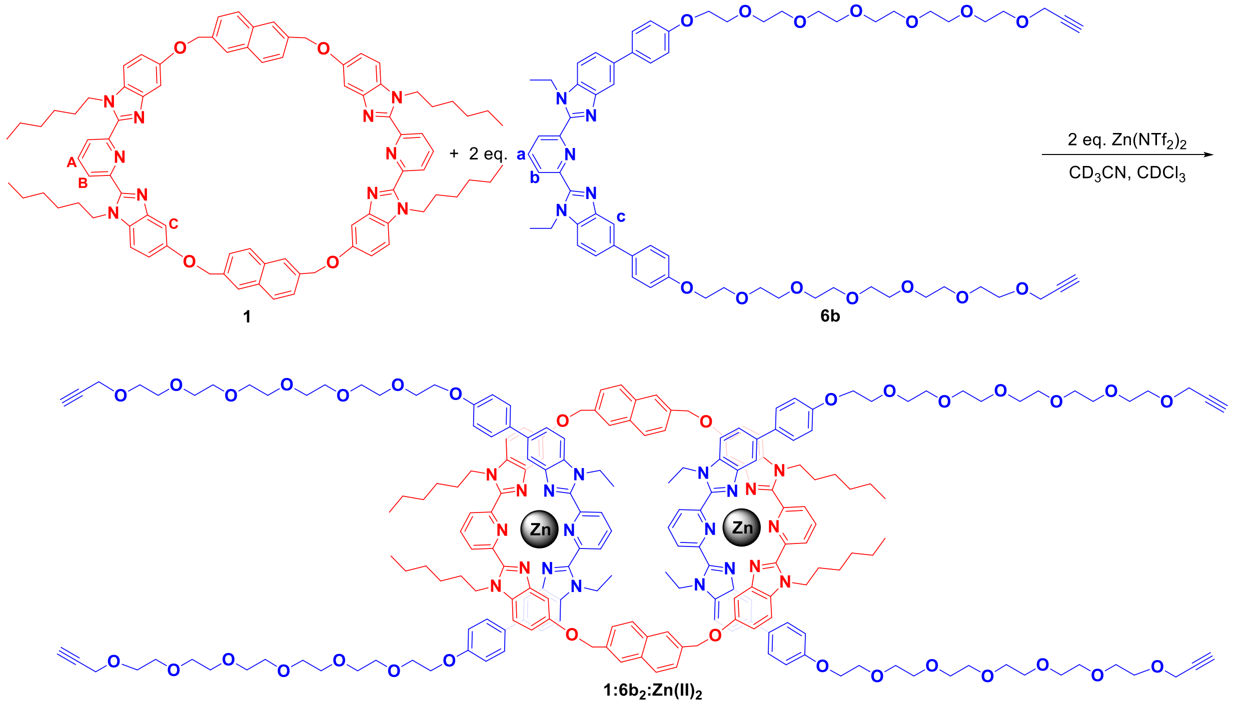

Figure S33. Formation of **1:6b$_2$:Zn(II)$_2$**.

1:6b$_2$:Zn(II)$_2$ was synthesized in the same way as **1:2$_2$:Zn(II)$_2$** with macrocycle **1** (10.0 mg, 0.00753 mmol) in chloroform-*d* (0.5 mL), stock solution of thread **6b** (20.9 mg, 0.0181 mmol) in chloroform-*d* (0.6 mL), and stock solution of zinc di[bis(trifluoromethylsulfonyl)imide] (11.3 mg, 0.0181 mmol) in acetonitrile-d_3 (0.6 mL). The resulting pseudo[3]rotaxane **1:6b$_2$:Zn(II)$_2$** solution was dried in vacuo to obtain a yellow solid that was redissolved in 1 mL 5% acetonitrile-d_3 in chloroform-*d*, and stirred at 45°C for 1 d to allow equilibration. The solvent was then removed in vacuo, resulting in a yellow waxy solid that was stored in the freezer at -37°C before use.

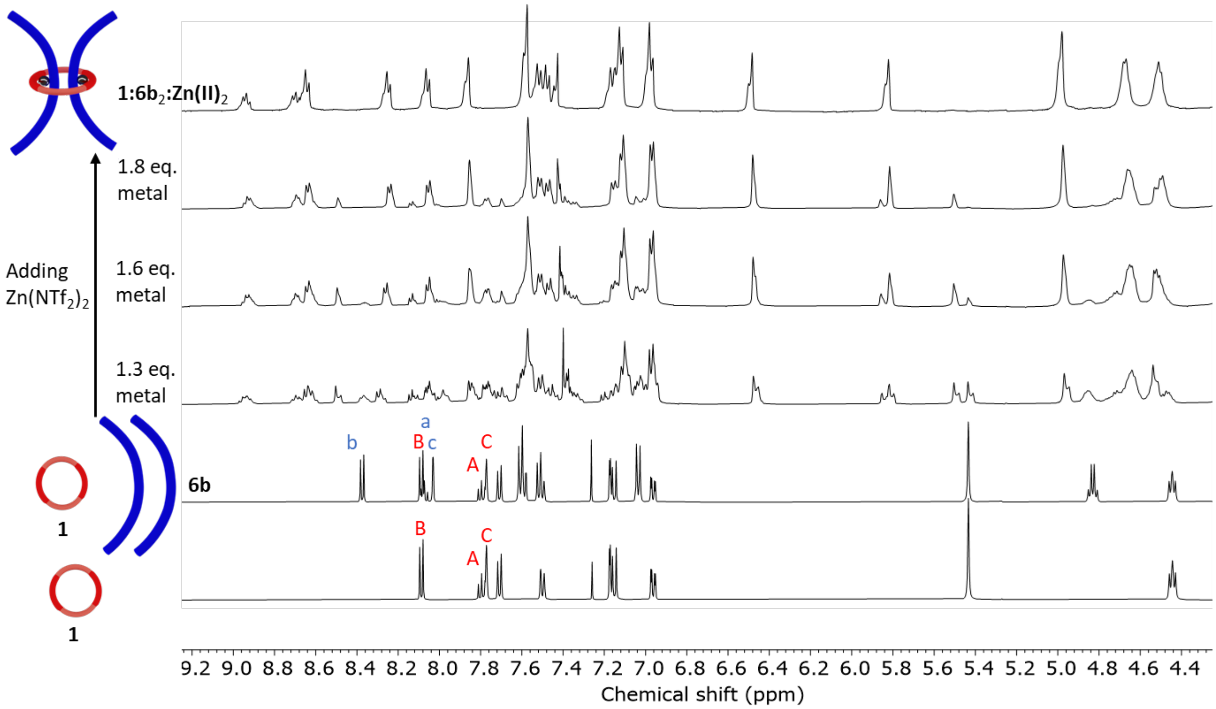

Figure S34. Partial ^{1}H-NMR overlay (500 MHz, 25°C, Solvent: increasing acetonitrile-d_3 in chloroform-d increasing upwards) of metal addition during NMR titrations.

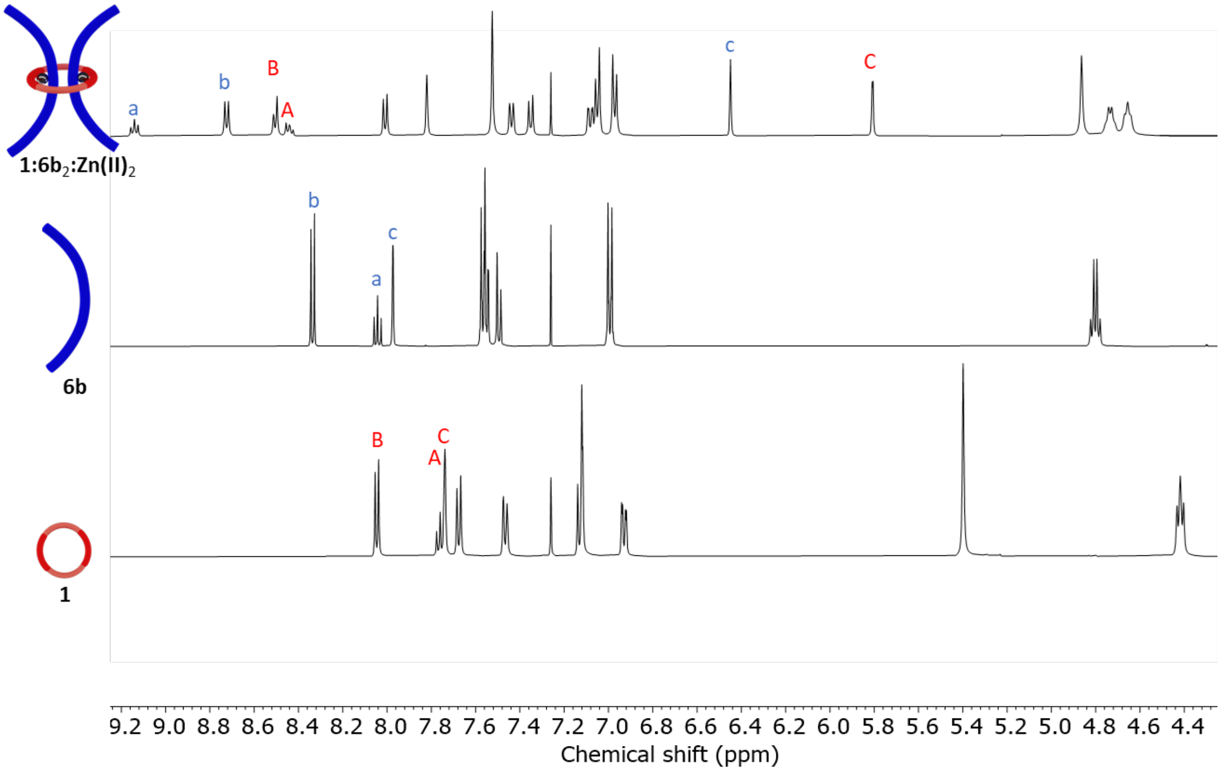

Figure S35. Partial ^{1}H-NMR overlay (500 MHz, 25°C) of 10 mM **1:6b$_2$:Zn(II)$_2$**, 20 mM **6b,** and 10 mM **1** (5% acetonitrile-d_3 in chloroform-d) after equilibration.

Zn(II) Assembly of Pseudo[3]rotaxane (P3R) **1:6c₂:Zn(II)₂**

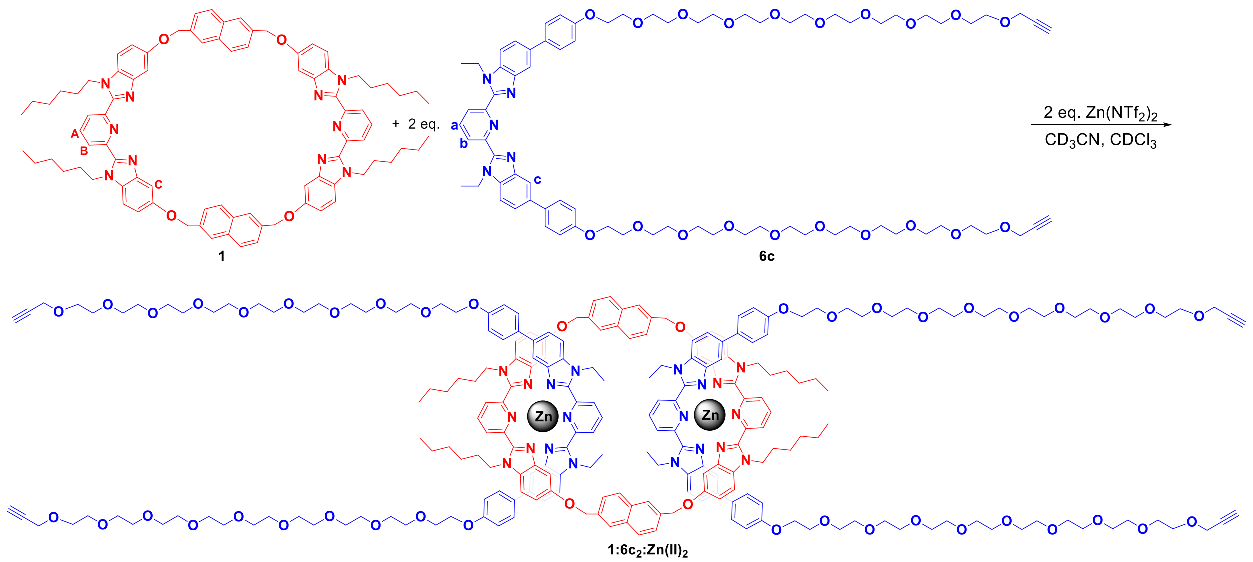

Figure S36. Formation of **1:6c$_2$:Zn(II)$_2$**.

1:6c$_2$:Zn(II)$_2$ was synthesized in the same way as **1:2$_2$:Zn(II)$_2$** with macrocycle **1** (10.0 mg, 0.00753 mmol) in chloroform-*d* (0.5 mL), stock solution of thread **6c** (25.7 mg, 0.0181 mmol) in chloroform-*d* (0.6 mL), and stock solution of zinc di[bis(trifluoromethylsulfonyl)imide] (11.3 mg, 0.0181 mmol) in acetonitrile-d_3 (0.6 mL). The resulting pseudo[3]rotaxane **1:6c$_2$:Zn(II)$_2$** solution was dried in vacuo to obtain a yellow solid that was redissolved in 1 mL 5% acetonitrile-d_3 in chloroform-*d*, and stirred at 45°C for 1 d to allow equilibration. The solvent was then removed in vacuo, resulting in a yellow waxy solid that was stored in the freezer at -37°C before use.

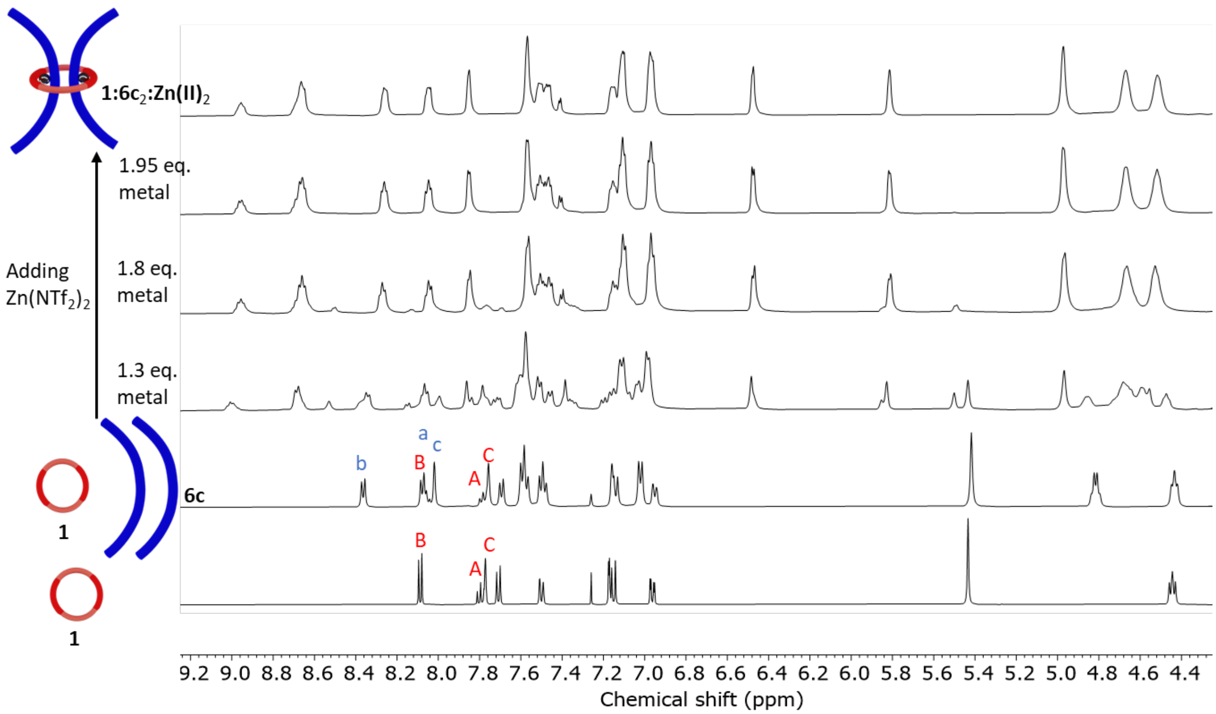

Figure S37. Partial ^{1}H-NMR overlay (500 MHz, 25°C, Solvent: increasing acetonitrile-d_3 in chloroform-d increasing upwards) of metal addition during NMR titrations.

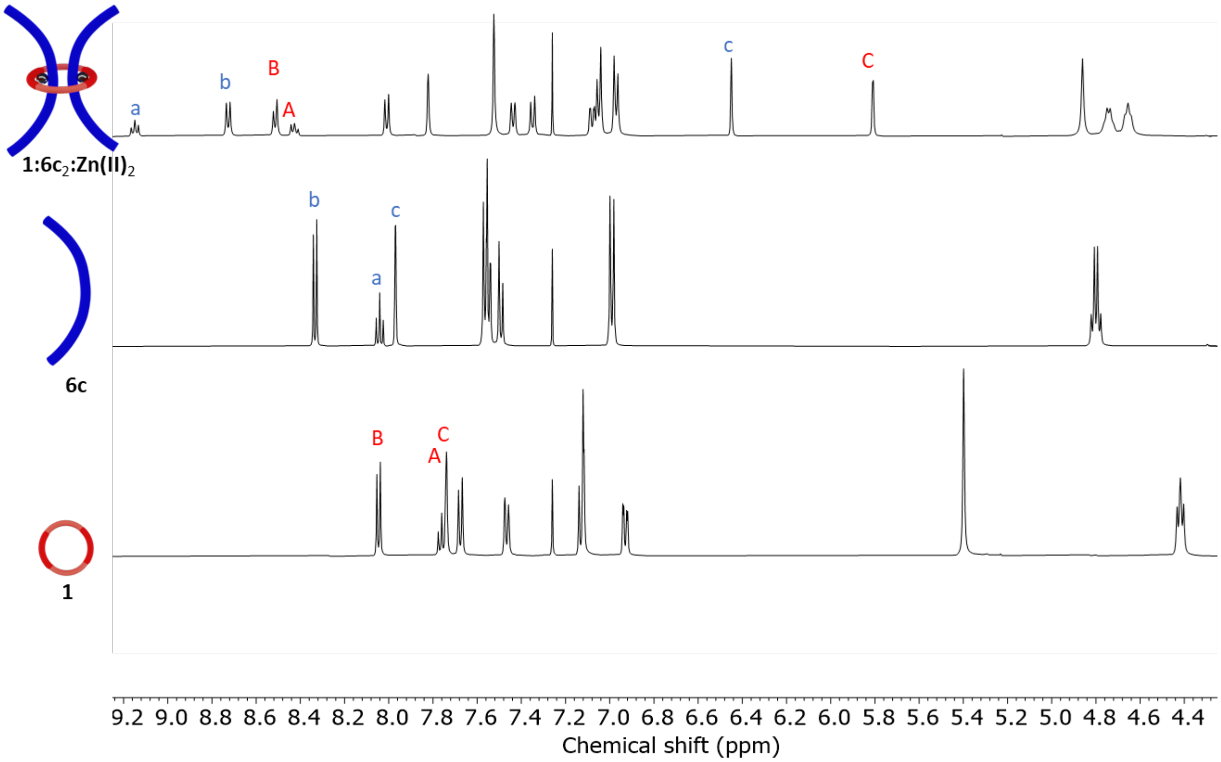

Figure S38. Partial ^{1}H-NMR overlay (500 MHz, 25°C) of 10 mM **1:6c$_2$:Zn(II)$_2$**, 20 mM **6c,** and 10 mM **1** (5% acetonitrile-d_3 in chloroform-d) after equilibration.

DOSY NMR of the macrocycle, threads, and Zn(II) assemblies

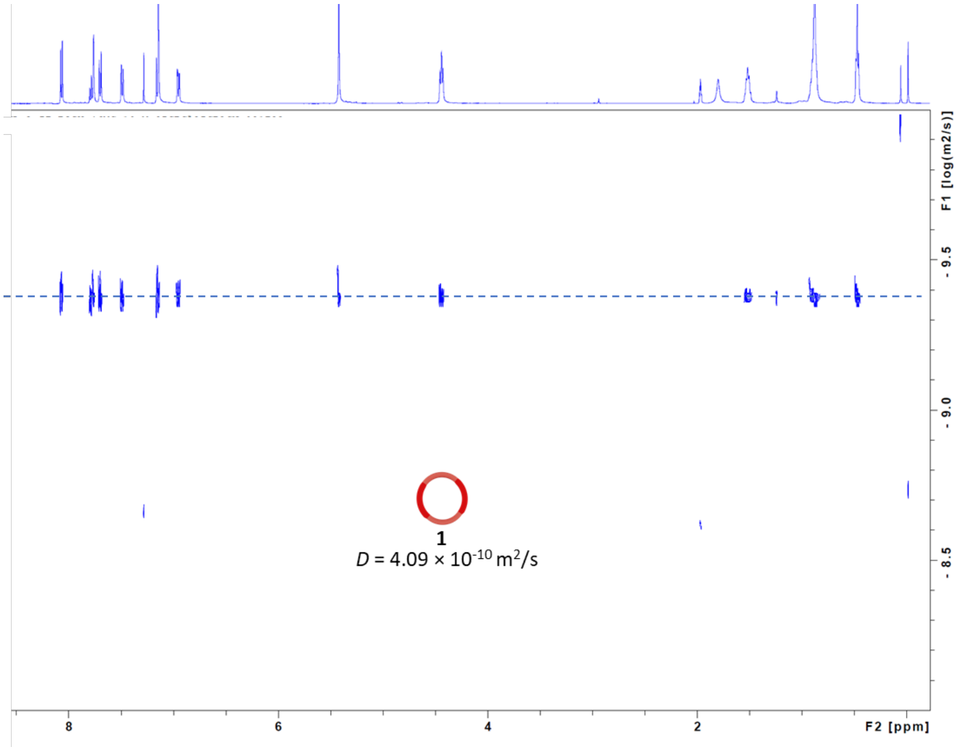

Figure S39. DOSY Spectrum (500 MHz, 25°C) of 10 mM **1** (5% acetonitrile-d_3 in chloroform-d).

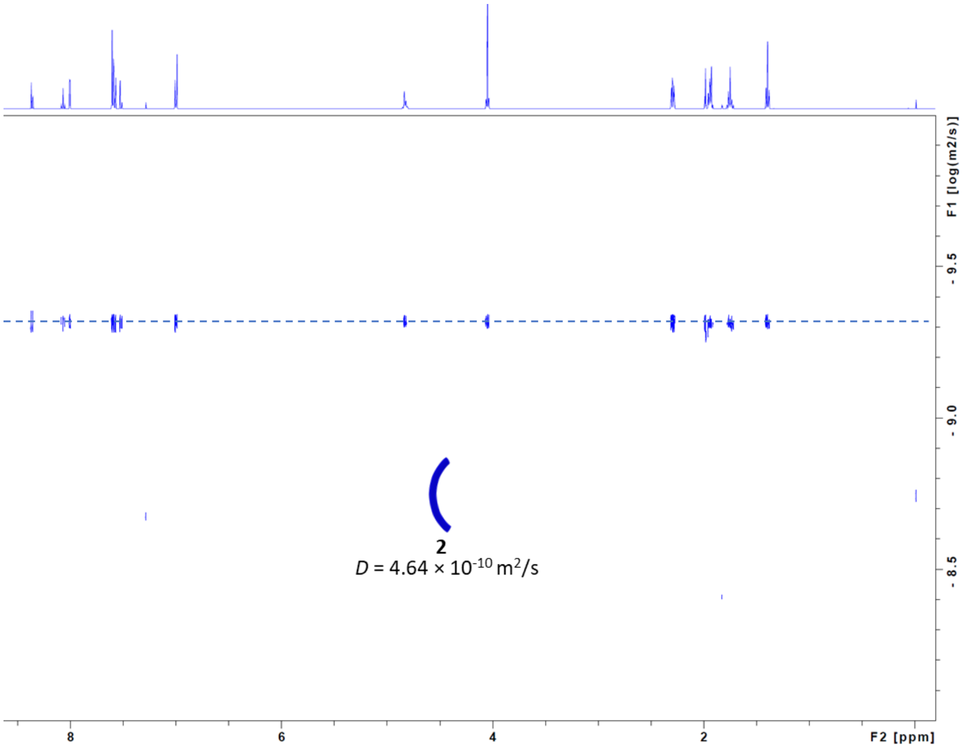

Figure S40. DOSY Spectrum (500 MHz, 25°C) of 20 mM **2** (5% acetonitrile-d_3 in chloroform-d).

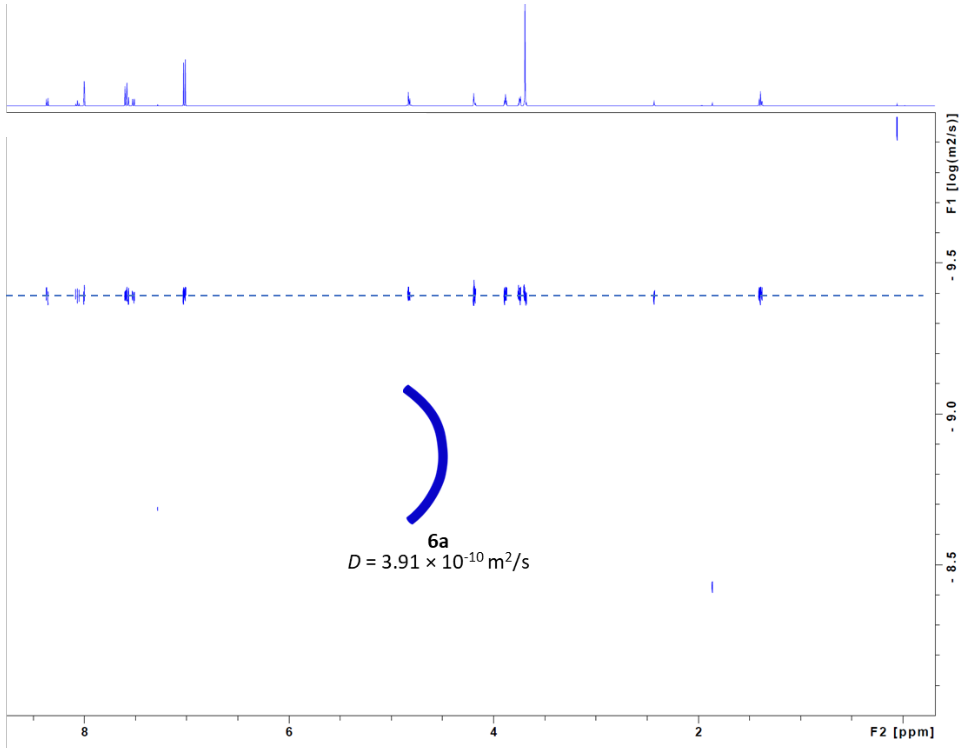

Figure S41. DOSY Spectrum (500 MHz, 25°C) of 20 mM **6a** (5% acetonitrile-d_3 in chloroform-*d*).

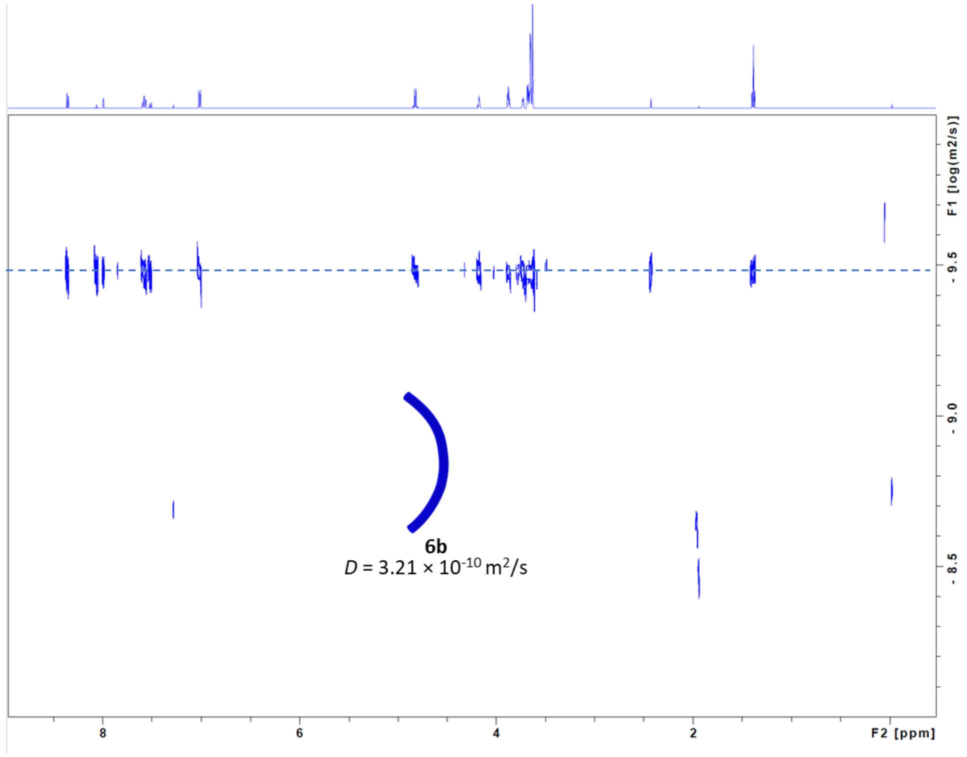

Figure S42. DOSY Spectrum (500 MHz, 25°C) of 20 mM **6b** (5% acetonitrile-d_3 in chloroform-*d*).

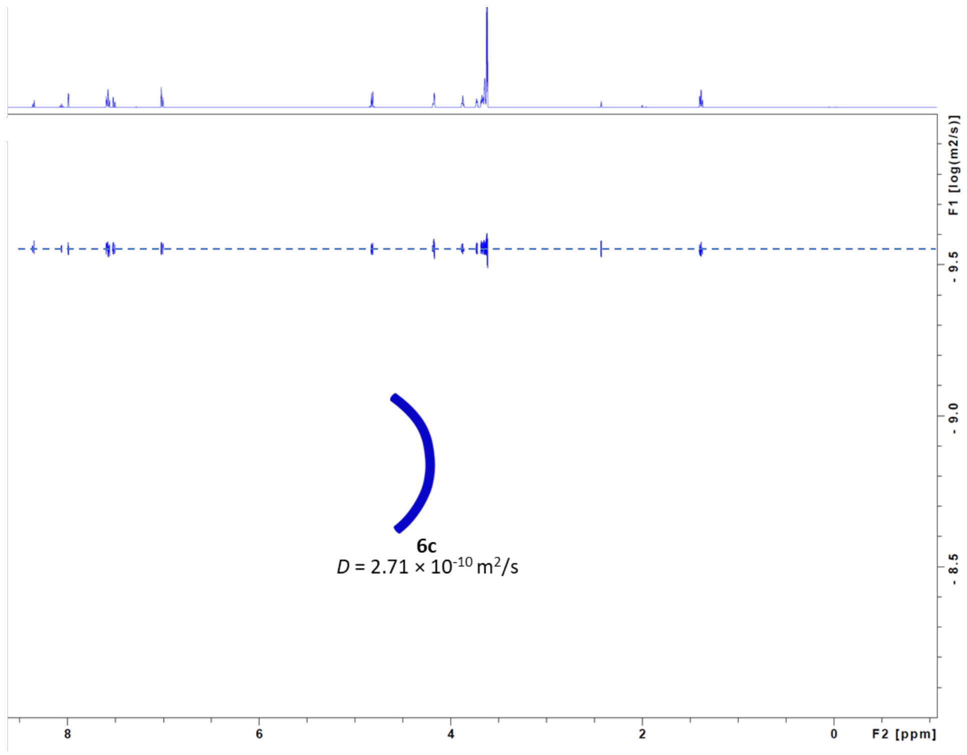

Figure S43. DOSY Spectrum (500 MHz, 25°C) of 20 mM **6c** (5% acetonitrile-d_3 in chloroform-d).

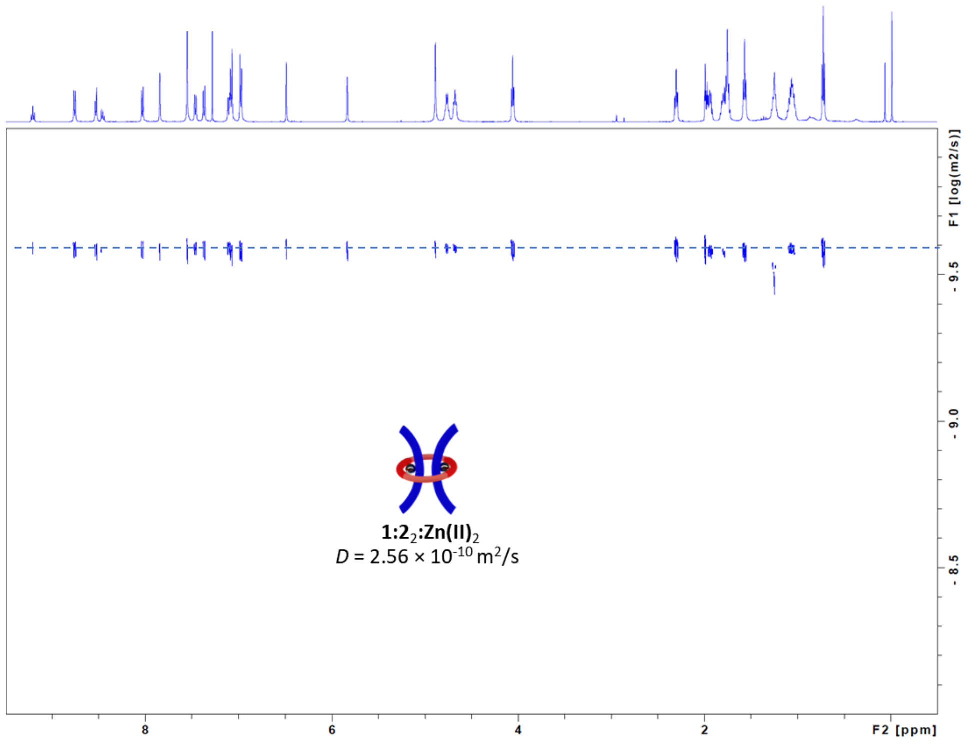

Figure S44. DOSY Spectrum (500 MHz, 25°C) of 10 mM **1:2$_2$:Zn(II)$_2$** (5% acetonitrile-d_3 in chloroform-d).

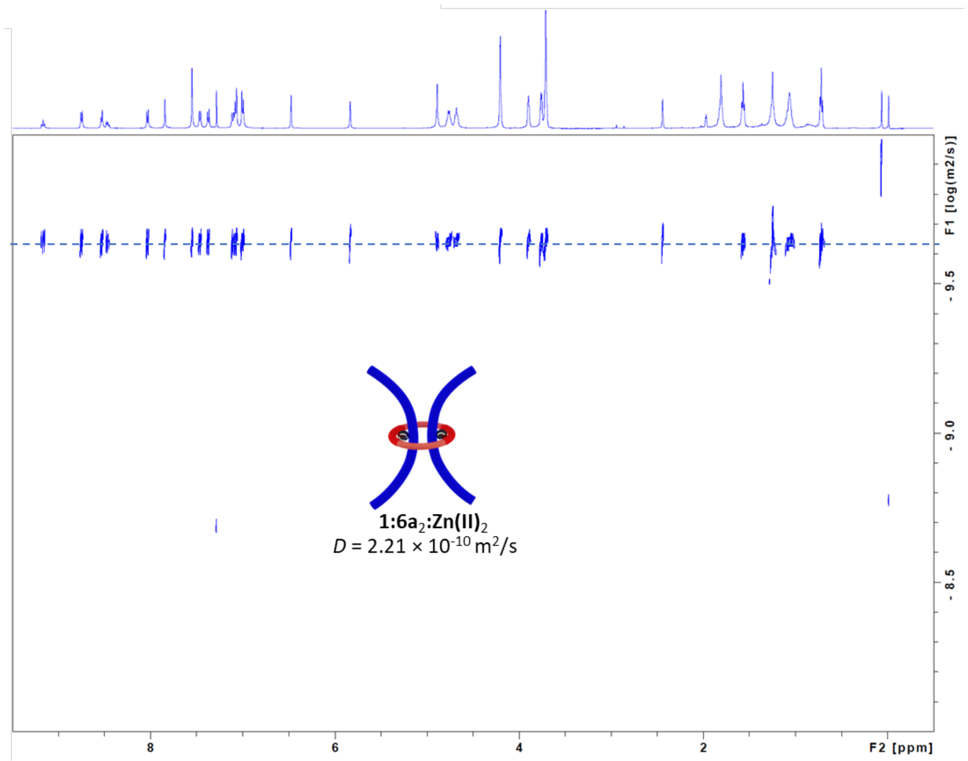

Figure S45. DOSY Spectrum (500 MHz, 25°C) of 10 mM **1:6a$_2$:Zn(II)$_2$** (5% acetonitrile-d_3 in chloroform-d).

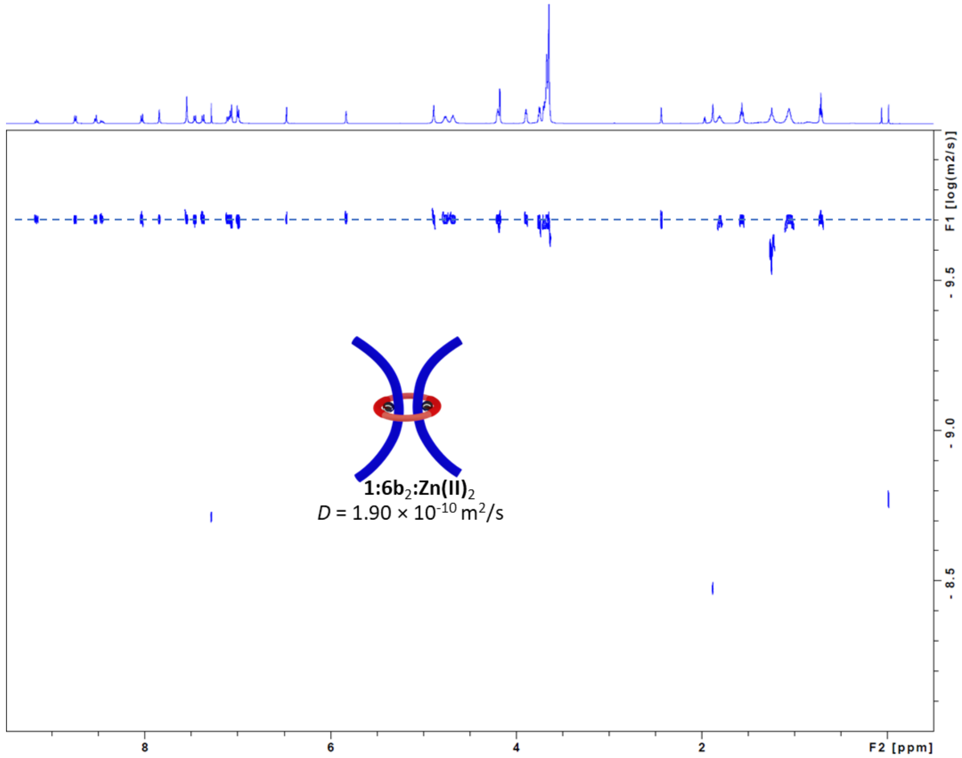

Figure S46. DOSY Spectrum (500 MHz, 25°C) of 10 mM **1:6b$_2$:Zn(II)$_2$** (5% acetonitrile-d_3 in chloroform-d).

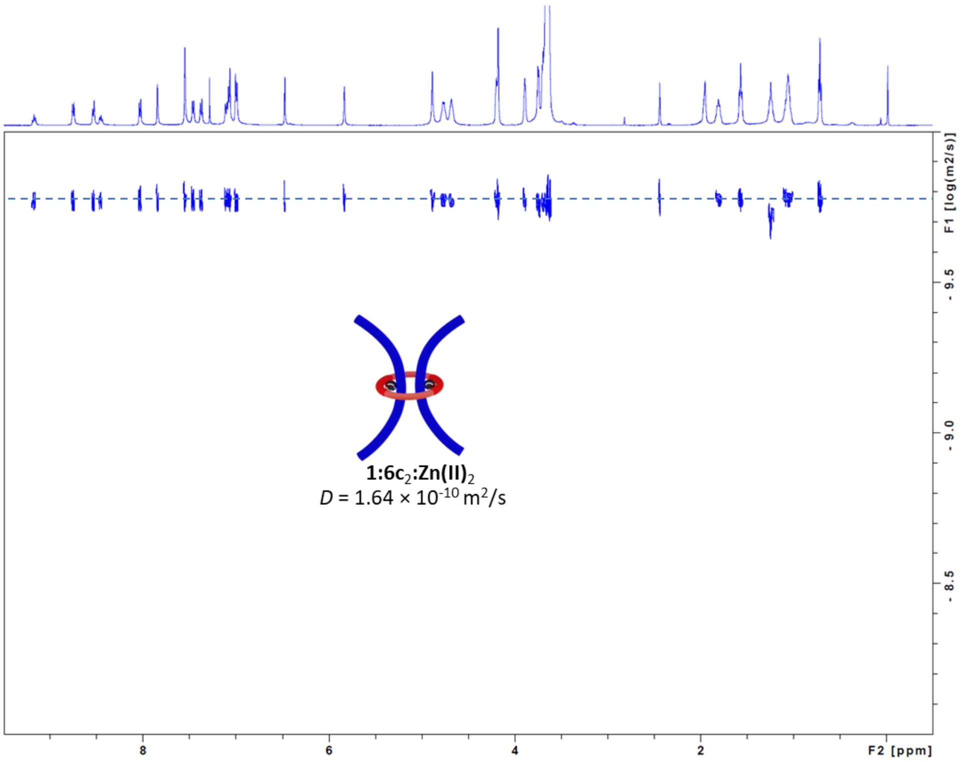

Figure S47. DOSY Spectrum (500 MHz, 25°C) of 10 mM $\mathbf{1{:}8_2{:}Zn(II)_2}$ (5% acetonitrile-d_3 in chloroform-d).

Table S1. Diffusion coefficient from DOSY experiments (500 MHz, 25°C, 5% acetonitrile-d_3 in chloroform-d)

	Diffusion coefficient (D, m^2/s)		Diffusion coefficient (D, m^2/s)
Thread **2**	4.64×10^{-10} m^2/s	Zn(II) Assembly of $\mathbf{1{:}2_2{:}Zn(II)_2}$	2.56×10^{-10} m^2/s
Thread **6a**	3.91×10^{-10} m^2/s	Zn(II) Assembly of $\mathbf{1{:}6a_2{:}Zn(II)_2}$	2.21×10^{-10} m^2/s
Thread **6b**	3.21×10^{-10} m^2/s	Zn(II) Assembly of $\mathbf{1{:}6b_2{:}Zn(II)_2}$	1.90×10^{-10} m^2/s
Thread **6c**	2.71×10^{-10} m^2/s	Zn(II) Assembly of $\mathbf{1{:}6c_2{:}Zn(II)_2}$	1.64×10^{-10} m^2/s
Macrocycle **1**	4.09×10^{-10} m^2/s		

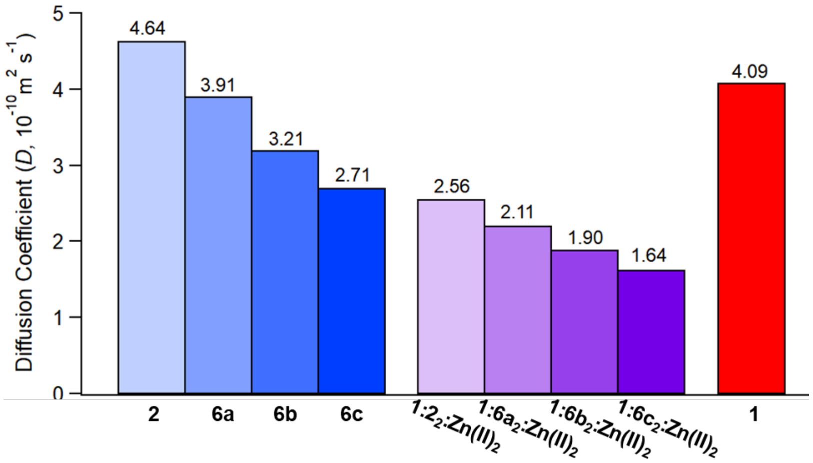

Figure S48. Diffusion coefficients from DOSY spectra (500 MHz, 25°C) of all threads, macrocycle, and their self-assemblies (5% acetonitrile-d_3 in chloroform-d).

Prepolymerization for **1:6a₂:Zn(II)₂** with **3b**

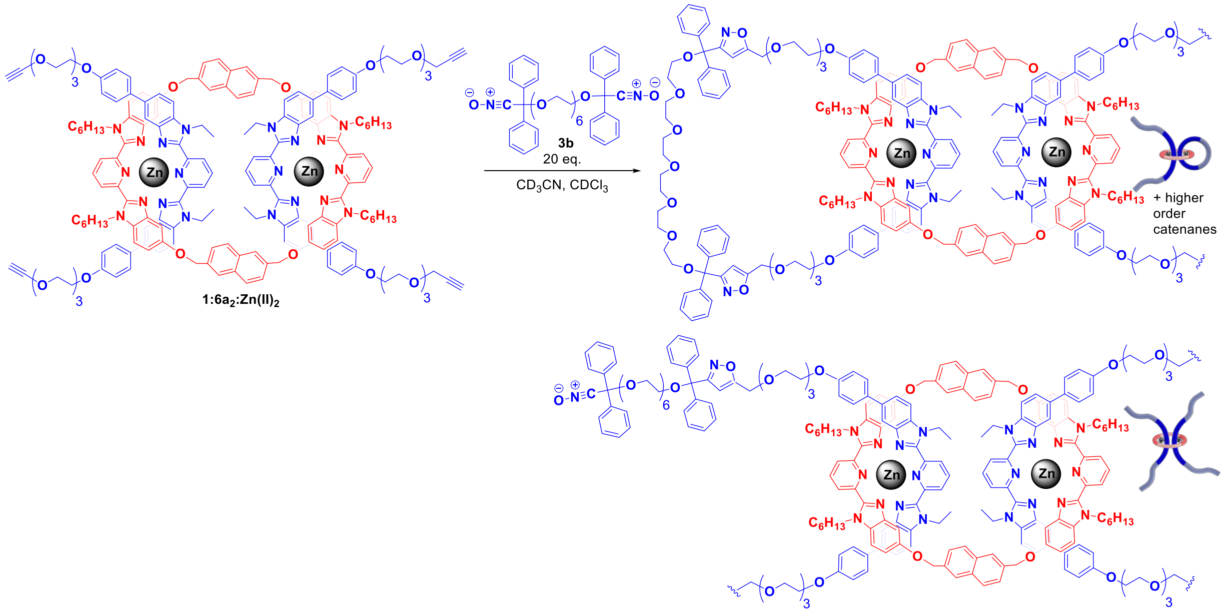

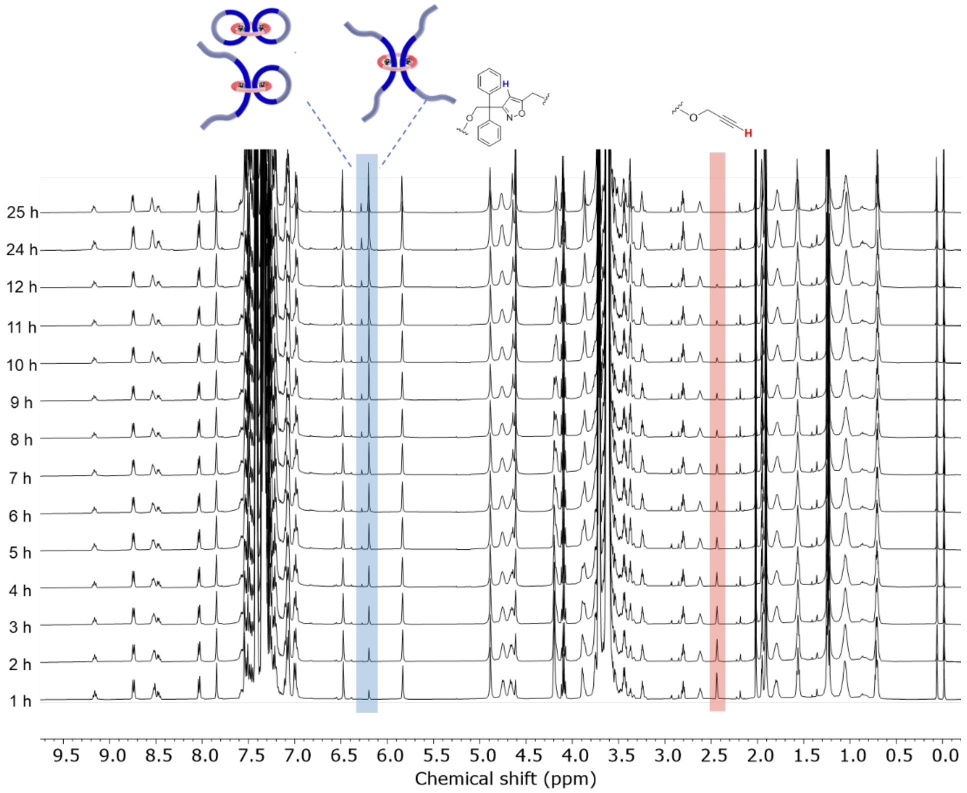

Figure S49. Prepolymerization of **1:6a$_2$:Zn(II)$_2$** with an excess amount of monomer **3b** (20 equivalents) in 5% acetonitrile-*d*$_3$ in chloroform-*d* to determine the reaction kinetics and the amount of backbiting. ^{1}H-NMR overlay (500 MHz, 25°C) of prepolymerization of **1:6a$_2$:Zn(II)$_2$** with an excess amount of monomer **3b** (20 equivalents) at different reaction times (1 h to 25 h). The disappearance of the signal that corresponds to the terminal protons of the alkyne (2.44 ppm) shows that **1:6a$_2$:Zn(II)$_2$** has been fully reacted. The increase of the signal that corresponds to the protons of the isoxazole (6.20 ppm for linear, and 6.28 ppm for catenane) shows the 15% backbiting.

Monomer **3b** (25.0 mg, 0.0359 mmol) and **1:6a$_2$:Zn(II)$_2$** (7.7 mg, 0.00177 mmol) were dissolved in 5% acetonitrile-*d*$_3$ in chloroform-*d* (0.5 mL) and added to an NMR tube. The NMR tube was constantly shaken by hand to allow thorough mixing.

Prepolymerization for **1:6b$_2$:Zn(II)$_2$** with **3b**

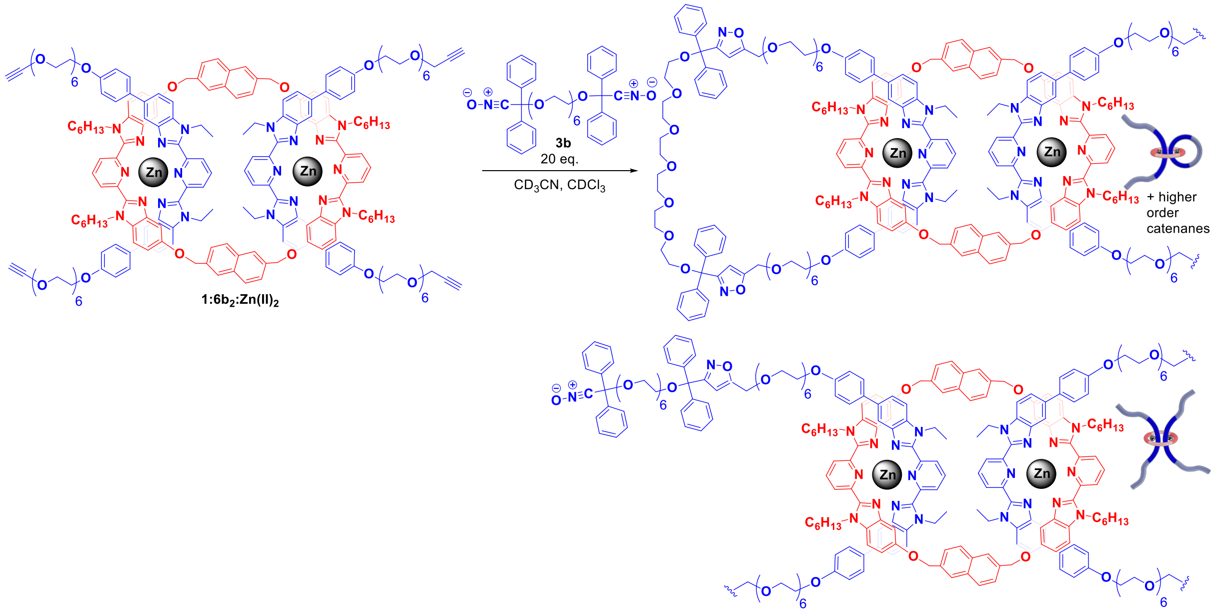

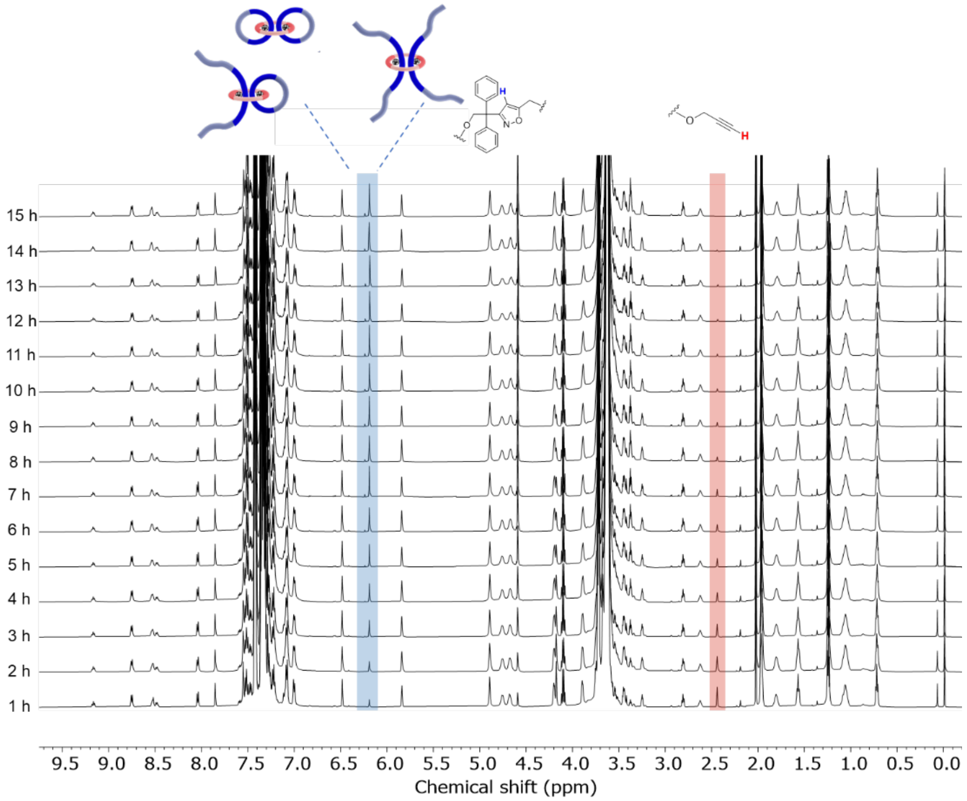

Figure S50. Prepolymerization of **1:6b$_2$:Zn(II)$_2$** with an excess amount of monomer **3b** (20 equivalents) in 5% acetonitrile-d_3 in chloroform-*d* to determine the reaction kinetics and the amount of backbiting. ^{1}H-NMR overlay (500 MHz, 25°C) of prepolymerization of **1:6b$_2$:Zn(II)$_2$** with an excess amount of monomer **3b** (20 equivalents) at different reaction times (1 h to 15 h). The disappearance of the signal that corresponds to the terminal protons of the alkyne (2.44 ppm) shows that **1:6b$_2$:Zn(II)$_2$** has been fully reacted. The increase of the signal that corresponds to the protons of the isoxazole (6.20 ppm for linear, and 6.25 ppm for catenane) shows the 7% backbiting.

Monomer **3b** (25.0 mg, 0.0359 mmol) and **1:6b$_2$:Zn(II)$_2$** (8.7 mg, 0.00177 mmol) were dissolved in 5% acetonitrile-d_3 in chloroform-*d* (0.5 mL) and added to an NMR tube. The NMR tube was constantly shaken by hand to allow thorough mixing.

Prepolymerization for **1:6c₂:Zn(II)₂** with **3b**

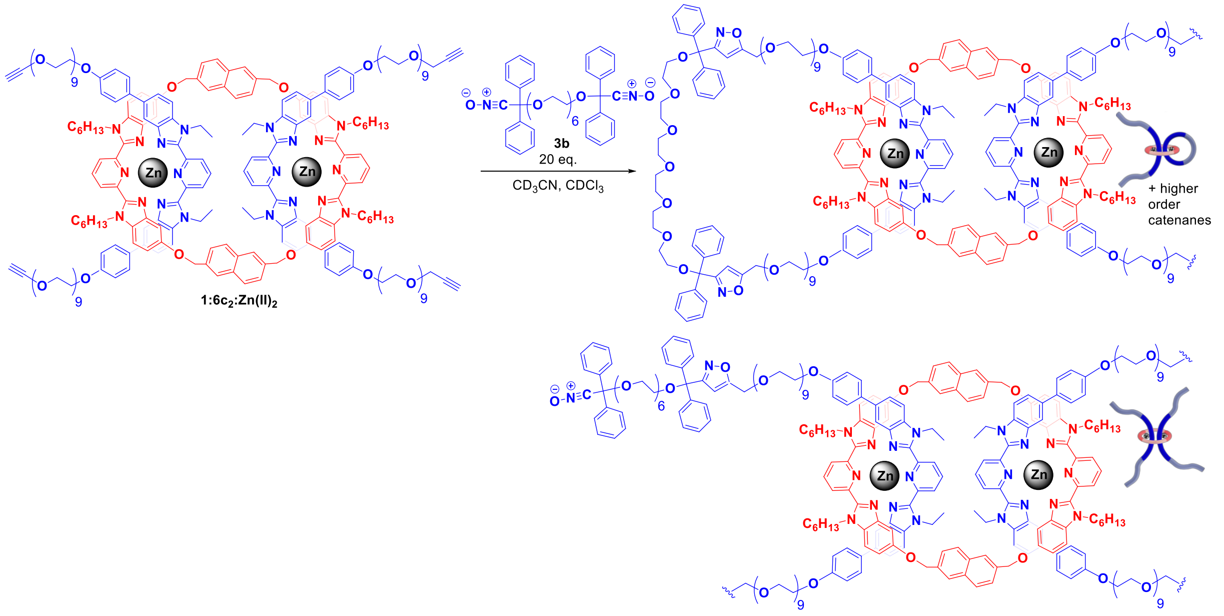

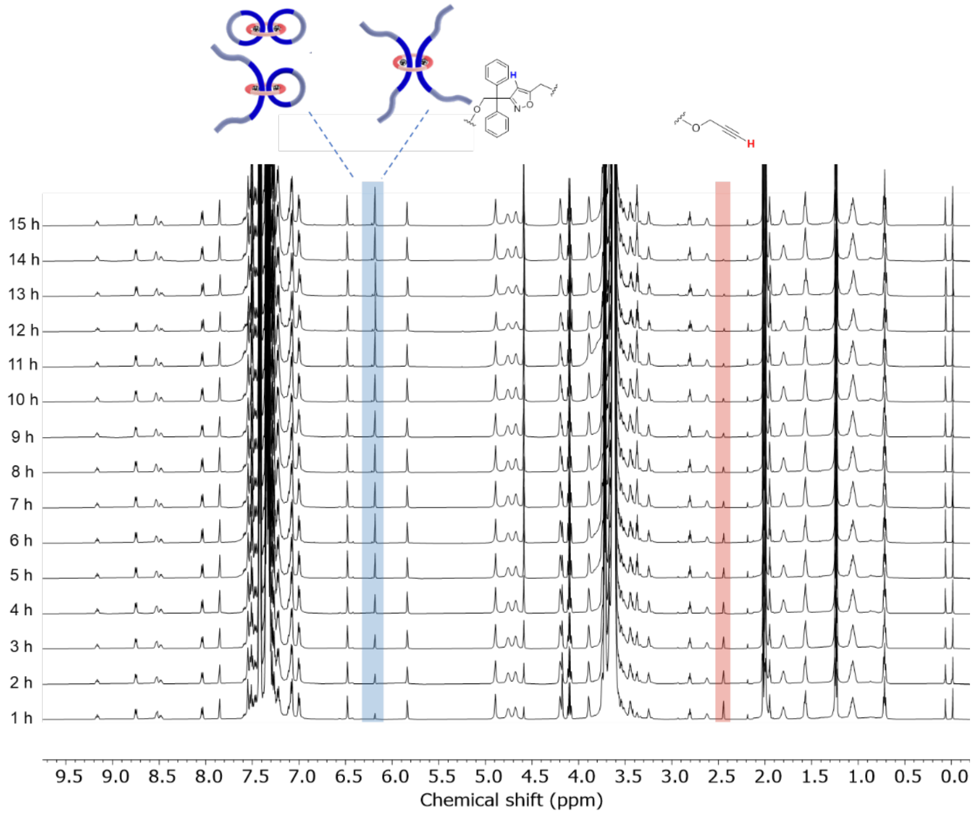

Figure S51. Prepolymerization of **1:6c$_2$:Zn(II)$_2$** with an excess amount of monomer **3b** (20 equivalents) in 5% acetonitrile-*d*$_3$ in chloroform-*d* to determine the reaction kinetics and the amount of backbiting. ^{1}H-NMR overlay (500 MHz, 25°C) of prepolymerization of **1:6c$_2$:Zn(II)$_2$** with an excess amount of monomer **3b** (20 equivalents) at different reaction times (1 h to 15 h). The disappearance of the signal that corresponds to the terminal protons of the alkyne (2.44 ppm) shows that **1:6c$_2$:Zn(II)$_2$** has been fully reacted. The increase of the signal that corresponds to the protons of the isoxazole (6.20 ppm for linear, and 6.22 ppm for catenane) shows the 5% backbiting.

Monomer **3b** (25.0 mg, 0.0359 mmol) and **1:6c$_2$:Zn(II)$_2$** (9.6 mg, 0.00177 mmol) were dissolved in 5% acetonitrile-*d*$_3$ in chloroform-*d* (0.5 mL) and added to an NMR tube. The NMR tube was constantly shaken by hand to allow thorough mixing.

Catenane reaction for **1:6c$_2$:Zn(II)$_2$** with **3b**

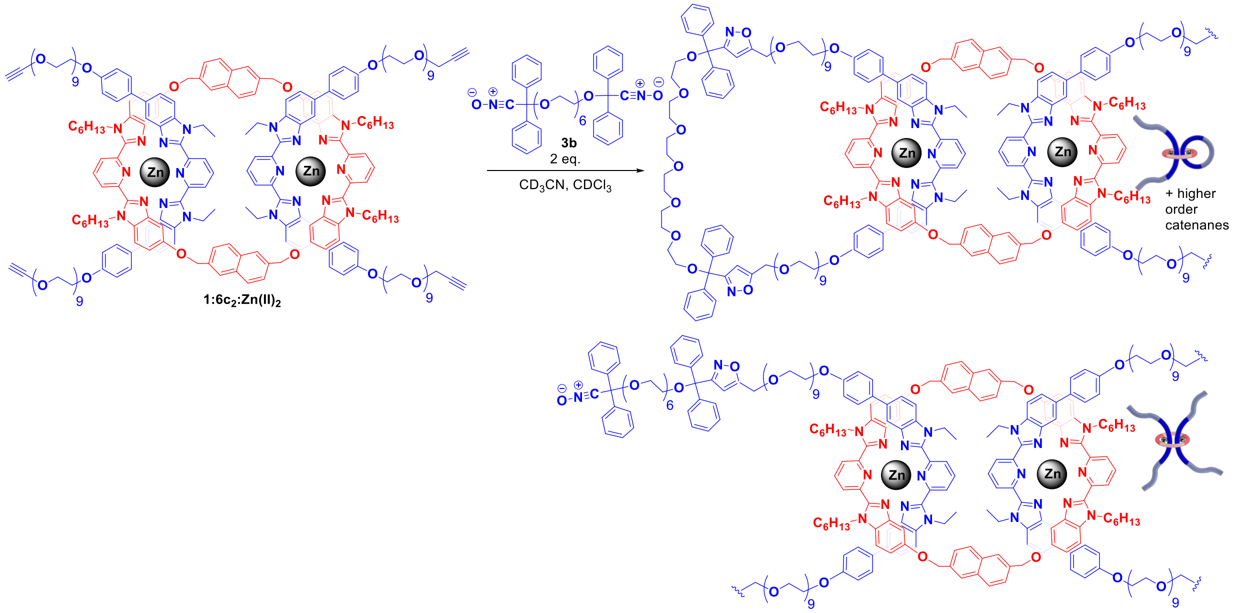

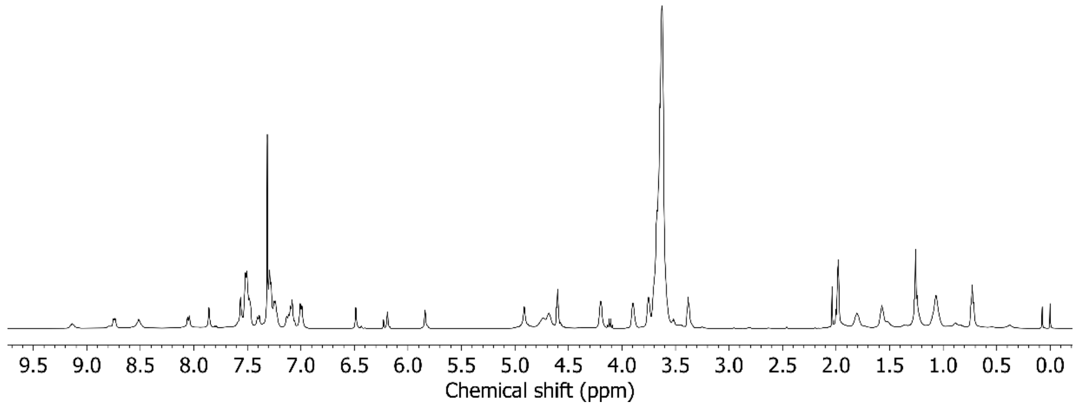

Figure S52. Cyclization of **1:6c$_2$:Zn(II)$_2$** with two equivalents of monomer **3b** in 5% acetonitrile-*d*$_3$ in chloroform-*d* to target the catenane. Crude ^{1}H-NMR (500 MHz, 25°C) of cyclization of **1:6c$_2$:Zn(II)$_2$** with two equivalents of monomer **3b** in 5% acetonitrile-*d*$_3$ in chloroform-*d* shows an increase in the protons of the isoxazole in the catenane and a decrease in the rotaxane.

Monomer **3b** (4.65 mg, 0.00668 mmol) and **1:6c$_2$:Zn(II)$_2$** (18.1 mg, 0.00334 mmol) were dissolved in 5% acetonitrile-*d*$_3$ in chloroform-*d* (0.67 mL) and added to an NMR tube. The mixture was heated to 45 °C for 8 d. The crude NMR was taken. The solvent was removed in vacuo. The residue was dissolved in DCM (15 mL), and acetonitrile (1 mL) was added. To demetalate, tetrabutylammonium hydroxide solution (1M in methanol, 100 µL) was added dropwise to demetalate for 30 min. The reaction mixture was then diluted with DCM (10 mL) and washed with water (5 × 20 mL). The organic layer was separated, and the solvent was removed in vacuo.

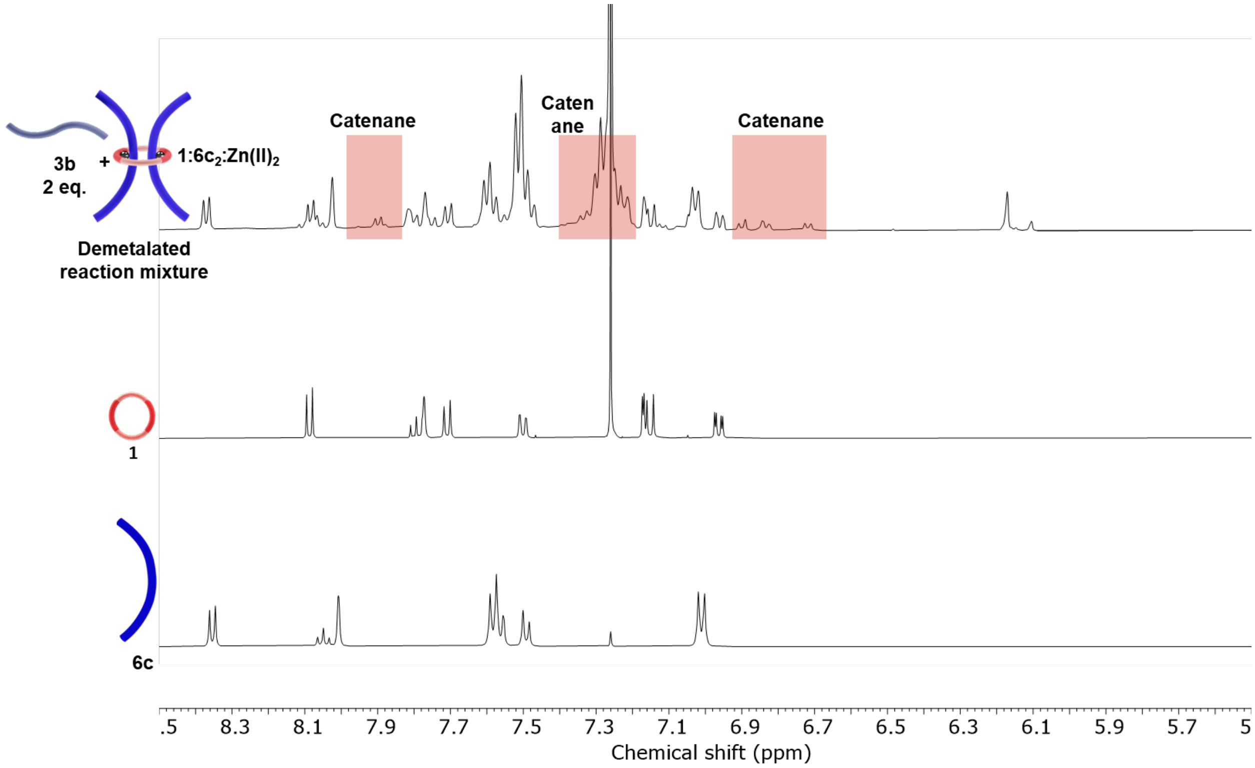

Figure S53. Partial ^{1}H-NMR overlay (500 MHz, $CDCl_3$, 25°C) of **1**, **6c**, and the reaction mixture from Figure S52 after demetalation.

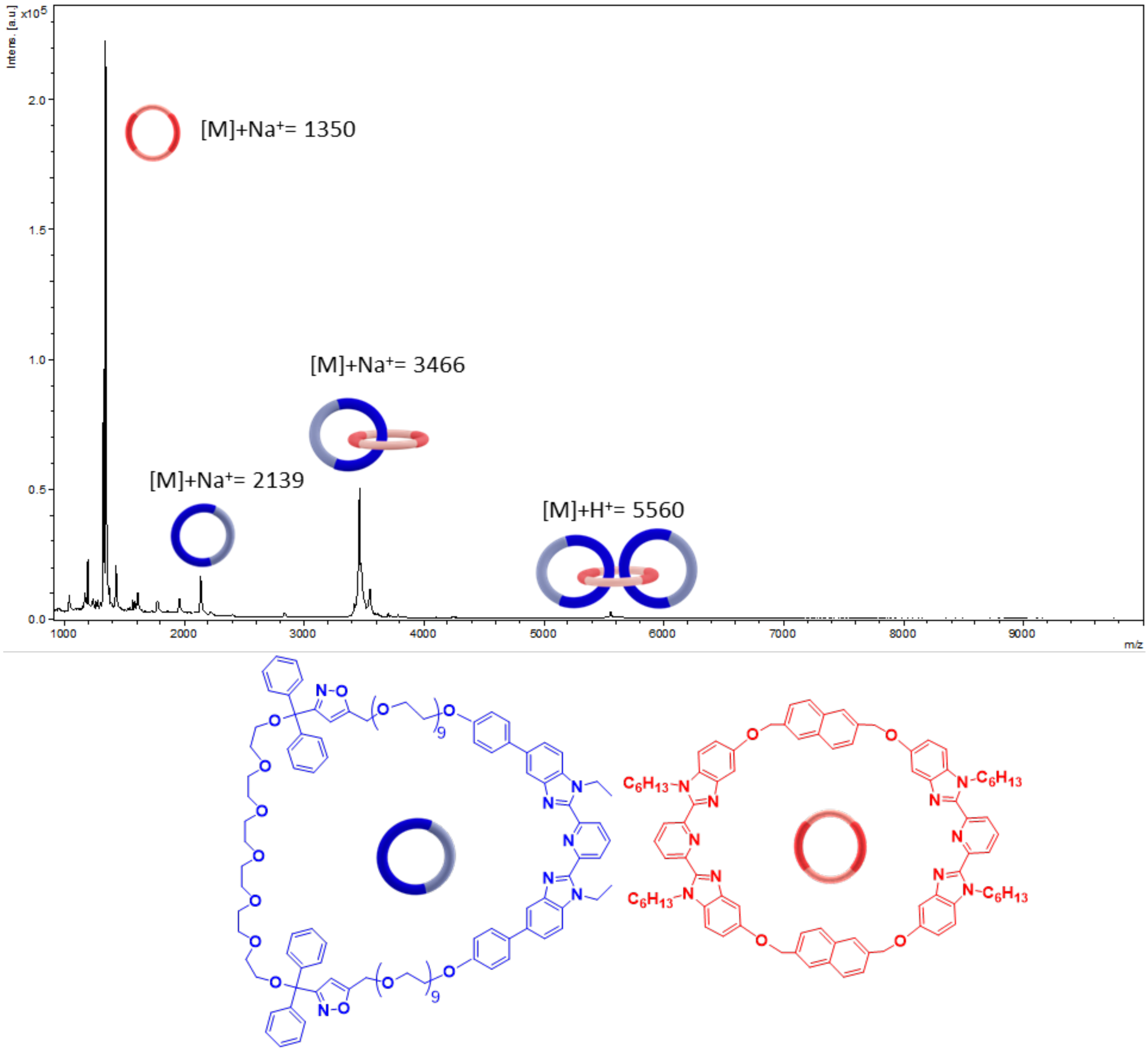

Figure S54. MALDI-TOF mass spectrometry data for the reaction mixture from Figure S52 after demetalation. The chemical structures for products detected are shown below.

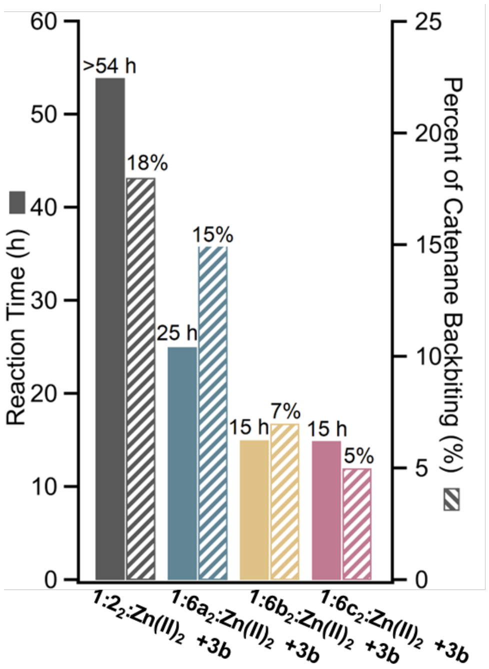

Figure S55. Bar graph to summarize the reaction time for different P3Rs and the percent of catenane product in the P3Rs when reacting with an excess amount of **3b** (20 equivalents). When increasing the length of the thread in the P3Rs, the reaction time shortens from over 54 h for **1:2$_2$:Zn(II)$_2$** to 15 h for **1:6b$_2$:Zn(II)$_2$** and **1:6c$_2$:Zn(II)$_2$,** and the catenane backbiting decreases from 18% for **1:2$_2$:Zn(II)$_2$** to 5% for **1:6c$_2$:Zn(II)$_2$.**

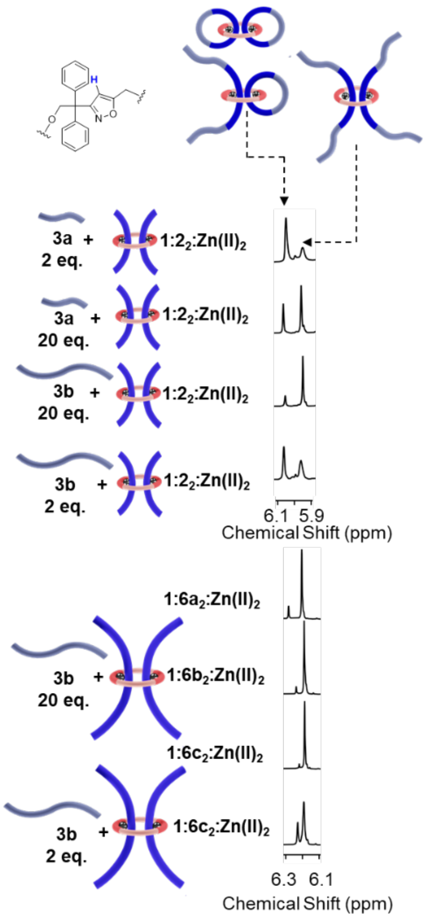

Figure S56. Crude ^{1}H-NMR (500 MHz, 25°C) spectra focusing on the isoxazole peaks in the catenane and rotaxane products in reactions of nitrile-oxide monomers and P3Rs.

Doubly-Threaded Slide-Ring Polycatenane Network (SR-PCN) $7_{80/20}$ and $8_{a/b}$ Synthesis

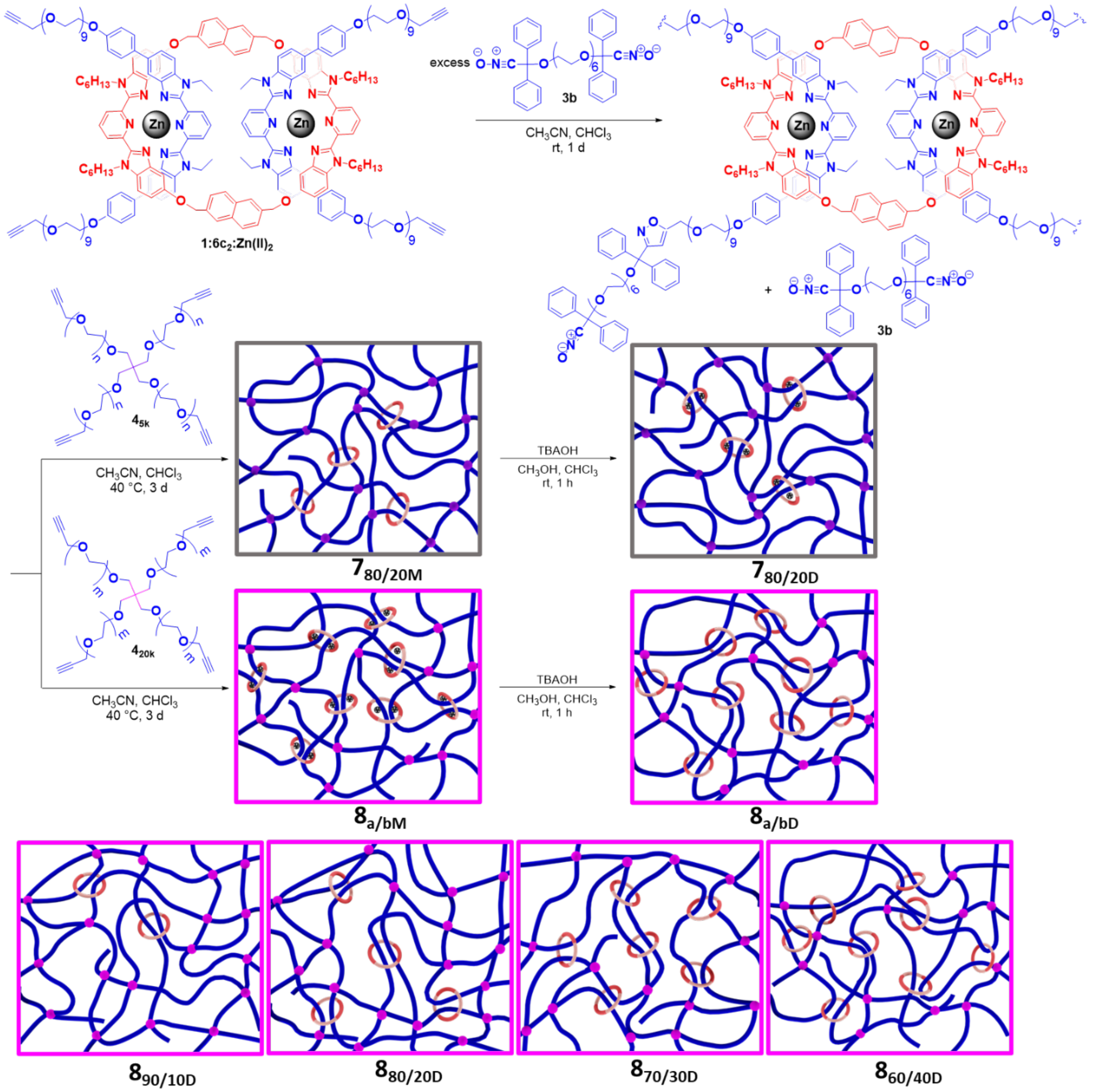

Figure S57. Synthesis of doubly-threaded SR-PCN $\mathbf{7_{80/20}}$ and $\mathbf{8_{a/b}}$ series. Polymerization of doubly-threaded SR-PCN $\mathbf{7_{80/20M}}$ with **1:6c$_2$:Zn(II)$_2$** (*20* mol%) and $\mathbf{4_{5k}}$ (*80* mol%), and demetalation with a dilute solution of tetrabutylammonium hydroxide (TBAOH) to $\mathbf{7_{80/20D}}$. SR-PCN $\mathbf{8_{a/bM}}$ series with varying amounts of **1:6c$_2$:Zn(II)$_2$** (n_{P3R} mol, *b* mol%) and $\mathbf{4_{20k}}$ (m_{PEG} mol, *a* mol%), and demetalation with a dilute solution of tetrabutylammonium hydroxide (TBAOH) to $\mathbf{8_{a/bD}}$.

Monomer **3b** (12.5 mg, 0.0179 mmol) was added to a 2 dram glass shell vial with plug style closures. A stock solution (250 μL) of **1:6c$_2$:Zn(II)$_2$** (n_{P3R} mol, see Table S2) in 30% acetonitrile in chloroform was added. The vial was capped and sealed with parafilm and electrical tape. The reaction mixture was stirred for 24 h. Then, 4-arm PEG-alkyne $\mathbf{4_{20k}}$ (M_n = 20.0 kgmol^{-1}, m_{PEG} mol, see Table S2) was added. 100 μL of 30% acetonitrile in chloroform (containing 1.7 mg of TMS silica for DLS microrheology measurements) was added. The reaction mixture was stirred for 2 min, and the stir bar was removed. The vial was capped and sealed with parafilm and electrical tape and left to gel at 40°C for 72

h. A spatula was used to scoop out the crude metalated doubly-threaded SR-PCN (**8**$_{a/bM}$) and transfer to a Teflon dish to dry in a fume hood overnight. Then, the crude **8**$_{a/bM}$ was dried in vacuo (48 h). The mass of the crude, dried **8**$_{a/bM}$ was recorded. To remove low molecular weight components from the crude **8**$_{a/bM}$ and calculate the gel fraction (GF), the crude, dried **8**$_{a/bM}$ was added in a beaker. Chloroform (500 mL) was added. The solution was heated to 55°C on a hot plate for 4 h. A watch glass was added on top of the beaker to mitigate evaporation. The swollen, washed **8**$_{a/bM}$ was transferred to a Teflon dish to dry in a fume hood overnight. Then, the washed **8**$_{a/bM}$ was dried in vacuo (48 h) at 50 °C. The mass of the dried, washed **8**$_{a/bM}$ was recorded.

7$_{80/20M}$ was synthesized and washed in the same way as **8**$_{a/bM}$ with monomer **3b** (12.5 mg, 1.8E-05 mol), a stock solution (250 µL, 39.2 mg/mL) of **1**:**6c**$_2$**:Zn(II)**$_2$ (9.8 mg, 1.8E-06 mol) in 30% acetonitrile in chloroform, and 4-arm PEG-alkyne **4**$_{5k}$ (M_n = 5.0 kgmol^{-1}, 36.0 mg).

The gel fraction (GF) of **8**$_{a/bM}$ and **7**$_{80/20M}$ was calculated from Equation S1:

$$\text{GF (wt \%)} = \frac{m_{dried,washed,metalated}}{m_{crude,dried,metalated}} \text{ x } 100 \qquad \text{(S1)}$$

Where $m_{crude,dried,metalated}$ is the weight of the crude, dried **8**$_{a/bM}$ and **7**$_{80/20M}$ after gelation, and $m_{dried,washed,metalated}$ is the weight of the dried, washed **8**$_{a/bM}$ and **7**$_{80/20M}$ after washing.

The washed **8**$_{a/bM}$ (Figure S58) and **7**$_{80/20M}$ both exhibited a yellow color under ambient light and a yellow fluorescence under 365 nm UV light on account of the Bip$_2$/Zn(II) complexes and qualitatively confirms the successful incorporation of **1:6c**$_2$**:Zn(II)**$_2$ moieties into the network.

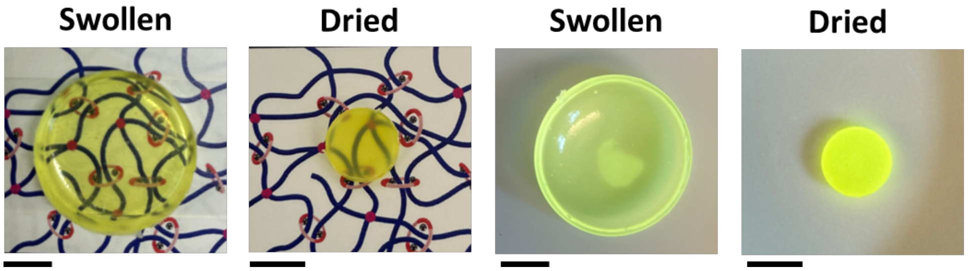

Figure S58. Pictures of **8**$_{a/bM}$ under ambient light (left) and under 365 nm UV light (right), 1 cm scale bar. **8**$_{80/20M}$ pictured.

<u>Demetalation and Washing</u>

To demetalate, the dried, washed **8**$_{a/bM}$ was added to a Teflon dish. 35 mL of chloroform and 5 mL of methanol were added to swell the network, and then tetrabutylammonium hydroxide solution (1M in methanol, 50 µL) was added dropwise to demetalate for 1 h, resulting in a slow color change from yellow to light blue under 365 nm UV light, the fluorescence color of the free ligand, consistent with the decomplexation/demetalation of the gels. This process was repeated three times. Then, the swollen demetalated doubly-threaded SR-PCN (**8**$_{a/bD}$) was transferred to a second Teflon dish. 20 mL chloroform and 20 mL methanol were added to soak the network for 1 h. This process was repeated for six times. Then, swollen **8**$_{a/bD}$ was added in a beaker. Chloroform (500 mL) was added. The solution was heated to 55°C on a hot plate for 4 h. A watch glass was added on top of the beaker to mitigate evaporation. The swollen, washed **8**$_{a/bD}$ was transferred to a Teflon dish to dry in a fume hood overnight. Then, washed **8**$_{a/bD}$ was dried in vacuo (48 h) at 50 °C. The mass of the dried, washed **8**$_{a/bD}$ was recorded. The soluble fractions (from the 500 mL chloroform wash for the metalated network, the 35 mL of chloroform and 5 mL of methanol with tetrabutylammonium hydroxide (× 3) during demetalation, the 20 mL chloroform and 20 mL methanol (× 6) demetalation washes, and the 500 mL chloroform demetalation wash) were combined, and the solvent was removed in vacuo. The residual was dissolved in DCM (10 mL) and washed with water (30 mL × 5). The solvent was removed in vacuo, and the residual was analyzed by ^{1}H-NMR analysis. **7**$_{80/20M}$ was demetalated and washed to **7**$_{80/20D}$ in the same way as **8**$_{a/bM}$.

The gel fraction (GF) of **8**$_{a/bD}$ and **7**$_{80/20D}$ was calculated from Equation S2:

$$\text{GF (wt \%)} = \frac{m_{dried,washed,demetalated}}{m_{crude,dried,metalated} - m_{zn(ntf2)2}} \text{ x } 100 \qquad \text{(S2)}$$

Where $m_{crude,dried,metalated}$ is the weight of the crude, dried **8**$_{a/bM}$ and **7**$_{80/20M}$ after gelation, $m_{dried,washed,demetalated}$ is the weight of the dried, washed **8**$_{a/bD}$ and **7**$_{80/20D}$ after washing, and $m_{zn(ntf2)2}$ is the weight of zinc di[bis(trifluoromethylsulfonyl)imide] from **1:6c$_2$:Zn(II)$_2$** to account for the loss of metal and counterion masses during demetalation.

The washed **8**$_{a/bD}$ (Figure S59) and **7**$_{80/20D}$ both exhibited a blue fluorescence under 365 nm UV light on account of the Bip ligands and qualitatively confirms the successful incorporation of Bip moieties into the network.

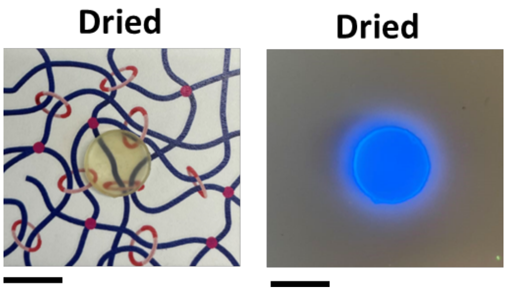

Figure S59. Pictures of **8**$_{a/bD}$ under ambient light (left) and under 365 nm UV light (right), 1 cm scale bar. **8**$_{80/20D}$ pictured.

Table S2. Composition of **doubly-threaded SR-PCN 8**$_{a/bM}$ series

Doubly-threaded SR-PCN	Monomer **3b**	4-arm PEG-Alkyne **4**$_{20k}$, m_{PEG} mol	**1:6c$_2$:Zn(II)$_2$**, n_{P3R} mol	**1:6c$_2$:Zn(II)$_2$**, Stock solution concentration
**8**$_{90/10M}$	12.5 mg, 1.8E-05 mol	162 mg, 8.1E-06 mol	4.9 mg, 0.9E-06 mol	19.6 mg/mL
**8**$_{80/20M}$	12.5 mg, 1.8E-05 mol	144 mg, 7.2E-06 mol	9.8 mg, 1.8E-06 mol	39.2 mg/mL
**8**$_{70/30M}$	12.5 mg, 1.8E-05 mol	126 mg, 6.3E-06 mol	14.7 mg, 2.7E-06 mol	58.8 mg/mL
**8**$_{60/40M}$	12.5 mg, 1.8E-05 mol	108 mg, 5.4E-06 mol	19.6 mg, 3.6E-06 mol	78.4 mg/mL

Gel Fraction (GF) for **8**$_{a/bM}$

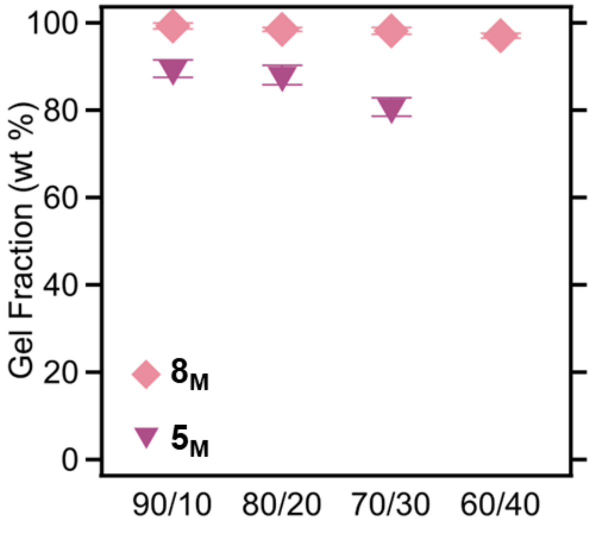

Figure S60. Gel fractions (GFs) for **8**$_{a/bM}$ calculated from Equation S1 compared with **5**$_{a/bM}$.

NMR analysis on the soluble fractions

It is important to calculate the number of rings and threads incorporated within the washed SR-PCN **7**$_{\mathbf{80/20D}}$ and **8**$_{\mathbf{a/bD}}$ and tangled networks **9**$_{\mathbf{a/bD}}$ series after the chloroform wash for the metalated network and the demetalation wash. Using the external standard NMR standard, the ring content can be calculated by comparing the molar ratio of a reference molecule (thymol), with a known structure and mass, to the macrocycle (MC) and thread in the combined soluble fractions. The relationship between the number (moles) of MC and thread in the combined soluble fraction and thymol is related to the integral of the diagnostic proton peak and the number of protons contributing to the peak, Equation S3:

$$n_{ring\ or\ thread\ extracted} = \frac{I_{MC\ or\ thread}}{I_{thymol}} \times \frac{N_{thymol}}{N_{MC\ or\ thread}} \times X_{thymol} \tag{S3}$$

where $n_{ring\ or\ thread\ extracted}$ is the number of moles of MC or thread in the combined soluble fraction, X_{thymol} is the number of moles of thymol (known), I is the integrated area of the diagnostic proton peak, and N is the number of protons contributing to the peak. X_{thymol} is known; the integrals are measured via ^{1}H-NMR; N corresponding to those integrals are known. The doublet on the macrocycle pyridine ring (N=4) at 8.08 ppm and the thread pyridine ring (N=2) were used as the diagnostic signal for the combined soluble fractions. Thymol was chosen for its diagnostic singlet (N=1) at 6.58 ppm that does not overlap with any other signals in the combined soluble fractions, resulting in the calculation of $n_{ring\ or\ thread\ extracted}$. Additionally, ^{1}H T1 measurements were conducted for the doublet on the macrocycle pyridine ring (N=4) at 8.08 ppm and the thread pyridine ring (N=2) and the singlet on the thymol standard (N=1) at 6.58 ppm before comparing the integral of these two peaks. The T1 value of the doublet on the macrocycle pyridine ring (N=4) at 8.08 ppm and the thread pyridine ring (N=2) and singlet on the thymol standard (N=1) at 6.58 ppm was observed to be lower than D1/4 when D1 = 20 sec. Consequently, the integral of these peaks could be reliably compared with an accuracy exceeding 95 %.

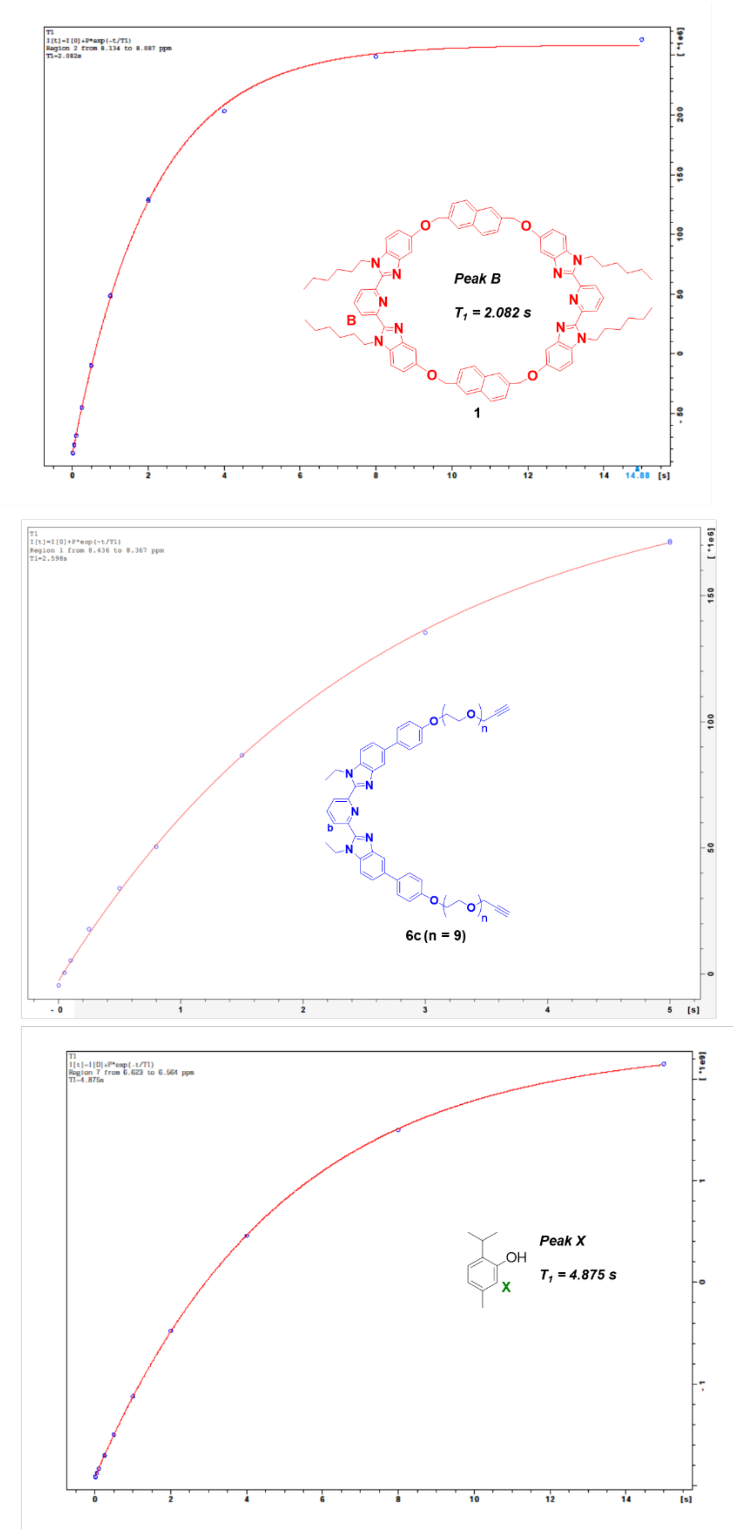

Figure S61. Plot showing relative magnetization versus time calculated from the three-parameter exponential fit (I0+P*exp(-t/T1)) for peak B (red), peak b (blue), and peak X (green) with 10 different spectra with varying inversion recovery delays.

The average number (moles) of macrocycle ($n_{ring\ extracted}$) and thread ($n_{thread\ extracted}$) present in the combined soluble fractions of SR-PCN series and tangled networks series is listed in Table S3, and ring retained (%) is plotted in Figure 5c. The residual from the combined soluble fraction was dissolved in 500 µL of $CDCl_3$ and added to an NMR tube. A thymol external standard for quantitative NMR *Trace*CERT® (0.0025 g, 150.22 g/mol) was dissolved in 100 µL of $CDCl_3$ to make the reference solution that was added to a Wilmad® coaxial insert tube. The same coaxial insert was added to each NMR tube containing the residual from the combined soluble fraction, and the outside of the insert was wiped with isopropyl alcohol and chloroform between samples to prevent contamination. The average mole number of macrocycle and thread present in each combined soluble fraction of SR-PCN for each a/b is listed in Table S3. With $X_{MC\ or\ thread}$ values, ring and thread content were calculated by using Equation S4.

$$Ring\ or\ thread\ retained\ (\%) = \frac{n_{ring\ or\ thread\ in\ the\ P3R} - n_{ring\ or\ thread\ extracted}}{n_{ring\ or\ thread\ in\ the\ P3R}} \times 100 \qquad (S4)$$

Where $n_{ring\ or\ thread\ in\ the\ P3R}$ is the total number of rings (one mole equivalent with respect to the P3R crosslinker) or threads (two mole equivalents with respect to the P3R crosslinker or tangled crosslinker) added to the polymerization as P3R crosslinker or tangled crosslinker in the prepolymer solution.

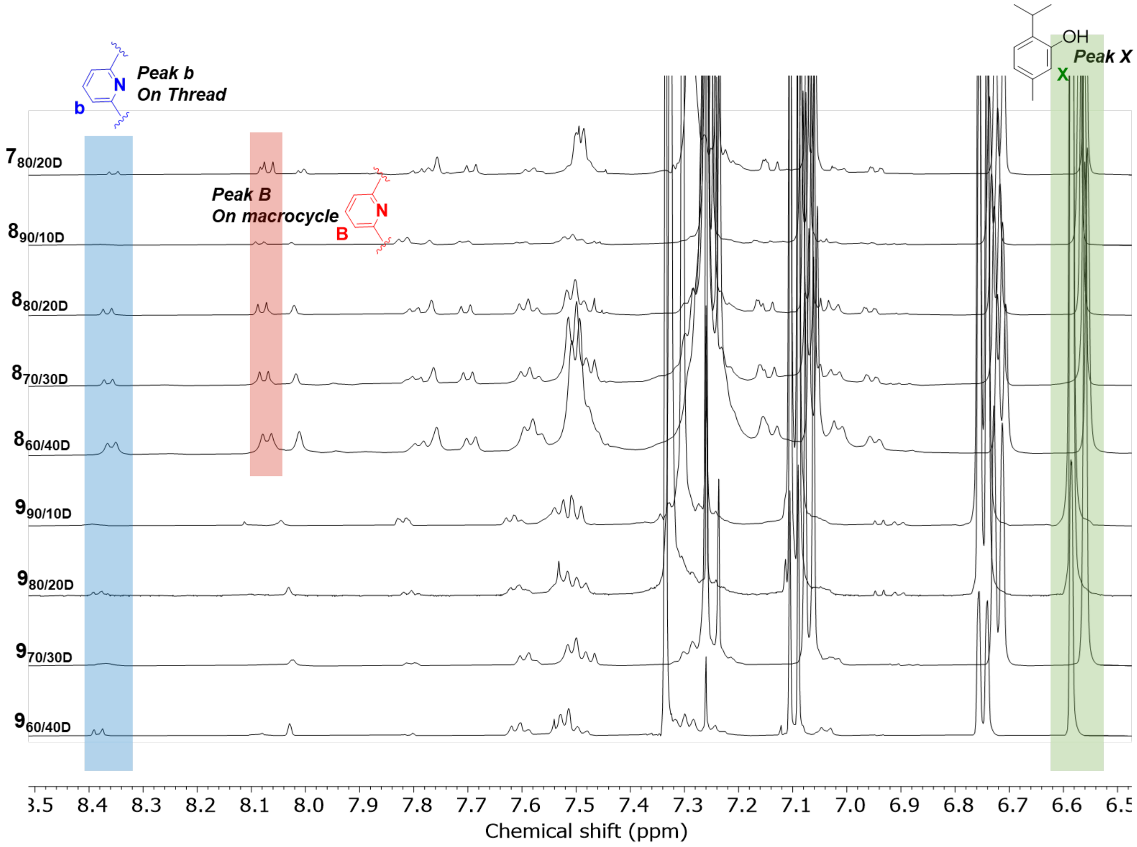

Figure S62. Partial ^{1}H-NMR (500 MHz, 25°C, $CDCl_3$) of the combined soluble fractions of SR-PCN $\mathbf{8_{a/bD}}$ series and $\mathbf{7_{80/20D}}$ and tangled networks $\mathbf{9_{a/bD}}$ series containing thymol as an external NMR standard. The integrals of the doublet on the pyridine ring at 8.08 ppm (B, red) for MC **1** (N=4) I_{MC} and for thread at 8.3 ppm (b, blue), I_{Thread}, and the singlet at 6.58 ppm (X, green) for thymol (N=1), I_{thymol}, were used to calculate the moles of macrocycle **1** and thread **6c** in each soluble fraction.

Table S3. NMR experiment parameters for ring and thread contents

	n_{P3R} (mol)	$n_{ring\ in\ the\ P3R}$ (mol)	$n_{thread\ in\ the\ P3R}$ (mol)	m_{PEG} (mol)	I_{MC}/I_{thymol}	$n_{ring\ extracted}$ (mol)	I_{thread}/I_{thymol}	$n_{thread\ extracted}$ (mol)	*Thread retained %*
**8$_{90/10D}$**	0.904E-06 mol	0.904E-06 mol	1.808E-06 mol	8.1E-06 mol	0.0282	0.117E-06 mol	0.00765	0.0635E-06 mol	96.5%
**9$_{90/10D}$**	0.904E-06 mol as **6c$_2$:Zn(II)**	N/A	1.808E-06 mol	8.1E-06 mol	N/A	N/A	0.00394	0.0327E-06 mol	98.2%
**8$_{80/20D}$**	1.808E-06 mol	1.808E-06 mol	3.616E-06 mol	7.2E-06 mol	0.0731	0.304E-06 mol	0.0433	0.359 E-06 mol	90.1%
**9$_{80/20D}$**	1.808E-06 mol as **6c$_2$:Zn(II)**	N/A	3.616E-06 mol	7.2E-06 mol	N/A	N/A	0.0192	0.159 E-06 mol	95.6%
**8$_{70/30D}$**	2.712E-06 mol	2.712E-06 mol	5.424E-06 mol	6.3E-06 mol	0.0931	0.417E-06 mol	0.0526	0.437 E-06 mol	92.0%
**9$_{70/30D}$**	2.712E-06 mol as **6c$_2$:Zn(II)**	N/A	5.424E-06 mol	6.3E-06 mol	N/A	N/A	0.0240	0.199 E-06 mol	96.3%
**8$_{60/40D}$**	3.616E-06 mol	3.616E-06 mol	7.232E-06 mol	5.4E-06 mol	0.2290	0.989E-06 mol	0.145	1.20 E-06 mol	83.3%
**9$_{60/40D}$**	3.616E-06 mol as **6c$_2$:Zn(II)**	N/A	7.232E-06 mol	5.4E-06 mol	N/A	N/A	0.0533	0.442 E-06 mol	93.9%
**7$_{80/20D}$**	1.808E-06 mol	1.808E-06 mol	3.616E-06 mol	7.2E-06 mol	0.0842	0.350E-06 mol	0.0416	0.345 E-06	90.4%

Zn(II) Assembly of **6c$_2$:Zn(II)**

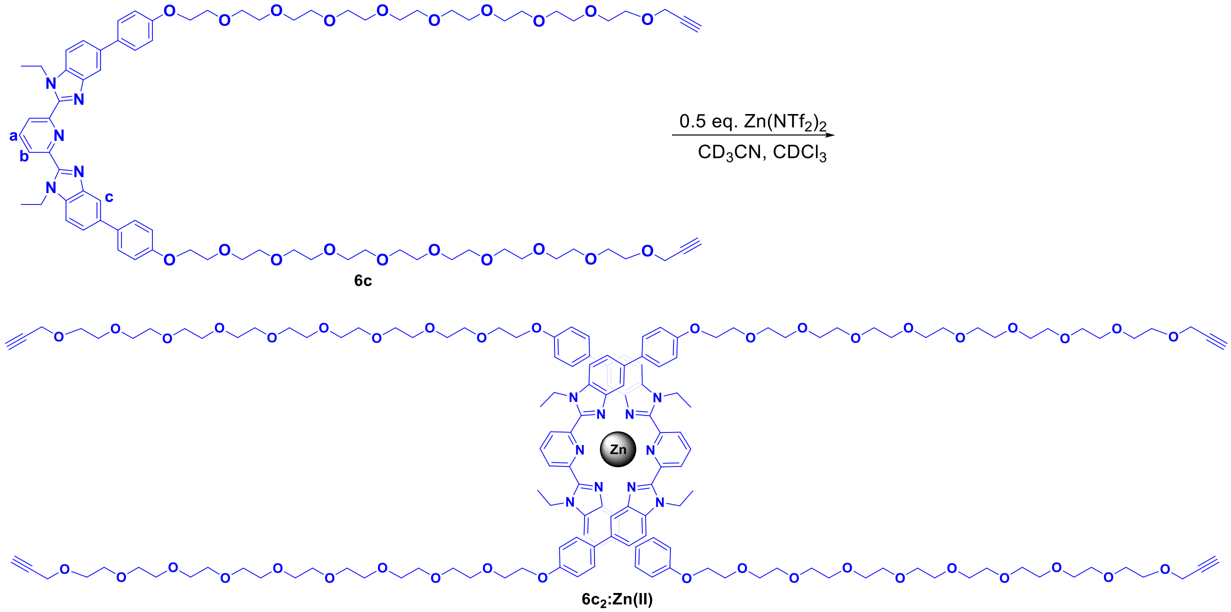

Figure S63. Formation of **6c$_2$:Zn(II)**.

Thread **6c** (510 mg, 0.359 mmol) was dissolved in chloroform-*d* (4 mL) and added to an NMR tube. A stock solution of zinc di[bis(trifluoromethylsulfonyl)imide] (157 mg, 0.251 mmol) in acetonitrile-d_3 (2 mL) was added until no free (unbound) Bip peaks appeared at ~0.5 equivalents of zinc di[bis(trifluoromethylsulfonyl)imide]. The complete disappearance of the doublets at 8.37 ppm indicates that all Bip ligands are bound with Zn^{2+} ions in a 2:1 Bip:metal ratio. The resulting **6c$_2$:Zn(II)** solution was dried in vacuo to obtain a yellow oil that was redissolved in 5% acetonitrile-d_3 in chloroform-*d* (2 mL) and stirred at 45°C for 1 d to allow equilibration. The solvent was then removed in vacuo resulting in a yellow oil that was stored in the freezer at -37°C before use.

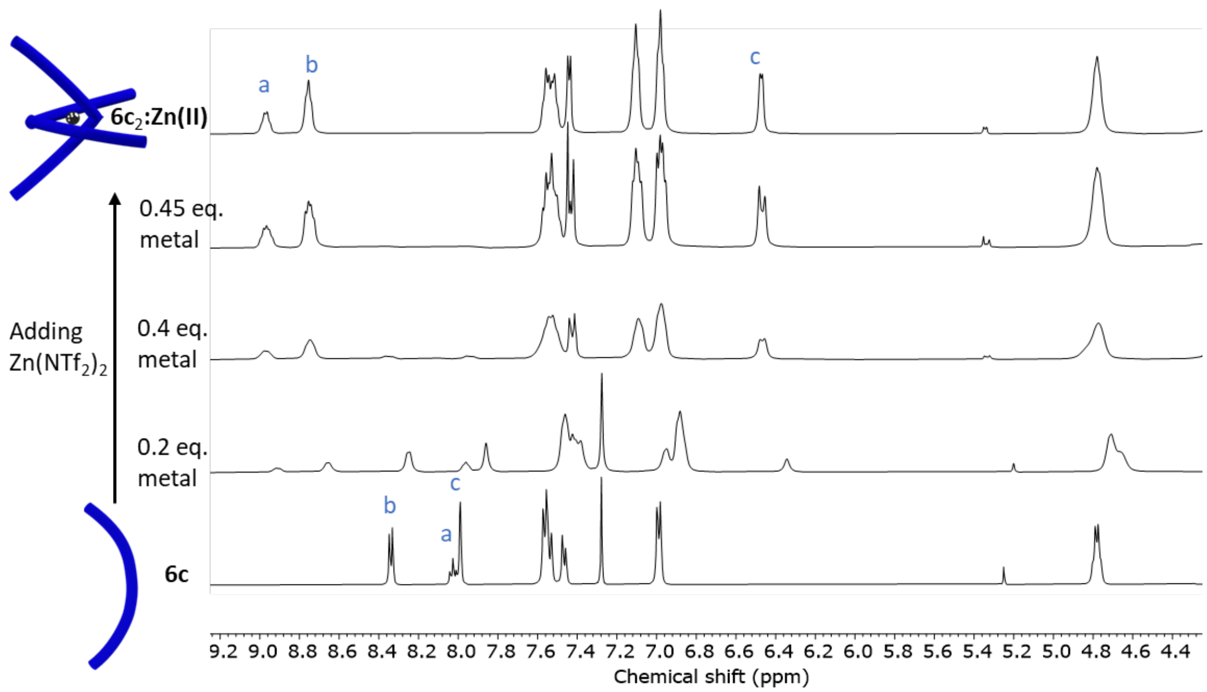

Figure S64. Partial ^{1}H-NMR overlay (500 MHz, 25°C, increasing acetonitrile-d_3 in chloroform-*d* increasing upwards) of metal addition during NMR titrations.

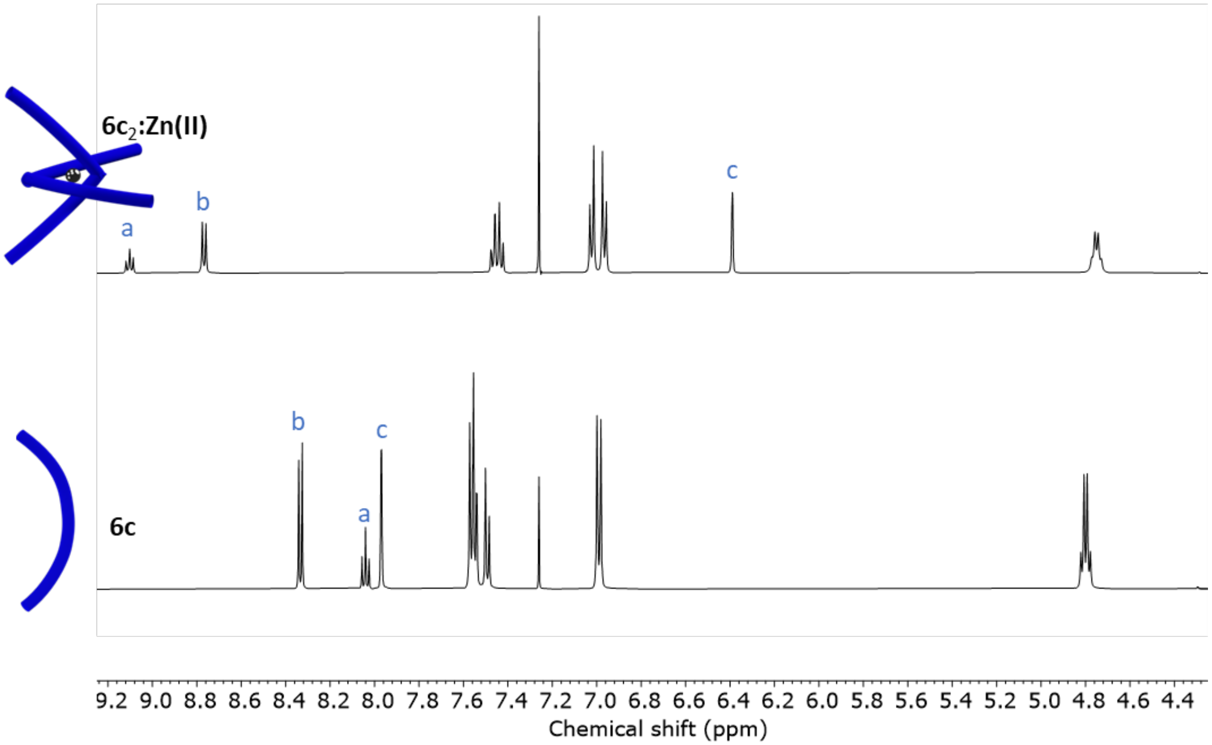

Figure S65. Partial ^{1}H-NMR overlay (500 MHz, 25°C) of 10 mM **6c$_2$:Zn(II)** and 20 mM **6c** (5% acetonitrile-d_3 in chloroform-*d*) after equilibration.

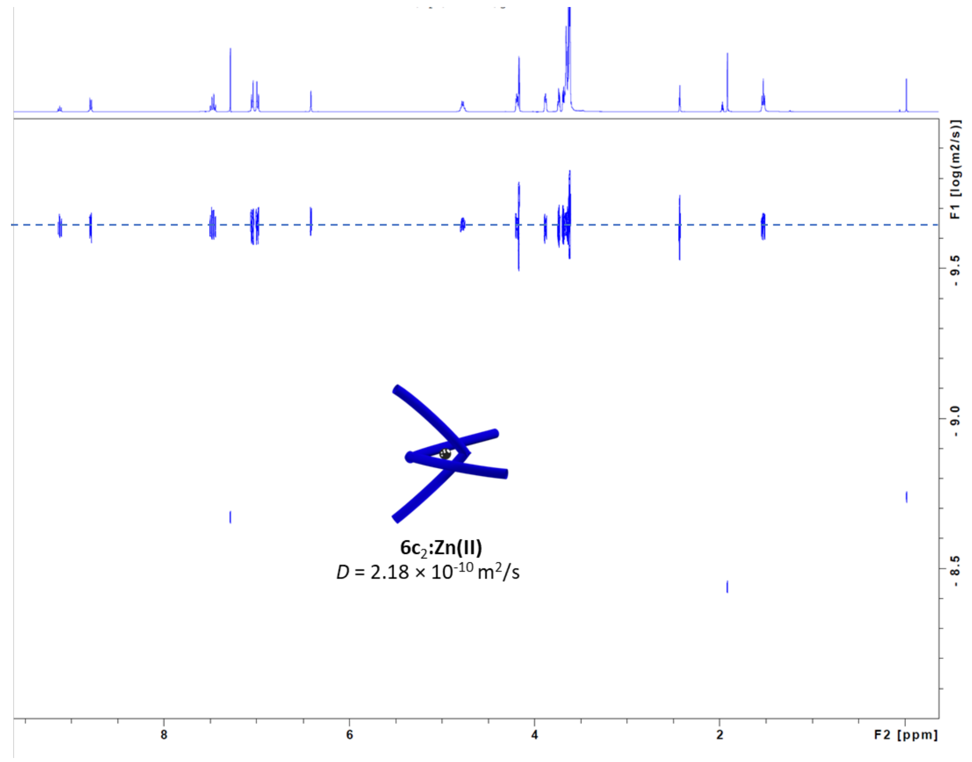

Figure S66. DOSY Spectrum (500 MHz, 25°C) of 10 mM **6c$_2$:Zn(II)** (5% acetonitrile-d_3 in chloroform-d).

Control Network $9_{a/b}$ Synthesis

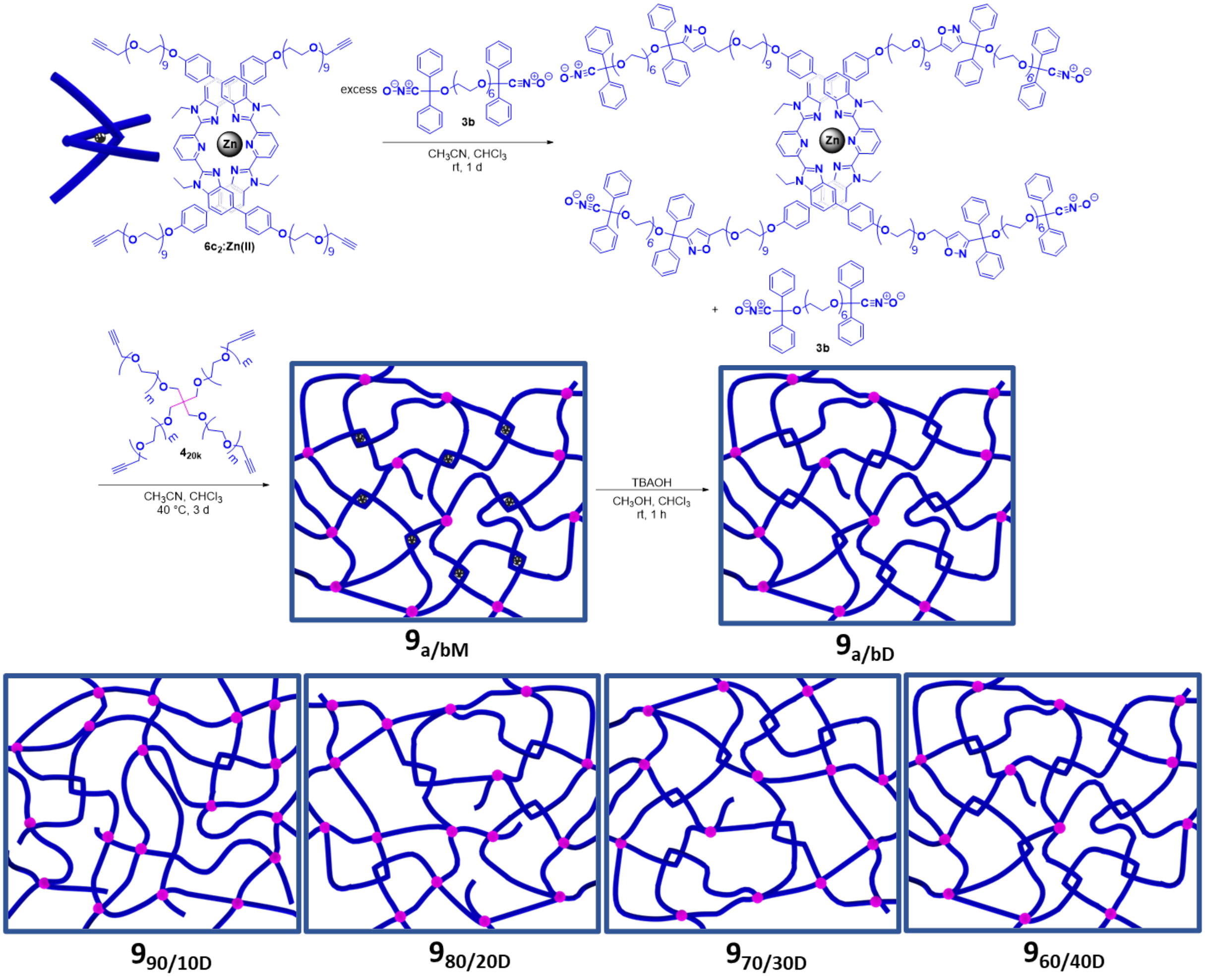

Figure S67. Synthesis of control network $\mathbf{9_{a/b}}$ series. Polymerization of control network $\mathbf{9_{a/bM}}$ series with varying amounts of $\mathbf{6c_2}$**:Zn(II)** ($n_{crossing}$ mol, *b* mol%) and $\mathbf{4_{20k}}$ (m_{PEG} mol, *a* mol%), and demetalation with a dilute solution of tetrabutylammonium hydroxide (TBAOH) to $\mathbf{9_{a/bD}}$.

Monomer **3b** (12.5 mg, 0.0179 mmol) was added to a 2 dram glass shell vial with plug style closures. A stock solution (250 μL) of $\mathbf{6c_2}$**:Zn(II)** ($n_{crossing}$ mol, see Table S4) in 30% acetonitrile in chloroform was added. The vial was capped and sealed with parafilm and electrical tape. The reaction mixture was stirred for 24 h. Then, 4-arm PEG-alkyne $\mathbf{4_{20k}}$ (M_n = 20.0 kgmol^{-1}, m_{PEG} mol, see Table S4) was added. 100 μL of 30% acetonitrile in chloroform (containing 1.7 mg of TMS silica for DLS microrheology measurements) was added. The reaction mixture was stirred for 2 min, and the stir bar was removed. The vial was capped and sealed with parafilm and electrical tape and left to gel at 40°C for 72 h. A spatula was used to scoop out the crude metalated control network ($\mathbf{9_{a/bM}}$) and transfer to a Teflon dish to dry in a fume hood overnight. Then, the crude $\mathbf{9_{a/bM}}$ was dried in vacuo (48 h) at 50 °C. The mass of the crude, dried $\mathbf{9_{a/bM}}$ was recorded. To remove low molecular weight components from the crude $\mathbf{9_{a/bM}}$ and calculate the gel fraction (GF), the crude, dried $\mathbf{9_{a/bM}}$ was added in a beaker. Chloroform (500 mL) was added. The solution was heated to 55°C on a hot plate for 4 h. A watch glass was added on top of the beaker to mitigate evaporation. The swollen, washed $\mathbf{9_{a/bM}}$ was transferred to a Teflon dish to dry in a fume hood overnight. Then, the washed $\mathbf{9_{a/bM}}$ was dried in vacuo (48 h) at 50 °C. The mass of the dried, washed $\mathbf{9_{a/bM}}$ was recorded.

Table S4. Composition of **control network 9**$_{\mathbf{a/bM}}$ series

Control network	Monomer **3b**	4-arm PEG-Alkyne **4**$_{\mathbf{20k}}$, m_{PEG} mol	**6c**$_{2}$**:Zn(II)**, $n_{crossing}$ mol	**6c**$_{2}$**:Zn(II)**, Stock solution concentration
**9**$_{\mathbf{90/10M}}$	12.5 mg, 1.8E-05 mol	162 mg, 8.1E-06 mol	3.12 mg, 0.9E-06 mol	12.4 mg/mL
**9**$_{\mathbf{80/20M}}$	12.5 mg, 1.8E-05 mol	144 mg, 7.2E-06 mol	6.24 mg, 1.8E-06 mol	24.6 mg/mL
**9**$_{\mathbf{70/30M}}$	12.5 mg, 1.8E-05 mol	126 mg, 6.3E-06 mol	9.36 mg, 2.7E-06 mol	37.4 mg/mL
**9**$_{\mathbf{60/40M}}$	12.5 mg, 1.8E-05 mol	108 mg, 5.4E-06 mol	12.5 mg, 3.6E-06 mol	50.0 mg/mL

The gel fraction (GF) of **9**$_{\mathbf{a/bM}}$ was calculated from modified Equation S1:

$$\text{GF (wt \%)} = \frac{m_{dried,washed,metalated}}{m_{crude,dried,metalated}} \text{ x } 100 \qquad \text{(S1)}$$

Where $m_{crude,dried,metalated}$ is the weight of the crude, dried **9**$_{\mathbf{a/bM}}$ after gelation, and $m_{dried,washed,metalated}$ is the weight of the dried, washed **9**$_{\mathbf{a/bM}}$ after washing.

The washed **9**$_{\mathbf{a/bM}}$ (Figure S68) exhibited a yellow color under ambient light and a yellow fluorescence under 365 nm UV light on account of the Bip$_2$/Zn(II) complexes and qualitatively confirms the successful incorporation of **6c**$_{2}$**:Zn(II)** moieties into the network.

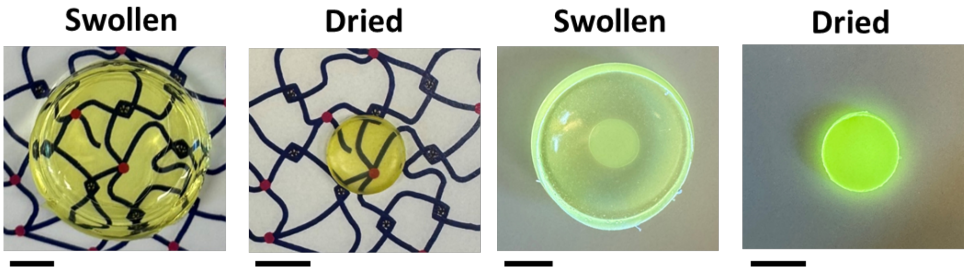

Figure S68. Pictures of **9**$_{\mathbf{a/bM}}$ under ambient light (left) and under 365 nm UV light (right), 1 cm scale bar. **9**$_{\mathbf{80/20M}}$ pictured.

<u>Demetalation and Washing</u>

To demetalate, the dried, washed **9**$_{\mathbf{a/bM}}$ was added to a Teflon dish. 35 mL of chloroform and 5 mL of methanol were added to swell the network, and then tetrabutylammonium hydroxide solution (1M in methanol, 50 µL) was added dropwise to demetalate for 1 h, resulting in a slow color change from yellow to light blue under 365 nm UV light, the fluorescence color of the free ligand, consistent with the decomplexation/demetalation of the gels. This process was repeated three times. Then, the swollen demetalated control network (**9**$_{\mathbf{a/bD}}$) was transferred to a second Teflon dish. 20 mL chloroform and 20 mL methanol were added to soak the network for 1 h. This process was repeated six times. Then, swollen **9**$_{\mathbf{a/bD}}$ was added in a beaker. Chloroform (500 mL) was added. The solution was heated to 55°C on a hot plate for 4 h. A watch glass was added on top of the beaker to mitigate evaporation. The swollen, washed **9**$_{\mathbf{a/bD}}$ was transferred to a Teflon dish to dry in a fume hood overnight. Then, washed **9**$_{\mathbf{a/bD}}$ was dried in vacuo (48 h) at 50 °C. The mass of the dried, washed **9**$_{\mathbf{a/bD}}$ was recorded.

The gel fraction (GF) of **9**$_{\mathbf{a/bD}}$ was calculated from modified Equation S2:

$$\text{GF (wt \%)} = \frac{m_{dried,washed,demetalated}}{m_{crude,dried,metalated} - m_{zn(ntf2)2}} \text{ x } 100 \qquad \text{(S2)}$$

Where $m_{crude,dried,metalated}$ is the weight of the crude, dried **9**$_{\mathbf{a/bM}}$ after gelation, $m_{dried,washed,demetalated}$ is the weight of the dried, washed **9**$_{\mathbf{a/bD}}$ after washing, and $m_{zn(ntf2)2}$ is the weight of zinc di[bis(trifluoromethylsulfonyl)imide] from **6c**$_{2}$**:Zn(II)** to account for the loss of metal and counterion masses during demetalation.

The washed **9**$_{\mathbf{a/bD}}$ (Figure S69) exhibited a blue fluorescence under 365 nm UV light on account of the Bip ligands and qualitatively confirms the successful incorporation of Bip moieties into the network.

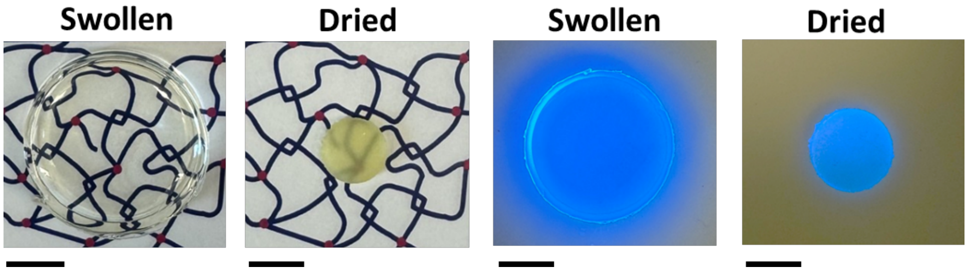

Figure S69. Pictures of $\mathbf{9_{a/bD}}$ under ambient light (left) and under 365 nm UV light (right), 1 cm scale bar. $\mathbf{9_{80/20D}}$ pictured.

Control Network $\mathbf{10_{a/b}}$ Synthesis and Washing

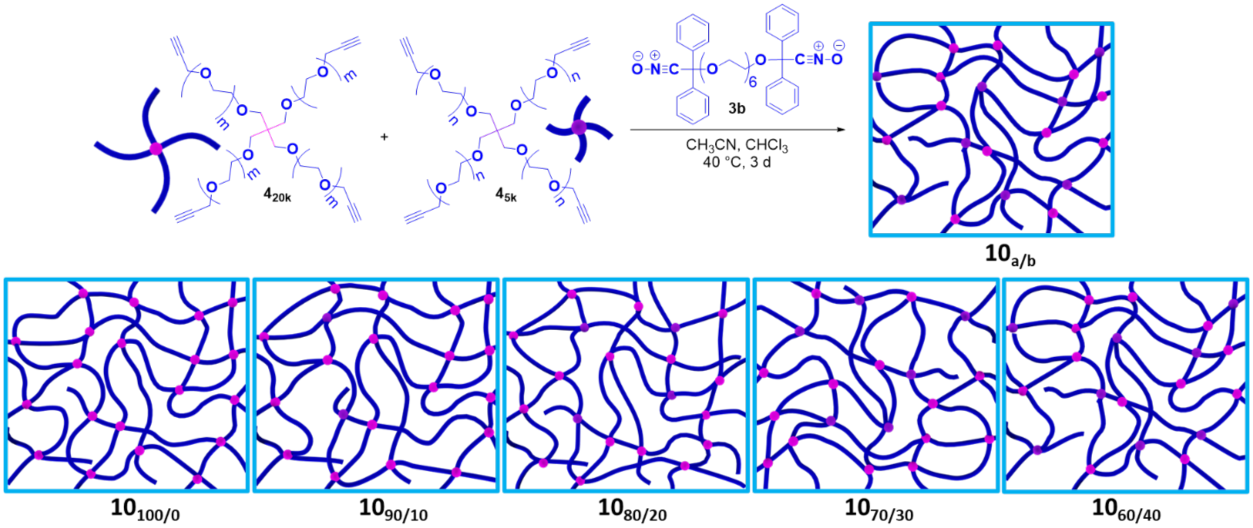

Figure S70. Synthesis of control network $\mathbf{10_{a/b}}$ series. Polymerization of control network $\mathbf{10_{a/b}}$ series with varying amounts of $\mathbf{4_{5k}}$ (n_{PEG} mol, *b* mol%) and $\mathbf{4_{20k}}$ (m_{PEG} mol, *a* mol%).

Monomer **3b** (12.5 mg, 0.0179 mmol) and 4-arm PEG-alkyne $\mathbf{4_{20k}}$ (M_n = 20.0 kgmol^{-1}, m_{PEG} mol, see Table S5) were added to a 2 dram glass shell vial with plug style closures. A stock solution (100 µL) of 4-arm PEG-alkyne $\mathbf{4_{5k}}$ (M_n = 5.0 kgmol^{-1}, n_{PEG} mol, see Table S5) in 30% acetonitrile in chloroform was added. Then, 250 µL of 30% acetonitrile in chloroform (containing 1.7 mg of TMS silica for DLS microrheology measurements) was added. The reaction mixture was stirred for 2 min, and the stir bar was removed. The vial was capped and sealed with parafilm and electrical tape and left to gel at 40°C for 72 h. A spatula was used to scoop out the crude control network ($\mathbf{10_{a/b}}$) and transfer to a Teflon dish to dry in a fume hood overnight. Then, the crude $\mathbf{10_{a/b}}$ was dried in vacuo (48 h) at 50 °C. The mass of the crude, dried control network was recorded. To remove low molecular weight components from the crude $\mathbf{10_{a/b}}$ and calculate the gel fraction (GF), the crude, dried $\mathbf{10_{a/b}}$ was added in a beaker. Chloroform (500 mL) was added. The solution was heated to 55°C on a hot plate for 4 h. A watch glass was added on top of the beaker to mitigate evaporation. The swollen $\mathbf{10_{a/b}}$ was added to a Teflon dish. 35 mL of chloroform and 5 mL of methanol were added to soak the network for 1 h. This process was repeated three times. Then, the swollen $\mathbf{10_{a/b}}$ was transferred to a second Teflon dish. 20 mL chloroform and 20 mL methanol were added to soak the network for 1 h. This process was repeated for six times. Then, the swollen $\mathbf{10_{a/b}}$ was added in a beaker. Chloroform (500 mL) was added. The solution was heated to 55°C on a hot plate for 4 h. A watch glass was added on top of the beaker to mitigate evaporation. The swollen, washed $\mathbf{10_{a/b}}$ was transferred to a Teflon dish to dry in a fume hood overnight. Then, the washed $\mathbf{10_{a/b}}$ was dried in vacuo (48 h) at 50 °C. The mass of the dried, washed $\mathbf{10_{a/b}}$ was recorded.

Table S5. Composition of **control network 10$_{a/b}$** series

Control network	Monomer **3b**	4-arm PEG-Alkyne **4$_{20k}$**, m_{PEG} mol	4-arm PEG-Alkyne **4$_{5k}$**, n_{PEG} mol	4-arm PEG-Alkyne **4$_{5k}$**, stock solution concentration
**10$_{100/0}$**	12.5 mg, 1.8E-05 mol	180.0 mg, 9.0E-06 mol	0 mg, 0 mol	0 mg/mL
**10$_{90/10}$**	12.5 mg, 1.8E-05 mol	162.0 mg, 8.1E-06 mol	4.5 mg, 0.9E-06 mol	45.0 mg/mL
**10$_{80/20}$**	12.5 mg, 1.8E-05 mol	144.0 mg, 7.2E-06 mol	9.0 mg, 1.8E-06 mol	90.0 mg/mL
**10$_{70/30}$**	12.5 mg, 1.8E-05 mol	126.0 mg, 6.3E-06 mol	13.5 mg, 2.7E-06 mol	135.0 mg/mL
**10$_{60/40}$**	12.5 mg, 1.8E-05 mol	108.0 mg, 5.4E-06 mol	18.0 mg, 3.6E-06 mol	180.0 mg/mL

The gel fraction (GF) of **10$_{a/b}$** was calculated from Equation S5:

$$\text{GF (wt \%)} = \frac{m_{dried,washed}}{m_{crude,dried}} \text{ x } 100 \qquad \text{(S5)}$$

Where $m_{crude,dried}$ is the weight of the crude, dried **10$_{a/b}$** after gelation, and $m_{dried,washed}$ is the weight of the dried, washed **10$_{a/b}$** after washing.

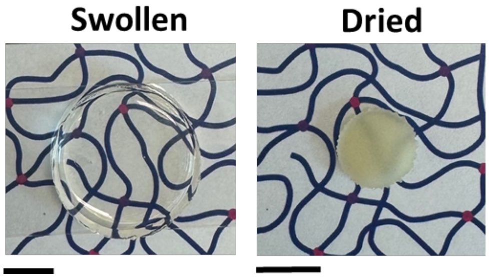

Figure S71. Pictures of **10$_{a/b}$** under ambient light, 1 cm scale bar. **10$_{100/0}$** pictured.

Gel Fraction (GF) for **9$_{a/b}$**

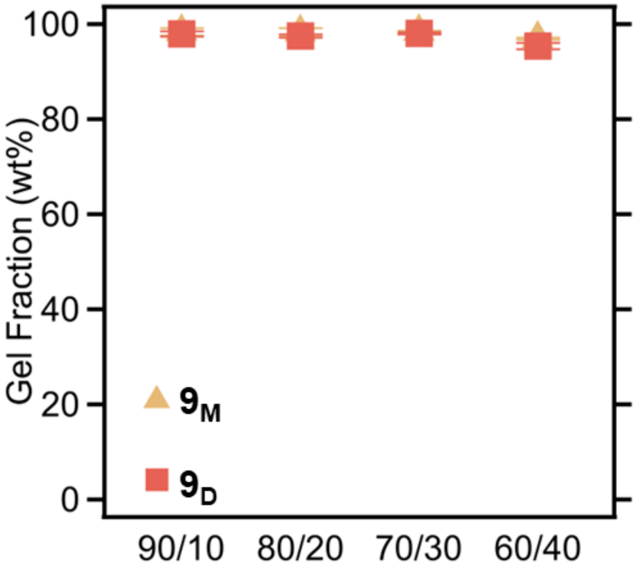

Figure S72. Gel fractions (GFs) for control network **9$_{a/bM}$** and **9$_{a/bD}$** calculated from modified Equations S1 and S2, respectively.

Gel Fraction (GF) for **10**$_{a/b}$

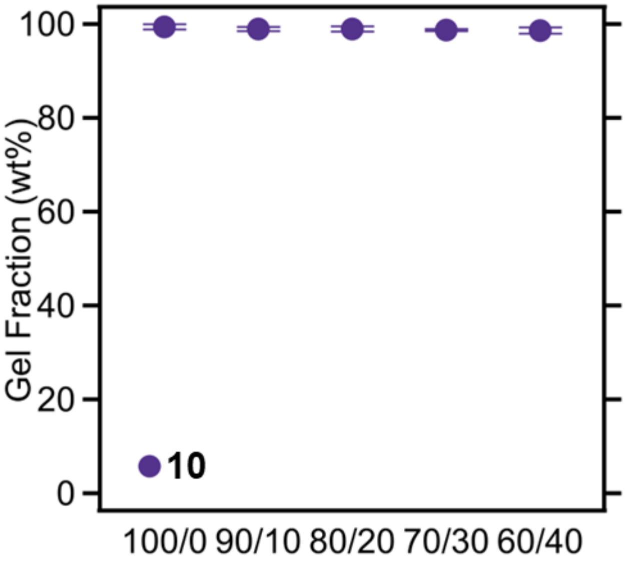

Figure S73. Gel fractions (GFs) for control network **10**$_{a/b}$ calculated from Equation S5.

Swelling procedure and swelling ratio (vol%)

A sieve filtration method was employed to ensure repeatable, reproducible measurement of swelling, achieved via fast and complete removal of excess fluid from the gel on a wire mesh sieve using vacuum filtration.[16] The mass of a 316 stainless steel wire sieve (250 x 250 mesh size, 0.0024" opening size) was recorded as W_1. The washed, dried demetalated slide-ring polycatenane network (**8**$_{a/bD}$), the washed, dried demetalated control network with (**9**$_{a/bD}$), and the washed, dried control network (**10**$_{a/b}$) were dried in vacuo (48 h) at 50 °C, and their dry masses were recorded as W_0.

The network was then submerged in solvent (50 mL) for 24 hours to allow the network to reach its swelling equilibrium. The swollen network was poured onto the wire sieve under vacuum to remove excess solvent from the swollen network (and sieve), and the mass of the sieve with the swollen network on it was recorded as W_2. The average of three weight measurements W_2 was calculated for each sample (and multiple samples for each type of network) and used to calculate the swelling ratio, which is defined as the ratio of the swollen volume (V) to the dry volume (V_0), Equation S6:

$$\text{Swelling Ratio (vol\%)} = \frac{V}{V_0} = \frac{W_2 - W_1 - W_0}{W_0} \div \mathrm{D} \times 100 \qquad \text{(S6)}$$

which is based on the weight of the dry network (W_0) and that of a wire mesh (W_1), and the density of the solvent (D).

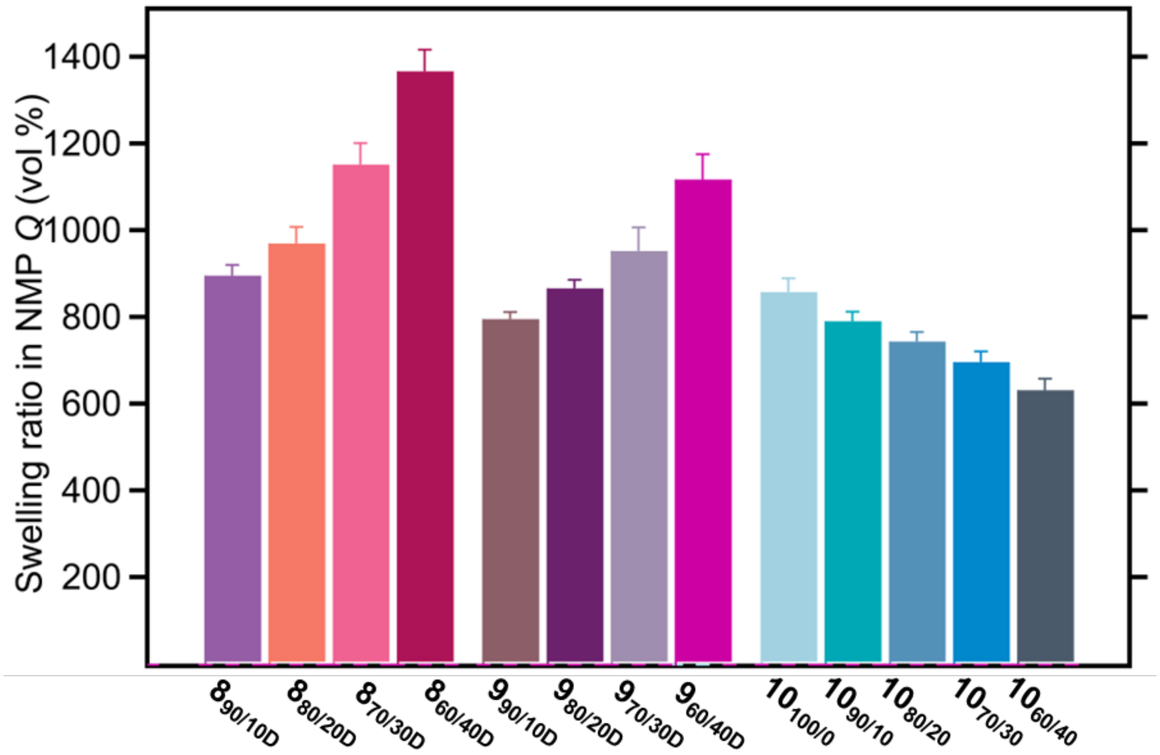

Figure S74. Volume-based swelling ratios calculated from Equation S6 for demetalated slide-ring polycatenane network (**8**$_{a/bD}$), the demetalated control network (**9**$_{a/bD}$), and the control network (**10**$_{a/b}$) in NMP.

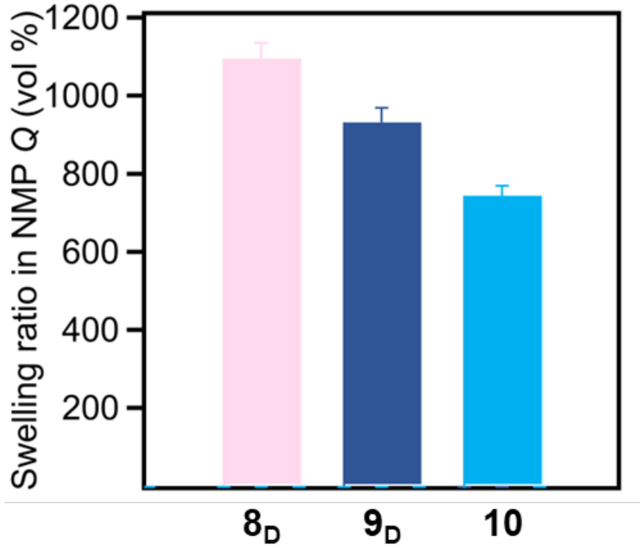

Figure S75. The average volume-based swelling ratios calculated from Equation S6 for demetalated slide-ring polycatenane network (**8**$_{a/bD}$), the demetalated control network (**9**$_{a/bD}$), and the control network (**10**$_{a/b}$) in NMP.

Small-amplitude oscillatory compression (SAOC) in NMP

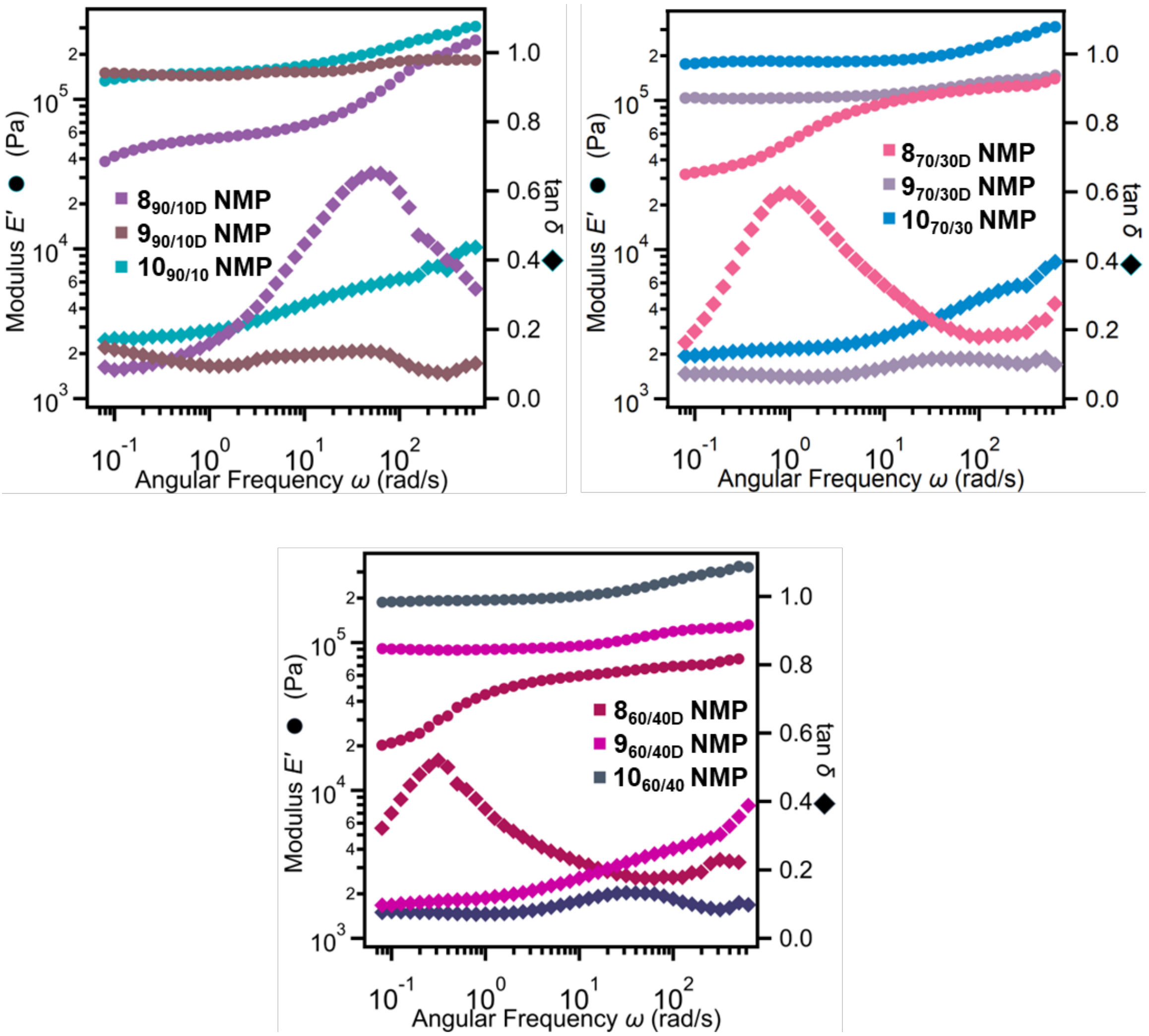

Figure S76. Small-amplitude oscillatory compression (SAOC) frequency sweeps showing storage modulus E' (filled circles) and tan δ (filled diamonds) for: control network (**$10_{a/b}$**), the demetalated control network (**$9_{a/bD}$**), and the demetalated slide-ring polycatenane network (**$8_{a/bD}$**) swollen in NMP.

Storage and loss moduli from small-amplitude oscillatory compression (SAOC) in NMP

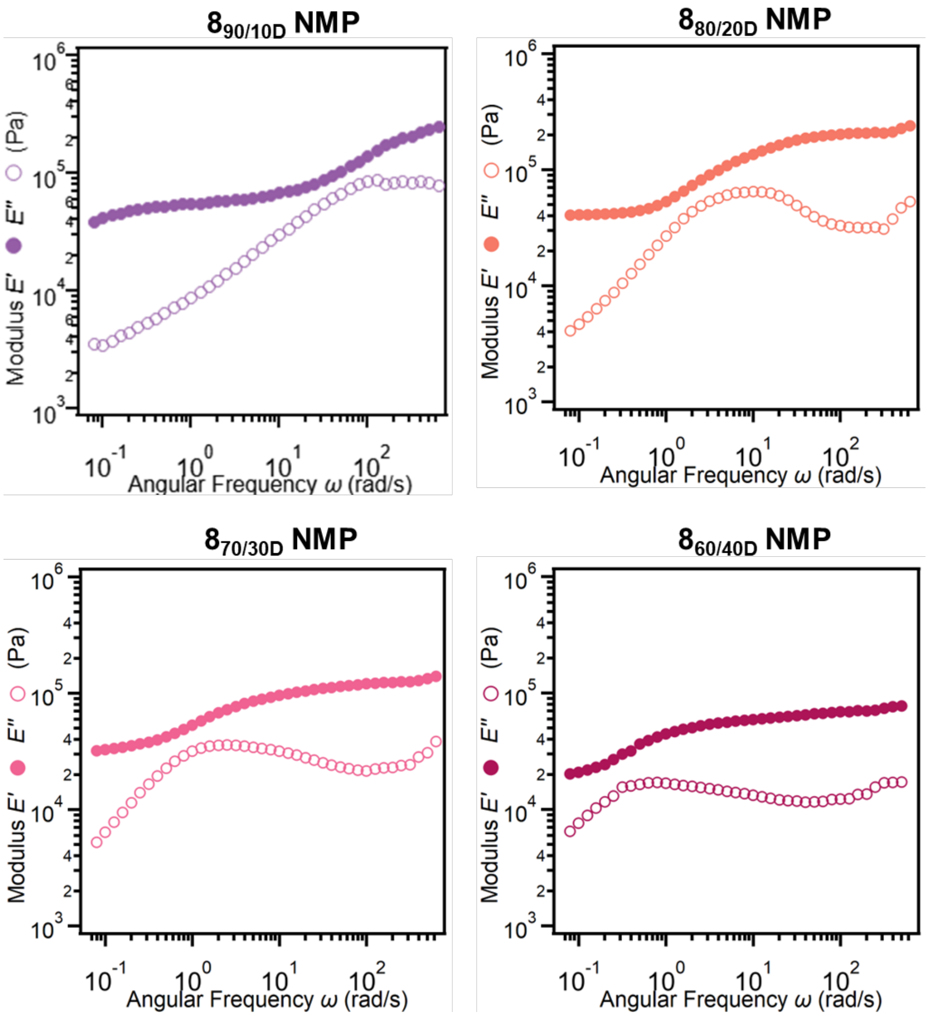

Figure S77. Small-amplitude oscillatory compression (SAOC) frequency sweeps showing storage modulus *E'* (filled circles) and loss modulus *E''* (open circles) for demetalated slide-ring polycatenane network (**8**$_{a/bD}$) swollen in NMP.

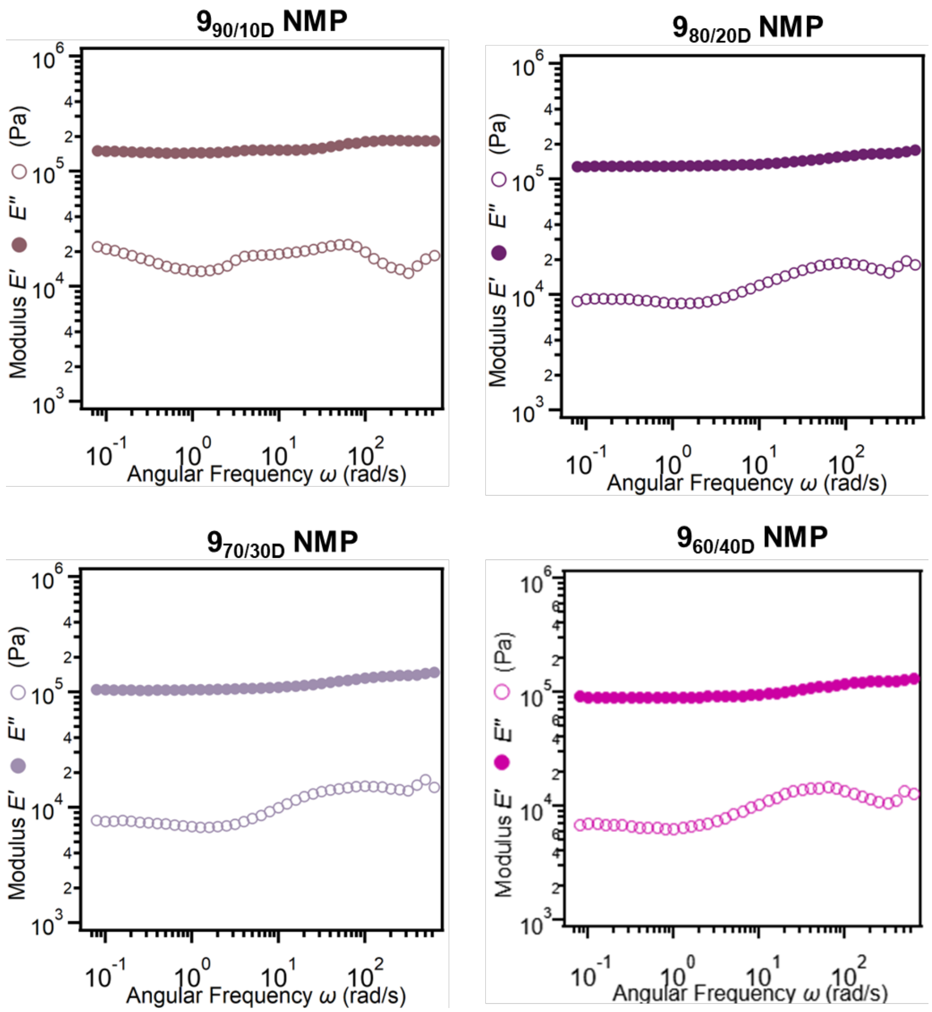

Figure S78. Small-amplitude oscillatory compression (SAOC) frequency sweeps showing storage modulus *E'* (filled circles) and loss modulus *E''* (open circles) for demetalated control network (**9**$_{a/bD}$) swollen in NMP.

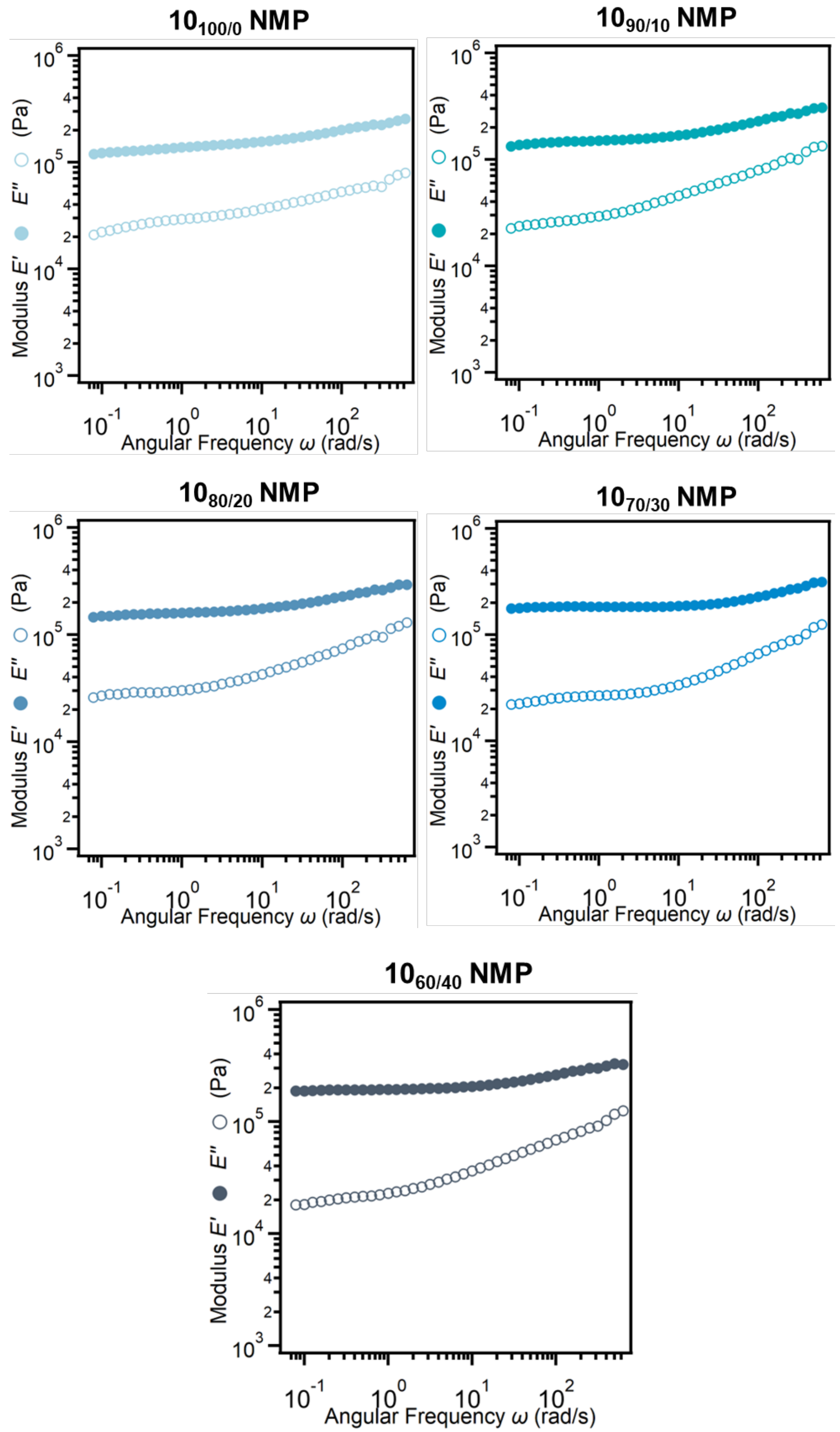

Figure S79. Small-amplitude oscillatory compression (SAOC) frequency sweeps showing storage modulus E' (filled circles) and loss modulus E'' (open circles) for the control network ($\mathbf{10}_{a/b}$) swollen in NMP.

Tensile testing in NMP

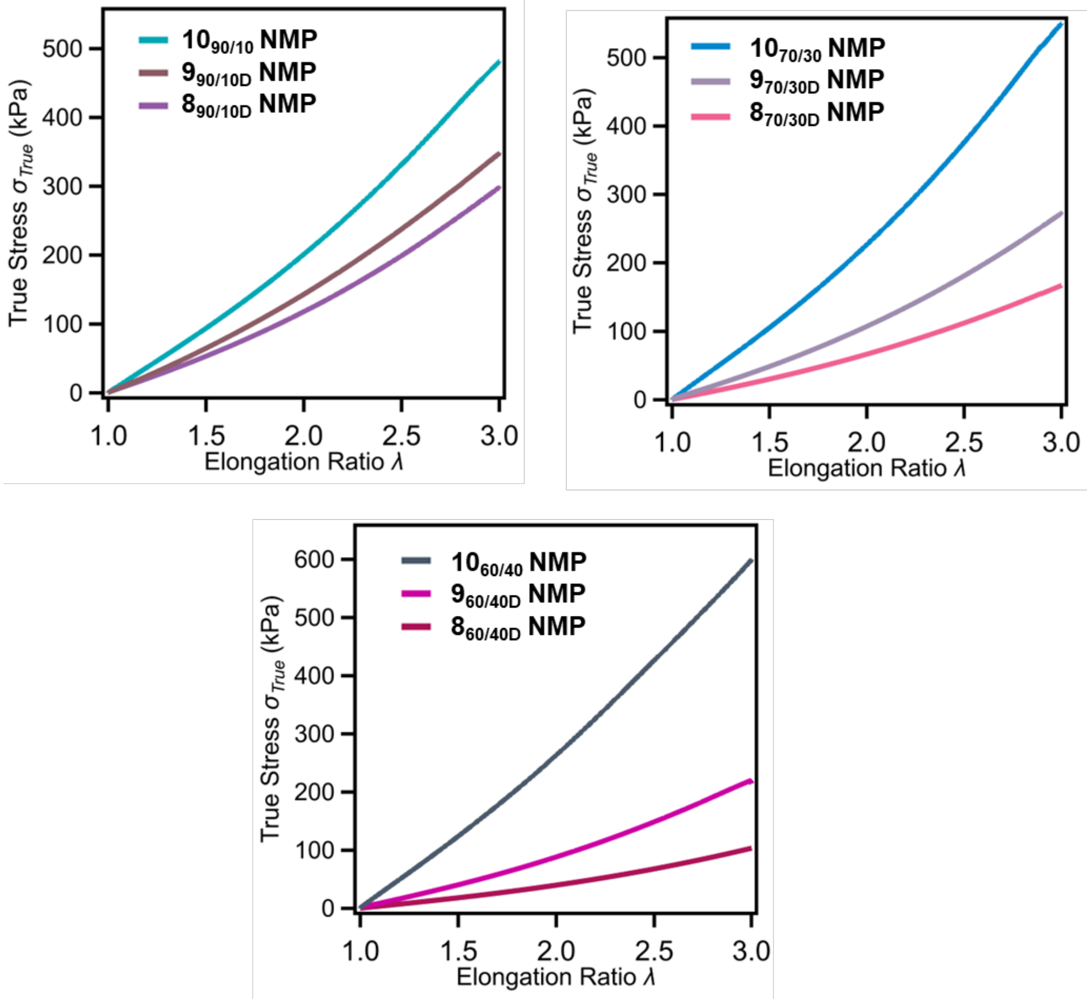

Figure S80. True stress-elongation ratio curves of swollen demetalated slide-ring polycatenane network (**8**$_{\mathbf{a/bD}}$), demetalated control networks (**9**$_{\mathbf{a/bD}}$) and covalent control networks (**10**$_{\mathbf{a/b}}$) swollen in NMP with a strain rate of 20% strain min^{-1}

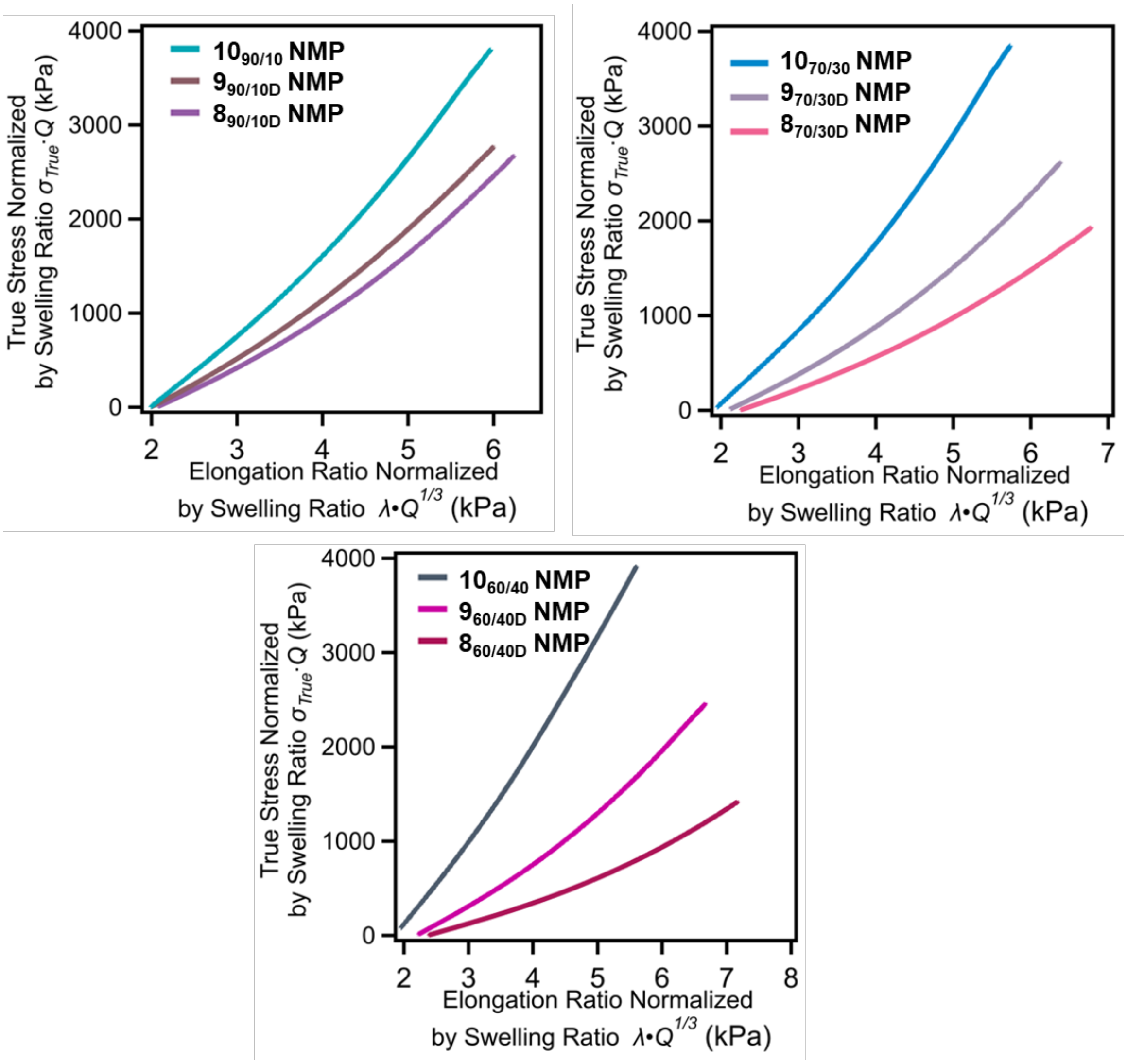

Figure S81. True stress normalized by swelling ratio-elongation ratio curves of swollen demetalated slide-ring polycatenane network (**8**$_{\mathbf{a/bD}}$), demetalated control networks (**9**$_{\mathbf{a/bD}}$), and covalent control networks (**10**$_{\mathbf{a/b}}$) swollen in NMP with a strain rate of 20% strain min^{-1}. The true stress was normalized by the swelling ratio, and the elongation ratio was normalized by $Q^{1/3}$.

Coarse-Grained Molecular Dynamics (MD) Simulations

A coarse-grained representation of network strands was performed, modeling them as bead-spring chains of beads with diameter σ.[17] The pairwise interactions between any two beads separated by a distance r were described by the pure repulsive truncated-shifted Lennard-Jones (LJ) potential,

$$U_{\mathrm{LJ}}(r) = \begin{cases} 4\varepsilon_{\mathrm{LJ}}[(\sigma/r)^{12} - (\sigma/r)^{6} - (\sigma/r_{\mathrm{cut}})^{12} + (\sigma/r_{\mathrm{cut}})^{6}] & r \le r_{\mathrm{cut}} \\ 0 & r > r_{\mathrm{cut}} \end{cases} \quad \text{(S7)}$$

The cutoff radius for the pairwise interactions is set to $r_{\mathrm{cut}} = 2^{1/6}\sigma$. The pairwise interactions between beads given by Equation S7 were turned off for phantom network simulations. The connectivity of the beads into polymer chains and crosslinking bonds was described by the finite extensible nonlinear elastic (FENE) potential,[17]

$$U_{\mathrm{FENE}}(r) = -\frac{1}{2}k_{\mathrm{spring}}R_{\mathrm{max}}^2 \ln\left(1 - \frac{r^2}{R_{\mathrm{max}}^2}\right) \quad \text{(S8)}$$

where r is the distance between the two bonded beads. The spring constant is set to $k_{\mathrm{spring}} = 30k_BT/\sigma^2$, and the maximum bond length $R_{\mathrm{max}} = 1.5\sigma$. The repulsive part of the bond potential is described by the truncated-shifted LJ potential with $\varepsilon_{\mathrm{LJ}} = 1.0k_BT$ and $r_{\mathrm{cut}} = 2^{1/6}\sigma$. This set of interaction parameters gives an average bond length, $l = 0.965\sigma$. The equation of motion is integrated by the velocity-Verlet algorithm with a time step $\Delta t = 0.005\tau_{LJ}$, where $\tau_{LJ} = \sigma(m/k_BT)^{1/2}$ is the standard LJ time. All simulations were performed using LAMMPS[18] under 3-D periodic boundary conditions.

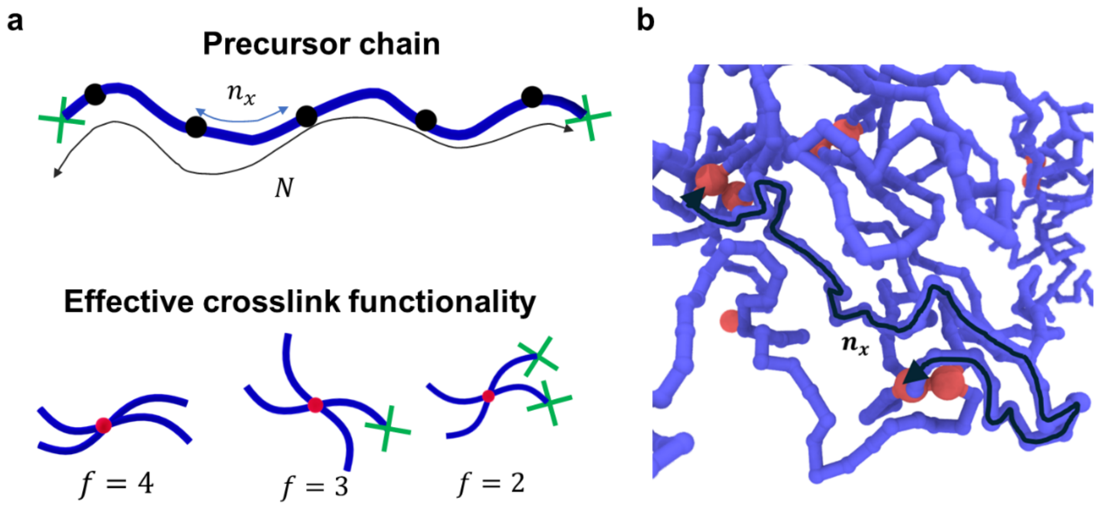

Figure S82. a) Schematic illustration of precursor chain featuring potential crosslinking sites (black dot), initializing the number of bonds between crosslinks, n_x, and different effective functionality of crosslinks (red dot). A dangling chain end is marked with a cross (green). b) Snapshot of the covalent network showing the network strand with n_x (blue) and covalent crosslink beads (red).

The preparation of networks with different crosslinks follows these steps:

(1) *Covalent Network*. The randomly crosslinked covalent networks (**Figure 7a**) are generated by crosslinking of noninteracting bead-spring chains (precursor chains) with a degree of polymerization (DP) of N = 1025 with the density $\rho = 0.85\ \sigma^{-3}$ of beads of diameter σ in the melt state. In addition, the number of bonds between crosslinks, n_x, is set to 150. Each crosslinking bond connects every n_x-*th* bead, starting from the 62nd bead from a chain end to a corresponding neighboring bead on the other chain by a bond (**Figure S82**). This crosslinking procedure results in a tetra-functional network with a narrowly distributed strand DP between crosslinking points, exhibiting a dispersity, $Đ$ = 1.02, calculated from the number- and weight-average strand DP obtained in the simulation. The total number of precursor chains in the covalent network is 1000. Following crosslinking, the network structure is equilibrated by performing an NVT ensemble simulation. The constant temperature is maintained using a Langevin thermostat with a damping time constant of 1.0τ, ensuring thermal equilibration while preserving the structural integrity of the network.

(2) *Doubly Threaded Slide Ring-Polycatenane Network* (dtSR-PCN). The preparation of the SR-PCN begins by swelling the covalent network under the condition of constant pressure P = 0 and constant temperature T set to unity, controlled through the Nosé-Hoover barostat and thermostat. This swelling process allows the network to expand, facilitating subsequent modifications. After swelling, randomly selected 10%, 20%,

30%, and 40% of the total covalent crosslinking bonds are designated for replacement. However, during the replacement process, only bonds not connected to dangling ends are considered to prevent any topological changes. The selected crosslinking bonds are removed and substituted with slide rings, each constructed from 12 beads connected by 12 bonds to form a closed-loop structure (**Figure 7a**). To ensure the slide rings maintain a regular dodecagonal shape, an additional harmonic angle potential is applied to the bond angles between adjacent slide-ring bonds:

$$U_{bend}(\theta) = K(\theta - \theta_0)^2 \quad \text{(S9)}$$

where θ is the bond angle, the equilibrium angle $\theta_0 = 5\pi/6$, and $K = 80.0 k_B T/rad^2$. To preserve the double-threaded structure, where each ring passes through two network strands, the two crosslinked beads from the removed crosslink, along with their two bonded neighboring beads, are temporarily constrained into a flat plane using an angular potential with an equilibrium angle $\theta_0 = \pi$. Once the slide ring is formed, this potential is deactivated. The normal vector of this plane is determined using the geometric center of these six beads. This center also serves as the reference point for generating the regular dodecagon structure of the slide ring within the plane, with a circumradius of 5σ. This slide-ring formation process is performed under the same NPT ensemble conditions, followed by an equilibration run for $1\times10^5\ \tau_{LJ}$.

(3) *Tangled Network*. The preparation of the tangled network follows the same process of randomly selecting a fraction of covalent crosslinking bonds for replacement, as in the steps for forming the SR-PCN. However, only bonds not connected to dangling ends are replaced to avoid topological changes during the process. Two dihedral angles are introduced to establish the double-thread "tangled" structure. These angles are defined using the two crosslinked beads from the removed crosslink, along with two connected beads from each thread. An improper dihedral potential is applied to enforce the desired "tangled" configuration:

$$U_{dihedral}(\phi) = K(\phi - \phi_0)^2 \quad \text{(S10)}$$

where the equilibrium dihedral angle is set to $\phi_0 = \pi/2$ and the force constant K is incrementally increased from 10 to 100 in steps of 20 every $5\times10^4\tau$ simulation run. Next, the bond connection from each crosslinked bead from the removed crosslink is exchanged with the two neighboring beads on the other threads to form the "tangled" structure (Figure 7a). The dihedral potential is applied during this step to stabilize the entangled configuration and is turned off before equilibration. The system is then subjected to an equilibration run for $1\times10^5\ \tau_{LJ}$, ensuring structural relaxation. The entire preparation process is carried out under the same NPT ensemble conditions, maintaining constant pressure $P = 0$ and constant temperature T set to unity.

(4) *No-Ring Network*. The preparation of the no-ring network follows the same process of randomly selecting a fraction of covalent crosslinking bonds, after which all the selected crosslinking bonds are removed. Then, the system is performed to an equilibration run for $1\times10^5\ \tau_{LJ}$ with the same NPT ensemble simulation run.

The mechanical properties of the network were evaluated by obtaining stress-elongation curves for each type of crosslinked gel, corresponding to covalent, SR-PCN, and tangled, through a series of uniaxial deformation simulations at constant volume.[19,20] In these simulations, a new deformation state was obtained by a series of small affine deformations $[x_i, y_i, z_i] \rightarrow [(1 + \Delta)^{-1/2}x_i, (1 + \Delta)^{-1/2}y_i, (1 + \Delta)z_i]$ with an increment $\Delta = 0.025$. Each small incremental deformation is achieved by deforming the network at a constant rate within $1.25 \times 10^3\tau_{LJ}$, followed by a $1 \times 10^4\ \tau_{LJ}$ run for equilibration and a $2.5 \times 10^4\ \tau_{LJ}$ run for the calculation of the average stress. The true stress is calculated as $\sigma_{\text{true}} = \frac{1}{2}(P_{xx} + P_{yy}) - P_{zz}$, where P_{ij} is the system pressure tensor as defined in LAMMPS.[18]

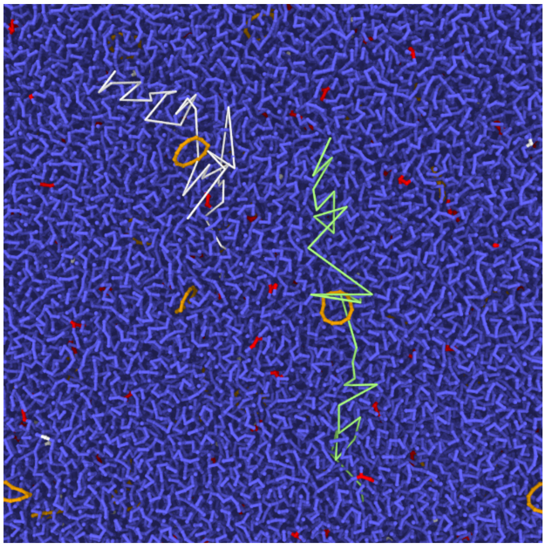

Figure S83. Zoomed-in trajectories of SR within the dtSR-PCN containing a 20% slide-ring fraction during uniaxial deformation (elongation ratio λ = 1.0–1.5).

Figure S83 illustrates the trajectories of slide rings in the dtSR-PCN with a 20% slide-ring fraction during uniaxial deformation from an elongation ratio of λ = 1.0 to 1.5. The green trail represents larger displacements than the white trail, showing slide-ring structures aligned with the deformation direction under tensile stress.

Storage modulus from small-amplitude oscillatory compression (SAOC) for $\mathbf{10}_{a/b}$ in NMP

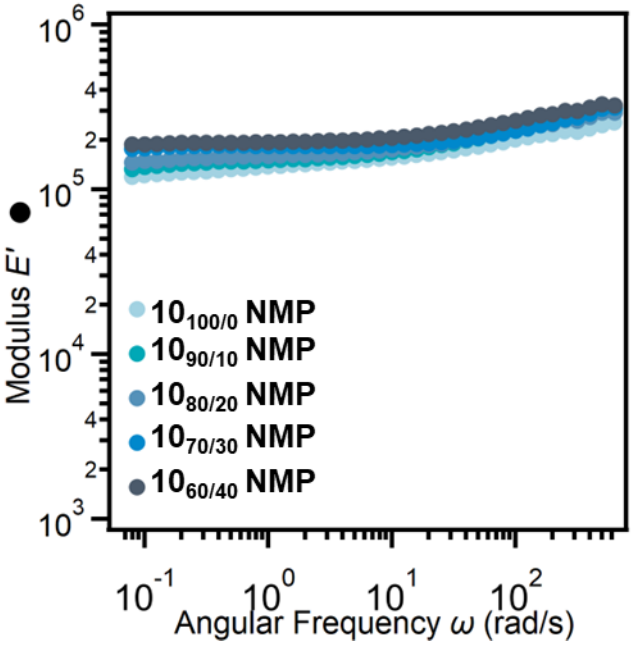

Figure S84. Storage modulus *E'* (filled circles) vs. angular frequency for control network ($\mathbf{10}_{a/b}$) swollen in NMP.

Dynamic light scattering (DLS) microrheology in NMP

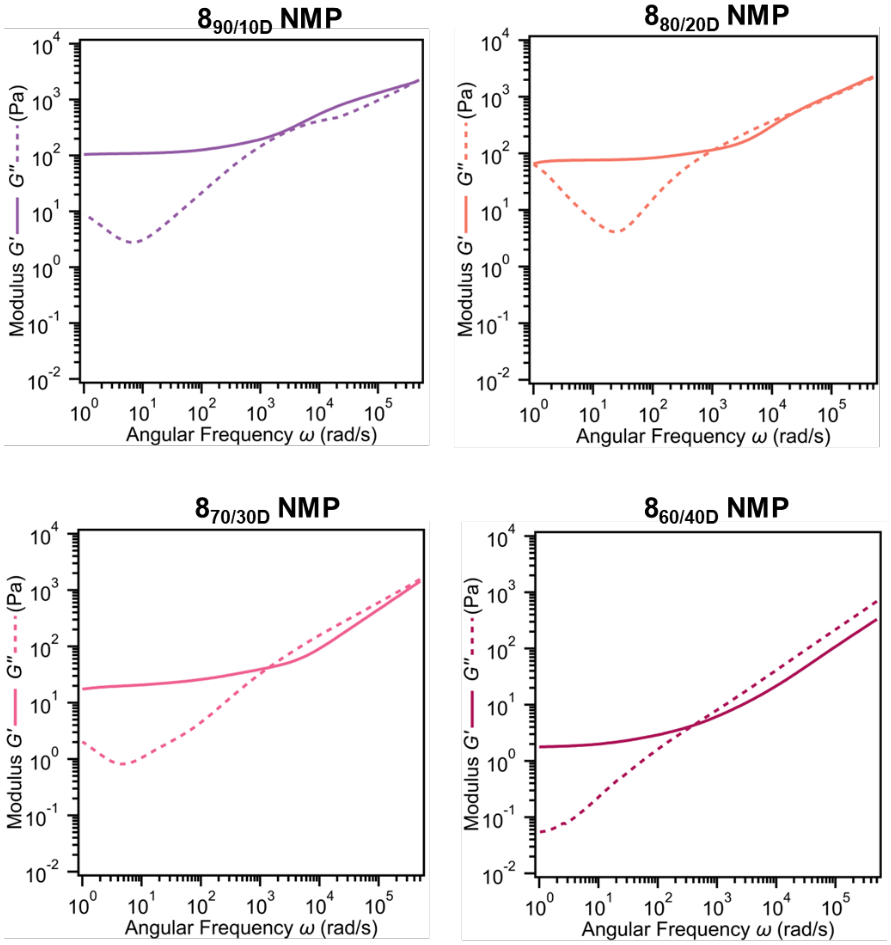

Figure S85. Dynamic light scattering (DLS) microrheology frequency sweeps showing storage modulus *G'* (solid lines) and loss modulus *G"* (dashed lines) for demetalated slide-ring polycatenane network (**8**$_{a/bD}$) swollen in NMP.

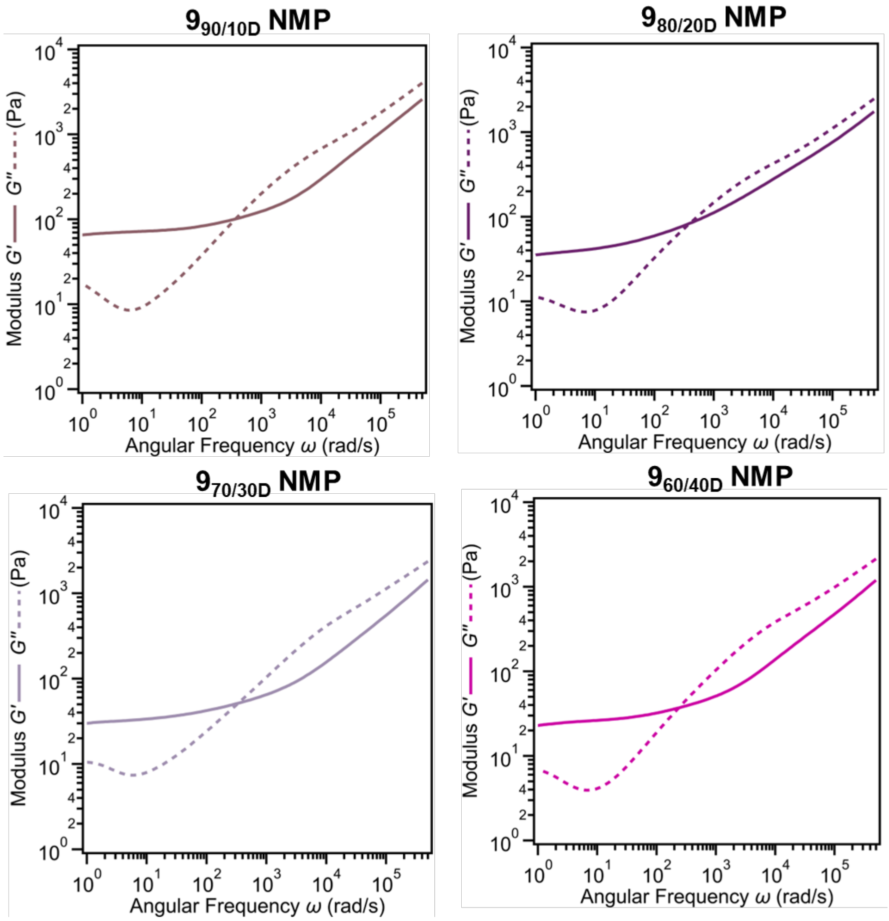

Figure S86. Dynamic light scattering (DLS) microrheology frequency sweeps showing storage modulus G' (solid lines) and loss modulus G'' (dashed lines) for demetalated control network ($\mathbf{9}_{\mathbf{a/bD}}$) swollen in NMP.

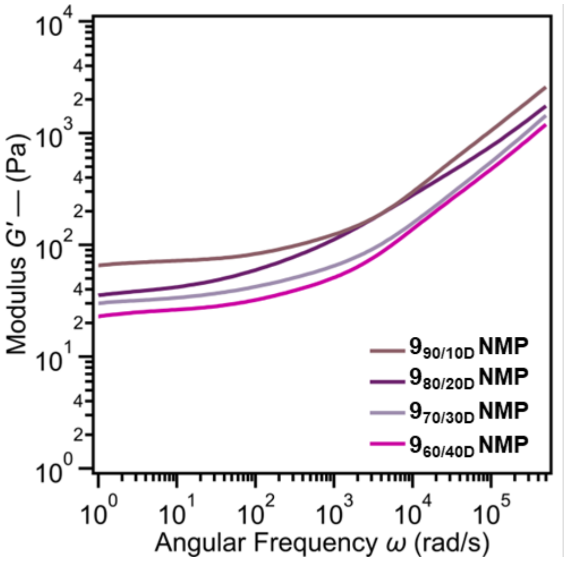

Figure S87. Storage modulus G (solid lines) vs. angular frequency for demetalated control network ($\mathbf{9}_{\mathbf{a/bD}}$) swollen in NMP.

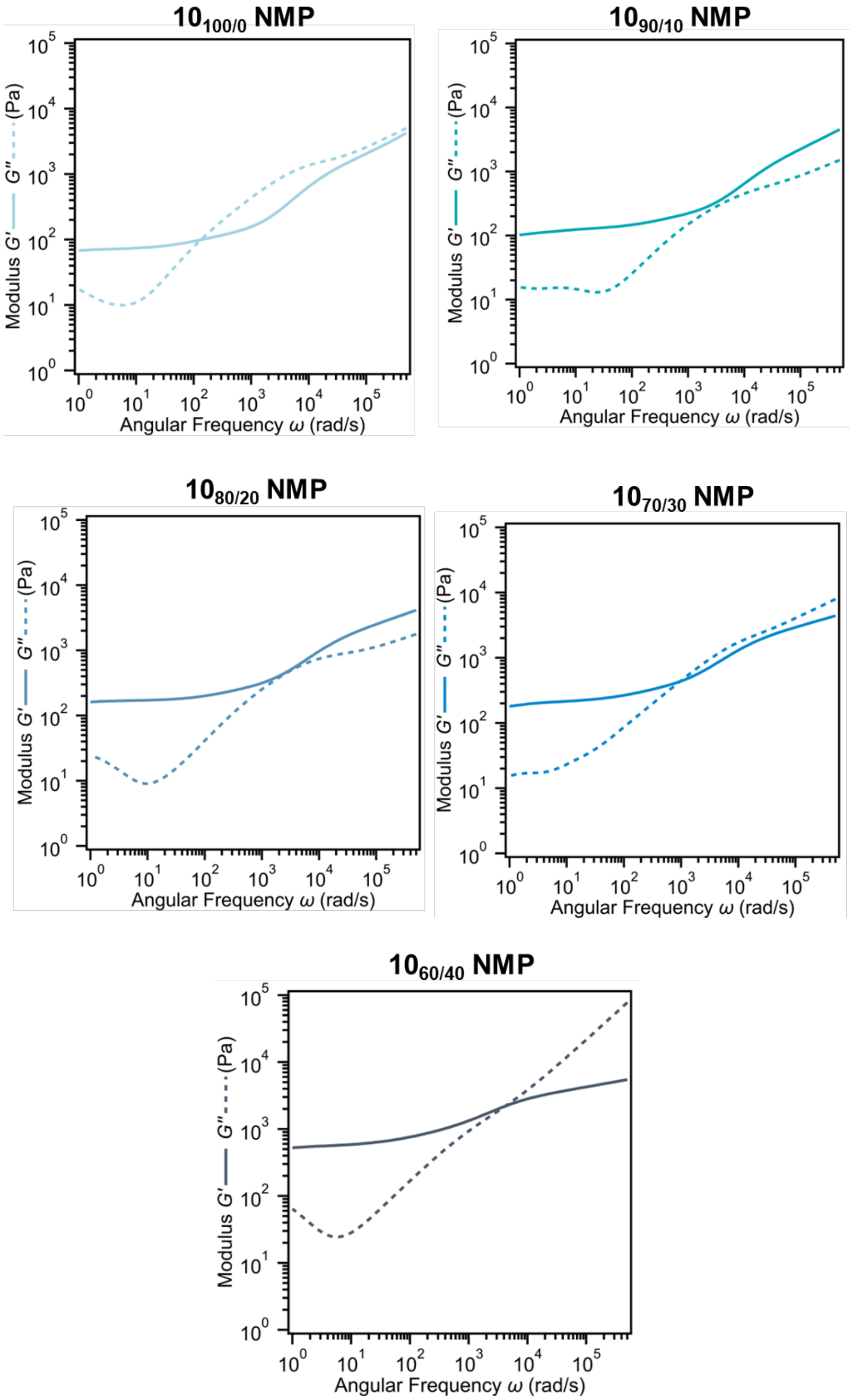

Figure S88. Dynamic light scattering (DLS) microrheology frequency sweeps showing storage modulus G' (solid lines) and loss modulus G'' (dashed lines) for the control network (**10**$_{a/b}$) swollen in NMP.

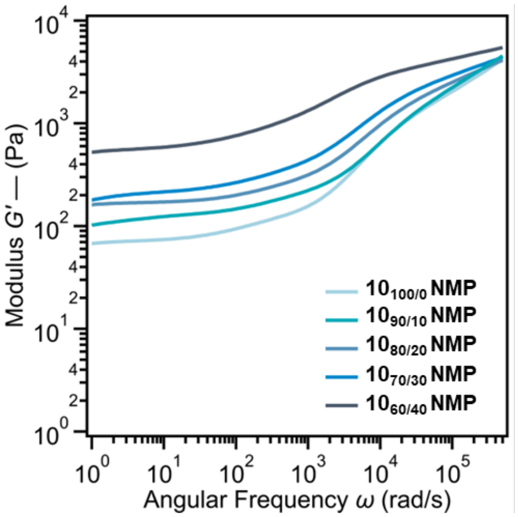

Figure S89. Storage modulus G' (solid lines) vs. angular frequency for control network (**10**$_{\mathbf{a/b}}$) swollen in NMP.

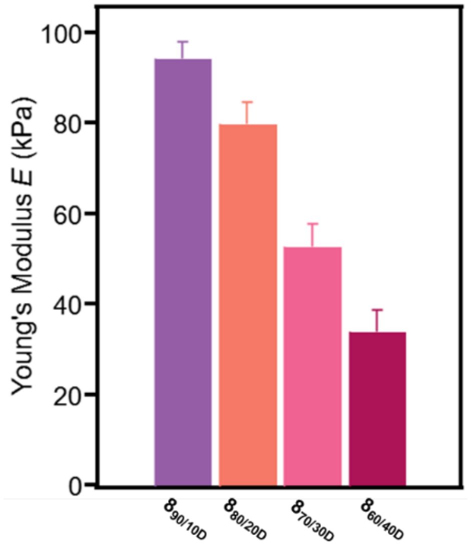

Figure S90. Young's modulus of swollen demetalated slide-ring polycatenane network (**8**$_{\mathbf{a/bD}}$) calculated from tensile testing with a strain rate of 20% strain min^{-1} until 200% strain before the material breaks. Young's modulus was determined by linear fitting of the stress-strain curves below 1% strain.

Computational Interpretation of Experimental Tensile Testing

To accurately replicate the experimentally observed stress-elongation behavior of doubly-threaded slide-ring polycatenane networks (dtSR-PCNs) within our computational framework, the simulations are conducted under NPT ensemble conditions, maintaining constant pressure $P = 2k_BT/\sigma^3$ and constant temperature T set to unity, controlled by the Nosé-Hoover barostat and thermostat. This methodology effectively modulates the network's swelling ratio (Q), thereby influencing its mechanical response.

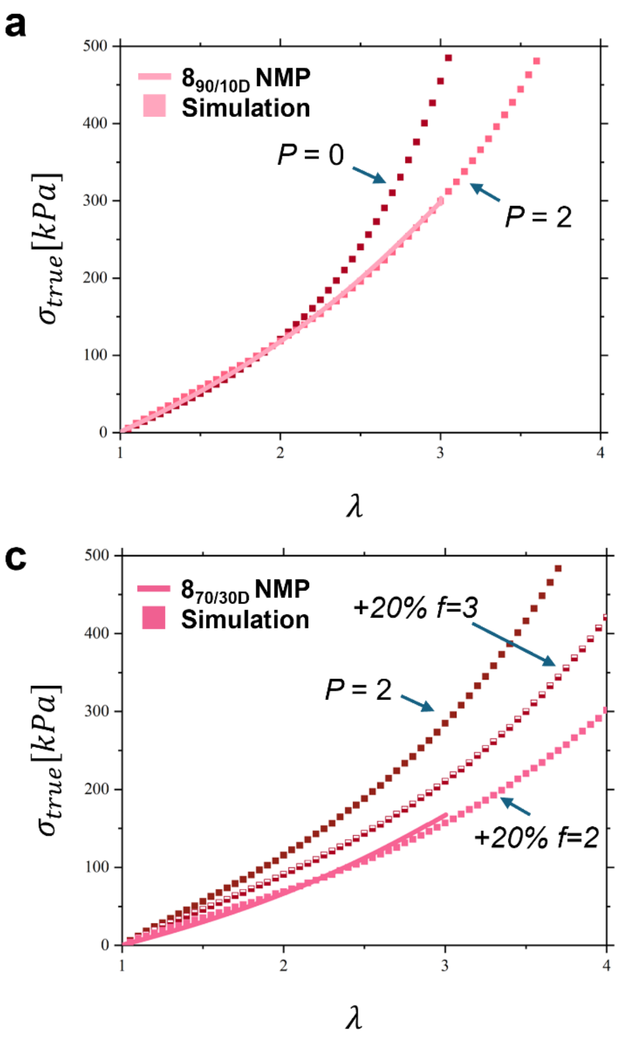

Figure S91. Dependence of the true stress σ_{true} on elongation ratio λ for various strategies employed to interpret the stress-elongation behavior of dtSR-PCNs, aligning simulation results with experimental data across different ring fractions of 10% (a), 20% (b), 30% (c), and 40% (d). A conversion of 1180 was applied to convert the LJ stress units into real stress units in kPa.

Figure S91 presents various simulation strategies implemented to interpret the mechanical behavior of dtSR-PCNs with ring fractions ranging from 10% to 40%. For the dtSR-PCN model with a 10% ring fraction, **Figure S91a** demonstrates a good agreement that the simulated stress–elongation behavior aligns well with experimental data when accounting for the swelling effects under the constant pressure to $P = 2k_BT/\sigma^3$. In case of the dtSR-PCN with a ring fraction of 20%, 12.5% of the total covalent crosslinks were modified by reducing their functionality (f) from

4 to 3, to achieve consistency between simulation and experiment (**Figure S91b**). This adjustment was accomplished by cutting the network strands at their midpoints, ensuring that each modified network crosslink can only have one dangling end with a degree of polymerization $n_x/2$ = 75, given n_x=150.[21] For the dtSR-PCN with a 30% ring fraction, to accommodate the increased presence of ring structures, the network topology was introduced by introducing more dangling ends compared with reducing 20% crosslink functionality from 4 to 3. Specifically, 20% of the covalent crosslinks were adjusted to an effective functionality from f = 4 to f = 2 by cutting two network strands at their centers. **Figure S91c** illustrates these systematic modifications applied to the network structure to align the simulated stress-strain behavior with experimental observations. At a ring fraction of 40%, simply changing crosslink functionality to f = 2 proved insufficient to replicate the experimental results, suggesting critical topological changes in the network structure due to the increased incorporation of slide rings. Consequently, after decreasing the functionality of 15% of the covalent crosslinks to f = 2, akin to what was done before, the crosslink density was further adjusted by randomly removing 20% of the covalent crosslinks. The crosslinks with reduced functionality were excluded from the removal process. As shown in **Figure S91d**, this approach resulted in a simulated stress-elongation curve that closely approximated the experimental behavior.

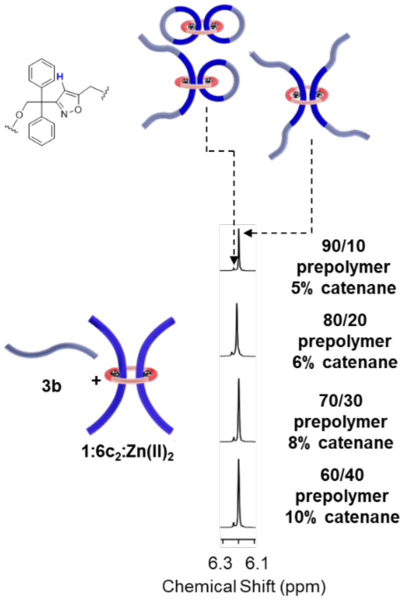

Figure S92. Crude ^{1}H-NMR (500 MHz, 25°C) spectra focusing on the isoxazole peaks in the catenane and rotaxane products in reactions of varying amounts of nitrile-oxide monomer **3b** and P3R **1:6c$_2$:Zn(II)$_2$** in 5% acetonitrile-d_3 in chloroform-d.

Pictures of organogel $\mathbf{8}_{\mathbf{80/20D}}$

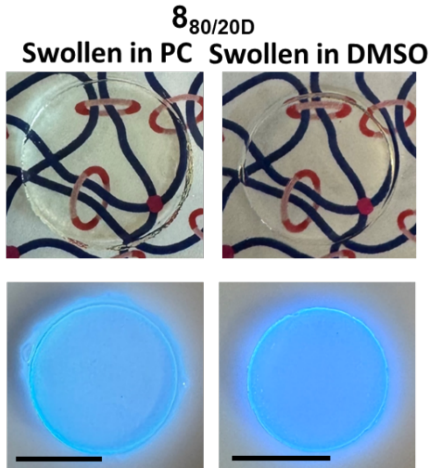

Figure S93. Pictures of $\mathbf{8}_{\mathbf{80/20D}}$ in DMSO and PC under ambient light (top) and under 365 nm UV light (bottom), 1 cm scale bar.

Storage and loss moduli from small-amplitude oscillatory compression (SAOC) in different solvents and remetalation

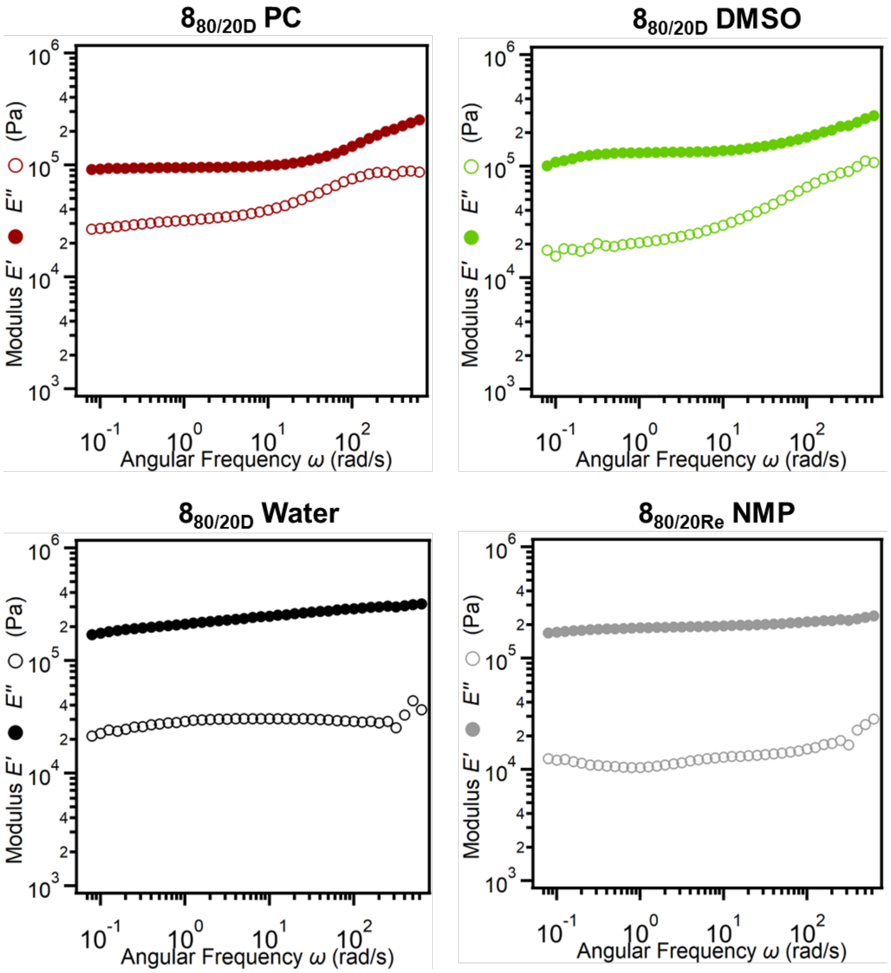

Figure S94. Small-amplitude oscillatory compression (SAOC) frequency sweeps showing storage modulus *E'* (filled circles) and loss modulus *E''* (open circles) for demetalated slide-ring polycatenane network (**$8_{80/20D}$**) swollen in different solvents and remetalated slide-ring polycatenane network (**$8_{80/20Re}$**) swollen in NMP.

Fluorescence spectrometry

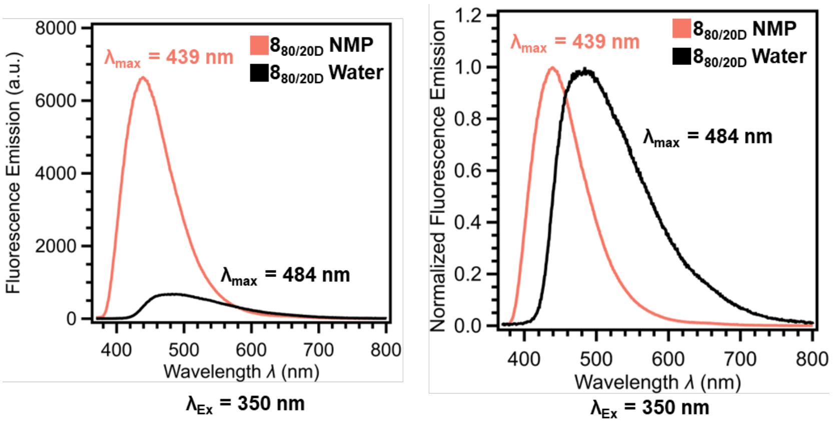

Figure S95. Fluorescence (excitation wavelength: 350nm) spectra of the demetalated SR-PCN **8**$_{\mathbf{80/20D}}$ hydrogel and organogel swollen in NMP. Swollen hydrogel gel was cut into a rectangular shape and adhered in a quartz 2mm x 10 mm cuvette from Hellma. After measuring the fluorescence of the hydrogel, the cuvette with the hydrogel was fully dried, and the same network in the cuvette was fully swollen in NMP and measured again. (Left: raw data of the same gel in water and NMP, Right: Normalized data of the same gel in water and NMP)

Remetalation procedure

To remetalate, the NMP swollen network **8**$_{\mathbf{80/20D}}$ was added to chloroform (200 mL) to soak for 12 h. This process was repeated six times. The chloroform swollen network was transferred to a Teflon dish to dry in a fume hood overnight. Then, dry network was dried in vacuo (48 h) at 50 °C. The network was then submerged in a solution of zinc di[bis(trifluoromethylsulfonyl)imide] (1.88 mg, 0.00301 mmol) in acetonitrile (15 mL) for 24 h to access **8**$_{\mathbf{80/20Re}}$**.** The acetonitrile swollen remetalated network **8**$_{\mathbf{80/20Re}}$ was transferred to a Teflon dish to dry in a fume hood overnight. Then, dry network was dried in vacuo (48 h) at 50 °C. The network was then submerged in NMP (50 mL) to allow it to reach its swelling equilibrium.

Acidification procedure

To acidify, the NMP swollen network **8**$_{\mathbf{80/20D}}$ was submerged in a solution of 0.1 M aq. hydrochloric acid solution (0.015 mL) in NMP (15 mL) for 24 h to access **8**$_{\mathbf{80/20H+}}$**.** This process was then repeated to access **8**$_{\mathbf{80/202H+}}$**.** Next, to acidify excessively, the NMP swollen network **8**$_{\mathbf{80/202H+}}$ was submerged in a solution of 0.1 M aq. hydrochloric acid solution (0.12 mL) in NMP (15 mL) for 24 h to access **8**$_{\mathbf{80/20exH+}}$**.**

Storage and loss moduli from small-amplitude oscillatory compression (SAOC) after acidification in NMP

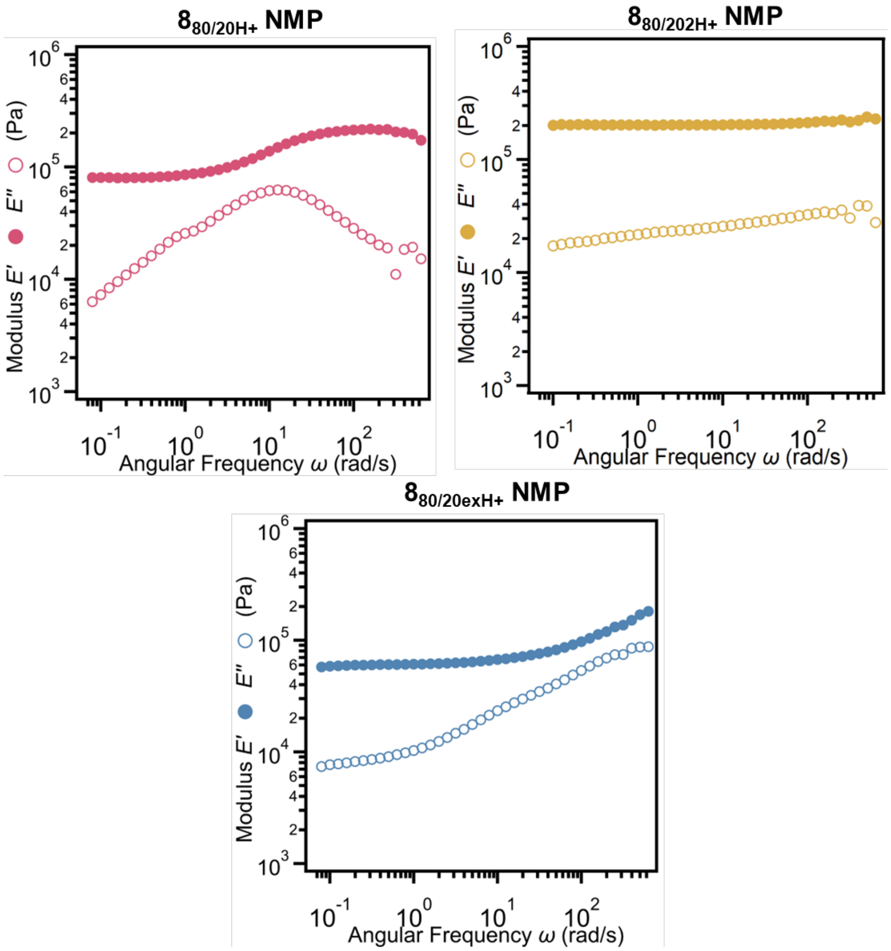

Figure S96. Small-amplitude oscillatory compression (SAOC) frequency sweeps showing storage modulus *E'* (filled circles) and loss modulus *E''* (open circles) for slide-ring polycatenane network (**$8_{80/20}$**) swollen in NMP with different amounts of acid.

References

1 B. M. McKenzie, A. K. Miller, R. J. Wojtecki, J. C. Johnson, K. A. Burke, K. A. Tzeng, P. T. Mather and S. J. Rowan, *Tetrahedron*, 2008, **64**, 8488–8495.

2 T. Tsutsuba, H. Sogawa, S. Kuwata and T. Takata, *Chem. Lett.*, 2017, **46**, 315–318.

3 A. Jerschow and N. Müller, *J. Magn. Reson. A.*, 1996, **123**, 222–225.

4 A. Jerschow and N. Müller, *J. Magn. Reson.*, 1997, **125**, 372–375.

5 P. Ziegler, *Bruker Topspin Advanced NMR Methods User Manual*, Bruker Topspin, 2010.

6 P. Ziegler, *1D and 2D Experiments Step-by-step Tutorial; Advanced Experiments User Guide, vers. 002*, Bruker Biospin, 2006.

7 P. C. Cai, B. A. Krajina, M. J. Kratochvil, L. Zou, A. Zhu, E. B. Burgener, P. L. Bollyky, C. E. Milla, M. J. Webber, A. J. Spakowitz and S. C. Heilshorn, *Soft Matter*, 2021, **17**, 1929–1939.

8 J. E. Hertzog, V. J. Maddi, L. F. Hart, B. W. Rawe, P. M. Rauscher, K. M. Herbert, E. P. Bruckner, J. J. de Pablo and S. J. Rowan, *Chem. Sci.*, 2022, **13**, 5333–5344.

9 V. M. Cangelosi, A. C. Sather, L. N. Zakharov, O. B. Berryman and D. W. Johnson, *Inorg. Chem.*, 2007, **46**, 9278–9284.

10 L. F. Hart, W. R. Lenart, J. E. Hertzog, J. Oh, W. R. Turner, J. M. Dennis and S. J. Rowan, *J. Am. Chem. Soc.*, 2023, **145**, 12315–12323.

11 C.-G. Wang, Y. Koyama, M. Yonekawa, S. Uchida and T. Takata, *Chem. Commun.*, 2013, **49**, 7723–7725.

12 O. Michel and B. J. Ravoo, *Langmuir*, 2008, **24**, 12116–12118.

13 A. E. Rodda, F. Ercole, V. Glattauer, J. Gardiner, D. R. Nisbet, K. E. Healy, J. S. Forsythe and L. Meagher, *Biomacromolecules*, 2015, **16**, 2109–2118.

14 O. Norberg, L. Deng, M. Yan and O. Ramström, *Bioconjugate Chem.*, 2009, **20**, 2364–2370.

15 H. S. Gill, J. N. Tinianow, A. Ogasawara, J. E. Flores, A. N. Vanderbilt, H. Raab, J. M. Scheer, R. Vandlen, S.-P. Williams and J. Marik, *J. Med. Chem.*, 2009, **52**, 5816–5825.

16 K. Zhang, W. Feng and C. Jin, *MethodsX*, 2020, **7**, 100779.

17 K. Kremer and G. S. Grest, *J. Chem. Phys.*, 1990, **92**, 5057–5086.

18 A. P. Thompson, H. M. Aktulga, R. Berger, D. S. Bolintineanu, W. M. Brown, P. S. Crozier, P. J. in 't Veld, A. Kohlmeyer, S. G. Moore, T. D. Nguyen, R. Shan, M. J. Stevens, J. Tranchida, C. Trott and S. J. Plimpton, *Comput. Phys. Commun.*, 2022, **271**, 108171.

19 A. V Dobrynin, M. Jacobs and Y. Tian, *Macromolecules*, 2023, **56**, 9289–9296.

20 E. R. Duering, K. Kremer and G. S. Grest, *J. Chem. Phys.*, 1994, **101**, 8169–8192.

21 A. V Dobrynin, Y. Tian, M. Jacobs, E. A. Nikitina, D. A. Ivanov, M. Maw, F. Vashahi and S. S. Sheiko, *Nat. Mater.*, 2023, **22**, 1394–1400.
